# Refinement of the CS6-expressing enterotoxigenic *Escherichia coli* strain B7A human challenge model: A randomized trial

**DOI:** 10.1371/journal.pone.0239888

**Published:** 2020-12-02

**Authors:** Kawsar R. Talaat, Chad K. Porter, Kayla M. Jaep, Christopher A. Duplessis, Ramiro L. Gutierrez, Milton Maciel, Brittany Adjoodani, Brittany Feijoo, Subhra Chakraborty, Jessica Brubaker, Stefanie A. Trop, Mark S. Riddle, Sabrina S. Joseph, A. Louis Bourgeois, Michael G. Prouty

**Affiliations:** 1 Department of International Health, Johns Hopkins University Bloomberg School of Public Health, Baltimore, MD, United States of America; 2 Naval Medical Research Center, Silver Spring, MD, United States of America; 3 Henry M. Jackson Foundation, Bethesda, MD, United States of America; 4 Uniformed Services University of the Health Sciences, Bethesda, MD, United States of America; 5 PATH, Washington, DC, United States of America; IAVI, UNITED STATES

## Abstract

**Background:**

Human challenge models for enterotoxigenic *Escherichia coli* (ETEC) facilitate vaccine down-selection. The B7A (O148:H28 CS6^+^LT^+^ST^+^) strain is important for vaccine development. We sought to refine the B7A model by identifying a dose and fasting regimen consistently inducing moderate-severe diarrhea.

**Methods:**

An initial cohort of 28 subjects was randomized (1:1:1:1) to receive B7A following an overnight fast at doses of 10^8^ or 10^9^ colony forming units (cfu) or a 90-minute fast at doses of 10^9^ or 10^10^ cfu. A second cohort included naïve and rechallenged subjects who had moderate-severe diarrhea and were given the target regimen. Immune responses to important ETEC antigens were assessed.

**Results:**

Among subjects receiving 10^8^ cfu of B7A, overnight fast, or 10^9^ cfu, 90-minute fast, 42.9% (3/7) had moderate-severe diarrhea. Higher attack rates (71.4%; 5/7) occurred in subjects receiving 10^9^ cfu, overnight fast, or 10^10^ cfu, 90-minute fast. Upon rechallenge with 10^9^ cfu of B7A, overnight fast, 5/11 (45.5%) had moderate-severe diarrhea; the attack rate among concurrently challenge naïve subjects was 57.9% (11/19). Anti-CS6, O148 LPS and LT responses were modest across all groups.

**Conclusions:**

An overnight fast enabled a reduction in the B7A inoculum dose; however, the attack rate was inconsistent and protection upon rechallenge was minimal.

## Introduction

Infectious diarrhea causes significant morbidity and mortality in infants, young children, and vulnerable populations in resource-limited countries, with an estimated 2.4 billion annual cases and 1.3 million deaths [1]. Enterotoxigenic *Escherichia coli* (ETEC) are a major cause of diarrhea in this population and among travelers to endemic regions [2–5]. Colonization factors (CFs) are critical ETEC virulence factors, and of the approximately 25 known ETEC CFs, CS6 is one of the most epidemiologically prevalent and considered a necessary component in a multivalent vaccine targeting the CFs [6–8].

The controlled human infection model (CHIM) is a unique opportunity to better understand disease pathogenesis and host immune responses in addition to providing a framework to assess the efficacy of prevention or treatment modalities [9]. The ETEC CHIM is one of the most well studied controlled infections [10]. Since the seminal publication in 1971, it has been instrumental in defining ETEC as a cause of secretory diarrhea [11]; approximately 1,000 subjects have been administered ETEC in experimental infection studies [10, 12, 13]. One of the most commonly administered strains is B7A, a CS6-expressing, LT/ST-positive strain, which has been safely administered at doses ranging from 1 x 10^6^ to 1 x 10^10^ colony forming units (cfu) with diarrhea rates ranging from 0% to 100% [10]. Over a decade ago, Coster et al administered 10^9^ and 10^10^ cfu with diarrhea rates of 63% and 100%, respectively [14].

One concern regarding the ETEC CHIM is the inoculum dose required to induce sufficient disease rates to facilitate evaluation of vaccine efficacy in reasonable numbers of subjects [12]. A high B7A inoculum may not be reflective of the inoculum dose in natural exposures and may bias efficacy estimates towards the null. Harro et al attempted to address this concern in a different ETEC strain, H10407, by changing the buffer and extending the fasting period before challenge, thereby reducing the required inoculum for the H10407 strain by two logs [12]. Based on these promising results, we sought to re-establish the B7A model using the most recently utilized doses (10^9^, 10^10^ cfu) and fasting regimen (90 minutes) while simultaneously assessing the effect of an overnight fast on moderate-severe diarrhea rates at 10^8^ and 10^9^ cfu doses.

## Methods

This was a dose-finding study in which the ETEC strain B7A (O148:H28 CS6^+^ LT^+^ST^+^) was orally administered to healthy subjects to identify a dose and fasting regimen that induced moderate-severe diarrhea in ≥70% of subjects. Additionally, we sought to assess protection upon repeat exposure to the B7A strain. An initial cohort was designed as an open-label study in which B7A was simultaneously administered to 28 subjects randomized in a 1:1:1:1 fashion to three dose levels (10^8^, 10^9^, 10^10^ cfu) with either an overnight (10^8^, 10^9^ cfu) or 90-minute (10^9^, 10^10^ cfu) fast (subjects randomized in blocks of 4; see randomization procedure, Supplementary materials). A second, follow-on cohort was designed to include a minimum of 15 additional naïve subjects at the selected target inoculum and fasting time along with a rechallenge of up to 15 previously exposed subjects who met the primary endpoint in the initial cohort. Rechallenged subjects also received the selected target inoculum dose at a comparable fasting time.

Healthy adult subjects aged 18–50 years were recruited from Baltimore, Maryland and surrounding areas, screened, and enrolled; the study was conducted April- November 2016. Informed consent was conducted through thorough conversation with the participants, including a written assessment to ensure comprehension. A written informed consent was obtained from male and non-pregnant female volunteers who were evaluated to assure good health and eligibility through medical history, physical examination, and clinical laboratory tests (Supplement, Eligibility Criteria). The day prior to inoculation, subjects were admitted to the Johns Hopkins University (JHU) Center for Immunization Research Bayview facility and remained in the inpatient unit until meeting all discharge criteria. Following discharge, subjects returned for at least one outpatient visit to assess for new adverse events and to have samples collected for immunology testing. Additionally, approximately six months following experimental infection, subjects were contacted by telephone to assess for new onset serious adverse events.

The Western Institutional Review Board (IRB) and the Naval Medical Research Center IRB reviewed and approved the protocol prior to initiation in compliance with all applicable Federal regulations governing the protection of human subjects. The study was registered on ClinicalTrials.gov as NCT02773446.

### Challenge preparation and dosing

The B7A strain, Lot 0481, was manufactured using Good Manufacturing Practice at the Walter Reed Army Institute of Research Pilot Bioproduction Facility [10, 11, 14, 15]. Each vial of the production cell bank contains approximately 9 x 10^8^ cfu of live B7A in Luria Broth (LB) with 15% glycerol as cryopreservative. Vials were transferred to the JHU and stored at -80°C ± 10°C until use. Approximately 48 hours prior to inoculation, a single vial was thawed, vortexed and plated for isolation onto six colonization factor antigen (CFA) agar plates (without bile salts) [16]. The plates were incubated for 22–24 hours at 37°C ± 1°C. From these plates, five isolated *E*. *coli*-like colonies were first screened for agglutination in anti—B7A antisera and if results were positive, 12 additional like-appearing colonies were selected and suspended in 3 mL sterile saline (0.9%). Using a calibrated pipette, 0.1 mL of the suspension was plated on each of seven CFA agar plates which were then incubated at 37°C. Cells were harvested in sterile saline after 18–20 hours. The final concentration of cfu’s was determined by optical density (OD) at 600 nm and confirmed by plate count. The OD of the suspension was adjusted to the appropriate concentration of bacterial cells. The inoculum dose for each challenge dose was determined by confirmatory plating on agar plates before and after administration. Additional internal quality checks on the challenge dose inoculum preparations used in these studies included determining the percentage of colonies expressing CS6 by colony blots using rabbit anti-CS6 antisera provided by NMRC and screening supernatants from the inoculum preparations for pre-formed ST toxin using previously published methods [17, 18] assessing cGMP level in T84 cells. The amount of ST present was determined relative to the amount of cGMP produced by 10 ng of purified ST-toxin (cGMP parameter assay kit from R&D Systems, Minneapolis, MN). ST toxin testing was performed courtesy of Tulane University. Greater than 90% of the B7A colonies recovered from these preparations expressed CS6 by colony blot and only negligible amounts of pre-formed ST toxin were detected in preparations.

Following the pre-specified fast, subjects drank 120 ml of sodium bicarbonate solution (1.6 gram of bicarbonate in 120 mL) just prior to ingesting 30 ml of the same sodium bicarbonate solution containing the ETEC inoculum. All subjects continued fasting for at least an additional 90 minutes post-inoculation.

### Subject monitoring

Subjects were regularly assessed for adverse events with vital sign measurements and focused physical exams. They were questioned daily for solicited adverse events, and this information was collected in a systematic manner. All stools were collected to assess for the primary endpoint of moderate-severe diarrhea. Subjects were given oral rehydration with passage of Grade 3–5 stool(s) to maintain proper hydration. Intravenous fluids were administered at the clinician’s discretion or when pre-specified criteria were met. All subjects were treated with ciprofloxacin (500 mg by mouth twice daily for three days) five days after infection or earlier if they met criteria for severe diarrhea based on volume, moderate diarrhea for 48 hours, mild or moderate diarrhea with two or more severe symptoms or any vomiting, or if a study physician determined it warranted. Subjects were discharged when clinical symptoms were resolved or resolving and the subject had two consecutive stool cultures negative for the challenge strain.

### Endpoints and definitions

The primary endpoint of moderate-severe diarrhea was defined as a subject with ≥4 loose stools or ≥401 grams of loose stools in a 24-hour period post-inoculation. Loose stools were those categorized as Grade 3–5 as previously described [19]. The primary endpoint was determined by an independent adjudication board comprised of three infectious disease physicians blinded to study group. Additional secondary clinical endpoints included maximum loose stool in any 24-hour interval, the proportion with severe diarrhea and/or other ETEC-attributable signs and symptoms, and overall ETEC disease severity as described by Porter et al [20].

### Immunology

#### Serum antibody ELISA

The serum levels of anti-CS6, -LT, and–O148 LPS IgG and IgA were measured by conventional ELISA methodologies on days 0, 7 and 28. Sera were assayed in duplicate for IgG and IgA anti-CS6, O148 LPS, and–heat labile toxin (LT). Antigen (Ag)-specific IgG ELISA was performed on Nunc™ MicroWell™, while Ag-specific IgA assay was performed on Nunc™ MicroWell™ Maxisorp™ 96-well plates (Thermo Scientific, Rochester, NY). Plates were coated with CS6 (WRAIR Bioproduction Facility, Silver Spring, MD, Lot 0840) at 1.0 μg/mL (IgG) or 2.0 μg/mL (IgA), while O148 LPS (NMRC Biochemistry Lab, Silver Spring, MD, Lot BB-210) was used at 2.5 μg/mL (IgG and IgA) all in carbonate buffer, for 1 hour at 37°C followed by overnight at 4°C. For Anti-LT assays, plates were initially coated overnight with 0.5 μg/mL GM1 (Sigma-Aldrich, Saint Louis, MO) in carbonate buffer, blocked with 1% casein (Sigma-Aldrich) and then coated with LT (Tulane University, New Orleans, LA) at 0.2 μg/mL (IgG and IgA) in carbonate buffer for 1 hour at 37°C. After three washes with Phosphate buffered saline with 0.05%Tween-20 (PBS-T), CS6, LPS, and LT plates were blocked with 200 μL/well of 5% non-fat milk (Sigma-Aldrich) in PBS-T, 2% casein, or 1% casein, respectively, for 1 hour at 37°C in a humidified chamber. After three washes with PBS-T, serum samples were added at a starting dilution of 1:50 in 1% non-fat milk-PBS-T, 2% casein or 1% casein, for CS6, LPS and LT assays, respectively. This was followed by a 3-fold serial dilution, then incubated for 1.5 hour at room temperature (RT) before plates were washed 5 times with PBS-T followed by addition of a secondary antibody. For IgG and IgA anti-CS6 and anti-LT, respectively, peroxidase-conjugated goat anti-human IgG (KPL, Gaithersburg, MD) or biotin-labeled goat anti-mouse IgA (KPL) were both added at 1:1000 in 1% non-fat milk-PBS-T or 1% casein buffer for 1.5 hours at 37°C in a humidified chamber. For IgG and IgA anti-LPS assays, biotin goat anti-human IgG or IgA (Jackson ImmunoResearch Labs, West Grove, PA) in 2% casein buffer were added for 1.5 hours at RT. After further washes, 1:2000 ExtrAvidin^®^-peroxidase (Sigma-Aldrich) was added to IgA as well as IgG anti-LPS plates for 1.5 hours at RT. After washes, IgG anti-CS6 and anti-LT plates were developed by addition of 2,2’-azino-bis(3-ethylbenzothiazoline-6-sufphonic acid (ABTS) substrate (KPL) for 30 min at RT, while IgA assays were developed with 1-Step Ultra TMB (3,3',5,5'-tetramethylbenzidine; Fisher Scientific, Waltham, MA) for 30 min at RT and stopped according to manufacturer directions [21].

After incubation, optical density (OD) was measured at 405 (ABTS) or 450 nm (Ultra TMB) using a Multiskan EX^®^ ELISA reader with Ascent^®^ software (Thermo Scientific). Cut-off for each plate was calculated by the average of the background wells OD plus a fixed value of 0.4.

ELISA results were calculated by a linear regression fitted to the experimental data; the endpoint titer was determined as the reciprocal of the interpolated sample dilution that intersected with the cutoff and was log10-transformed for analyses. The average of duplicate assays was calculated as the final result. Sera with OD below the cutoff were assigned a value of one half of the initial dilution (i.e., 1:25) for computational purposes as previously described [21]. Subjects with ≥4-fold increase in titers over baseline were considered responders.

#### Antibody in Lymphocyte Supernatant (ALS)

ALS were prepared from Peripheral Blood Mononuclear Cells (PBMCs) collected on study days 0 and 7. Fresh isolated PBMC were resuspended in RPMI (Life technologies, Carlsbad, CA) containing 10% fetal calf serum (Life Technologies), 1% Penicillin-Streptomycin and 1% GlutaMax (Life Technologies), and incubated in 24-well plates (Corning Inc., Corning, NY) at 5x10^6^ cells/ml, 1 ml/well, at 37°C and 5% CO_2_ for 72 h. Following incubation, culture supernatants were collected and stored at −80°C until tested by ELISA for the measurement of IgG and IgA anti-CS6, -LPS and -LT antibodies, using the methodologies described above with minor modifications. The initial dilutions for anti-CS6, -LPS and LT were 1:5, 1:8 and 1:8, respectively. Serum samples with OD below the cutoff, even at the lowest dilution, were assigned a value of one-half of the initial dilution (i.e. 1:2.5, 1:4 or 1:4) for computational purposes. Subjects with ≥4-fold increase in titers over baseline were considered responders.

### Microbiology

Up to three stool samples per day following challenge were plated directly on MacConkey agar (Mac) and on Mac supplemented with 25 μg/ml of chloramphenicol (Mac+CM). The B7A strain is naturally chloramphenicol resistant and the use of Mac+CM allows for differentiation of B7A from commensal *E*. *coli* strains. Rectal swabs were collected when subjects were unable to produce a stool sample on any given day. After incubation for 18–24 hours at 37°C, up to five lactose fermenting, *E*. *coli*-like colonies on Mac+CM agar were screened for slide agglutination with rabbit anti-B7A whole cell antiserum. If no B7A positive colonies were found on the Mac+CM agar, an additional five colonies were screened from the Mac plates.

For quantitative cultures two and four days post-inoculation, approximately 1 g of each collected stool sample was serially diluted in PBS and plated on Mac and Mac+CM plates. After 18–24 hours of incubation at 37°C, five presumptive *E*. *coli* colonies were screened for agglutination with anti-B7A antiserum. Based on the percentage of positive reactions, the cfu's of B7A per gram of stool were estimated. If no B7A positive colonies were found on the Mac+CM plates, then additional colonies were screened from the Mac plates. Quantitative shedding on a subject level was discontinued after initiation of antibiotic treatment.

### Statistical analysis

A sample size of 15 subjects per arm in Cohort 2 provided an 80% power to detect a moderate-severe diarrhea risk difference of 55% presuming a ≥70% attack rate in naïve subjects. The proportion of subjects meeting various endpoints was compared using Fisher's exact test. Continuous variables were compared using the Kruskal-Wallis test. All statistical tests were interpreted in a two-tailed fashion using an *alpha* = 0.05. This study was not powered to demonstrate significant differences in the rate of moderate-severe diarrhea across B7A doses and fasting periods.

## Results

### Subjects

**Fig 1:**
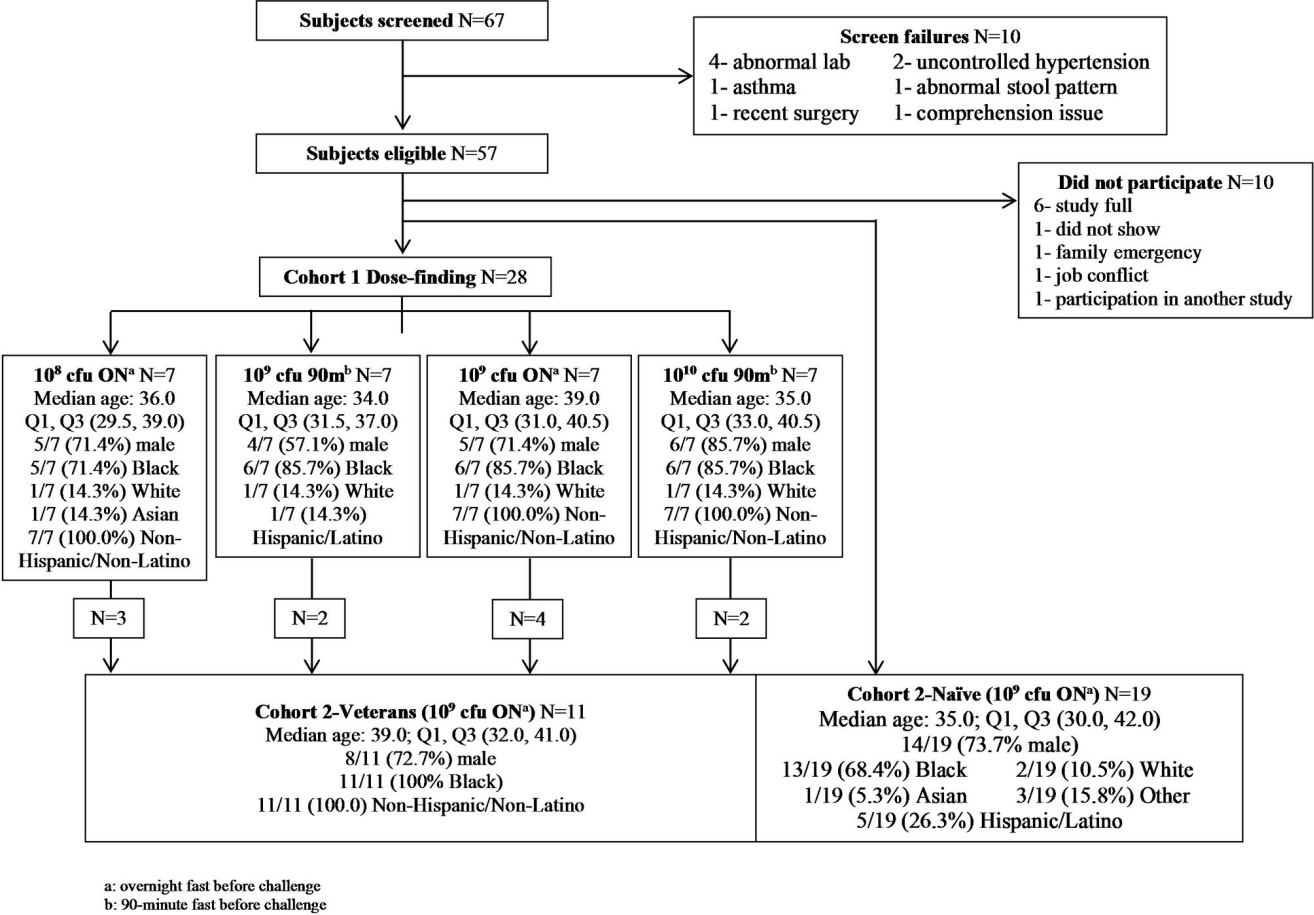
Subject disposition.

Twenty-eight subjects were randomized to one of the four dosing groups for the first cohort. Nineteen additional subjects were enrolled as naïve in the second cohort, for a total of 47 subjects. Participants were predominately male (34/47, 72%) and African-American (37/47, 78%) with a median age of 35.0 (IQR: 30.5, 40.0) (Fig 1). There were no differences in the demographic characteristics in subjects across dosing groups.

### Clinical symptoms

Among subjects in the groups receiving B7A doses of 10^8^ and 10^9^ cfu following a 90-minute fast, three of seven (42.9%) from each group met the primary endpoint of moderate-severe diarrhea (Table 1). Stool output was also comparable with a maximum 24-hour output of approximately 250 g for the aforementioned study groups. The rate of moderate-severe diarrhea was higher, 71.4% (5/7), in groups receiving 10^9^ cfu following overnight fast or 10^10^ after a 90-minute fast. The group receiving 10^9^ cfu following overnight fast had the highest maximum 24-hour output based on frequency (median 8; interquartile range (IQR): 2, 10) and the greatest volume (median: 1055 g; IQR: 380, 1339); however, there were no significant differences in output across all study groups (p>0.05). The shortest time to the onset of diarrhea (median: 11.4 h; IQR: 11.2, 13.4) and shortest duration of diarrhea (median 22.6 h; IQR: 16.0, 70.8) were observed in the group receiving 10^9^ cfu following 90-minute fast.

**Table 1:** Clinical outcomes by group.

	Cohort 1				Cohort 2- Naive	Cohort 2- Veteran
**Target Dose (cfu)**	**1.0x10**^**8**^	**1.0x10**^**9**^	**1.0x10**^**9**^	**1.0x10**^**10**^	**1.0x10**^**9**^	**1.0x10**^**9**^
**Actual Dose (cfu)**	**1.66x10**^**8**^	**1.90x10**^**9**^	**1.90x10**^**9**^	**1.97x10**^**10**^	**1.90x10**^**9**^	**1.90x10**^**9**^
**Fasting Time**	**Overnight**	**90 min**	**Overnight**	**90 min**	**Overnight**	**Overnight**
**MSD (any)**	3/7 (42.9%)	3/7 (42.9%)	5/7 (71.4%)	5/7 (71.4%)	11/19 (57.9%)	5/11 (45.5%)
**MSD (by vol)**	3/7 (42.9%)	3/7 (42.9%)	5/7 (71.4%)	5/7 (71.4%)	11/19 (57.9%)	5/11 (45.5%)
**MSD (by num)**	3/7 (42.9%)	2/7 (28.6%)	4/7 (57.1%)	5/7 (71.4%)	10/19 (52.6%)	4/11 (36.4%)
**Max 24 hr vol, median (IQR)**	263 (0, 1366)	252 (0, 1046)	1055 (380, 1339)	607 (247, 708)	504 (135, 766)	303 (0, 1016)
**Max 24 hr num, median (IQR)**	3 (0, 6.0)	1 (0, 13)	8 (2, 10)	5 (2, 10)	5 (1, 6)	3 (0, 6)
**Onset time (h), median (IQR)**	31.0 (16.0, 46.0)	11.4 (11.2, 13.4)	18.5 (13.0, 21.4)	24.8 (12.0, 43.1)	14.2 (9.5, 17.4)	12.8 (5.2, 18.7)
**MSD time (h), median (IQR)**	36.1 (21.0, 80.2)	22.6 (16.0, 70.8)	28.8 (20.2, 29.1)	34.5 (26.4, 66.9)	24.7 (13.6, 32.6)	23.1 (19.9, 24.1)
**IV therapy**	2/7 (28.6%)	2/7 (28.6%)	6/7 (85.7%)	3/7 (42.9%)	8/19 (42.1%)	2/11 (18.2%)
**Early abx**	3/7 (42.9%)	3/7 (42.9%)	4/7 (57.1%)	4/7 (57.1%)	10/19 (52.6%)	4/11 (36.4%)

cfu: colony forming units.

MSD: moderate to severe diarrhea.

Max 24 hr vol: maximum 24 hour volume of loose/liquid stools.

Max 24 hr num: maximum number of loose/liquid stools in 24 hour period.

IQR: interquartile range.

Abx: antibiotics.

Among 16 subjects meeting the moderate-severe diarrhea endpoint and subsequently eligible for Cohort 2, 11 were re-admitted to the inpatient unit 10 weeks following initial challenge (veteran subjects). An additional 19 naïve subjects were concurrently admitted. All subjects received 1.9 x 10^9^ cfu of B7A following an overnight fast. As shown in Table 1, the proportion of naïve subjects with moderate-severe diarrhea was lower than observed in the initial cohort with 11/19 (57.9%) meeting the primary endpoint. Additionally, the incidence of moderate-severe diarrhea in previously challenged subjects was not significantly different than in naïve subjects (5/11; 45.5%, p = 0.7). While the maximum 24-hour loose stool output appeared lower in the veteran subjects compared to naïve subjects, these differences were not statistically different (all p > 0.05). The number and percent of subjects with additional clinical signs and symptoms is shown in Table 2. Although most gastrointestinal and systemic symptoms occurred in volunteers who also experienced diarrhea, not all subjects with moderate-severe diarrhea had associated symptoms, and some subjects had symptoms without experiencing diarrhea.

**Table 2:** Frequency of adverse events by group.

	Group 1	Group 2	Group 3	Group 4	Naïve	Veteran
Dose	10^8^	10^9^	10^9^	10^10^	10^9^	10^9^
Fasting	Overnight	90 minutes	Overnight	90 minutes	Overnight	Overnight
N	7	7	7	7	19	11
Abdominal cramps, n (%)	5 (71.4)	5 (71.4)	5 (71.4)	3 (42.9)	13 (68.4)	7 (63.6)
Abdominal Pain, n (%)	3 (42.9)	4 (57.1)	5 (71.4)	5 (71.4)	12 (63.2)	7 (63.6)
Abdominal Tenderness, n (%)	0 (0.0)	0 (0.0)	2 (28.9)	0 (0.0)	9 (47.4)	2 (18.2)
Anorexia, n (%)	4 (57.1)	4 (57.1)	5 (71.4)	4 (57.1)	14 (73.7)	4 (36.4)
Arthralgia, n (%)	0 (0.0)	1 (14.3)	0 (0.0)	1 (14.3)	3 (15.8)	1 (9.1)
Bloating, n (%)	5 (71.4)	6 (85.7)	5 (71.4)	4 (57.1)	11 (57.9)	5 (45.5)
Constipation, n (%)	1 (14.3)	1 (14.3)	2 (28.9)	0 (0.0)	0 (0.0)	0 (0.0)
Fever, n (%)	0 (0.0)	0 (0.0)	1 (14.3)	0 (0.0)	3 (15.8)	1 (9.1)
Flatulence, n (%)	7 (100.0)	6 (85.7)	6 (85.7)	4 (57.1)	10 (52.6)	7 (63.6)
Generalized Myalgia, n (%)	0 (0.0)	1 (14.3)	0 (0.0)	1 (14.3)	3 (15.8)	2 (18.2)
Headache, n (%)	3 (42.9)	4(57.1)	3 (42.9)	5 (71.4)	10 (52.6)	4 (36.4)
Hyperactive Bowel Sounds, n (%)	5 (71.4)	2 (28.9)	7 (100.0)	3 (42.9)	8 (42.1)	3 (27.3)

**Fig 2:**
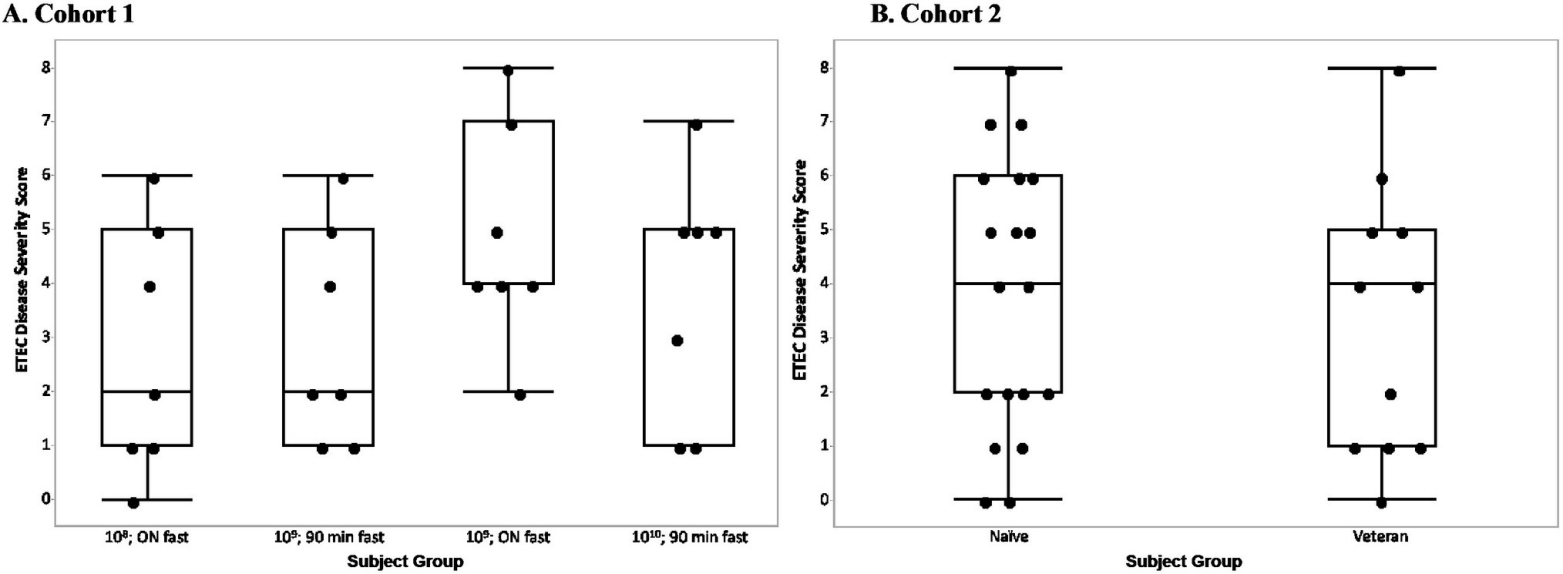
The disease severity score for each subject is represented by a dot on the figure based on (A) the dose and fasting time to which that subject was randomized in Cohort 1 or (B) veteran status of the subject in Cohort 2. The horizontal line within the box represents the median sample value. The ends of the box represent the 1st and 3rd quartile. The whiskers extend from the ends of the box to the outermost data point that falls within the distances computed as 1st/3rd quartile—1.5*(interquartile range).

In the initial cohort, the incorporation of an overnight fast doubled the median ETEC disease severity score in subjects receiving the 10^9^ cfu inoculum, although this was not statistically significant (p = 0.13) (Fig 2A). There was no difference in the median ETEC disease severity score between naïve and veteran subjects in Cohort 2 (p = 0.4); however, the lower and upper quartiles were slightly higher in naïve subjects corresponding to the higher attack rate in that group (Fig 2B).

### Microbiology

**Fig 3:**
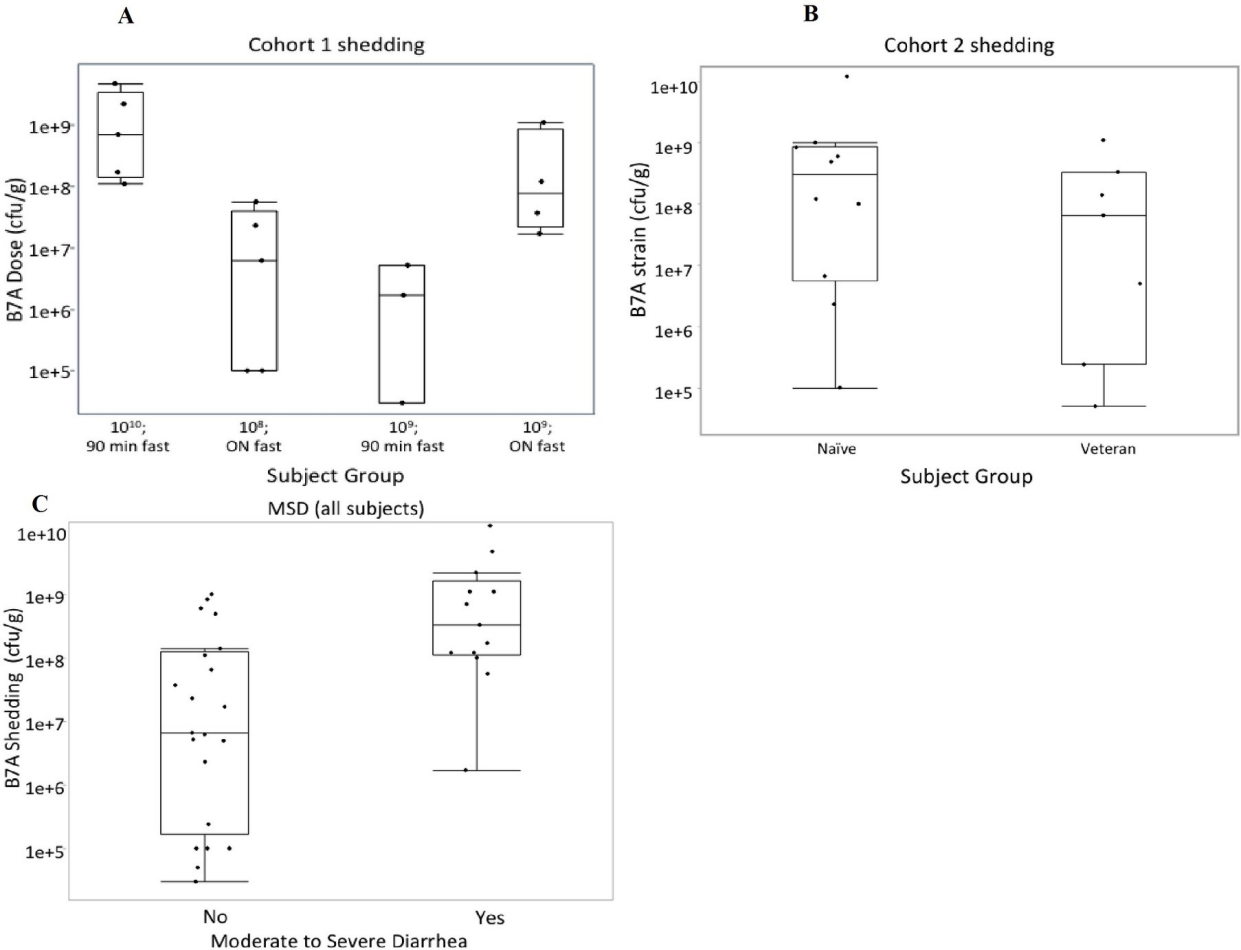
Comparison of median shedding 2 days after challenge with ETEC strain B7A based on (A) subjects in Cohort 1 (B) subjects in Cohort 2 and (C) subjects with and without moderate-severe diarrhea. The shedding value for each subject is represented by a dot on the figure based on (A) the dose/fasting time to which that subject was randomized, (B) veteran status, and (C) whether the subject experienced MSD. The horizontal line within the box represents the median sample value. The ends of the box represent the 1st and 3rd quartile. The whiskers extend from the ends of the box to the outermost data point that falls within the distances computed as 1st/3rd quartile—1.5*(interquartile range). Quantitative shedding was not evaluated for subjects who had initiated antibiotic treatment or did not produce a stool sample at the time point.

All subjects shed the challenge organism. Quantitative shedding was not evaluated for 11/28 subjects in Cohort 1 and 13/30 subjects in Cohort 2 at day 2 due to early antibiotic treatment (22/24) or collection of a rectal swab sample (2/24). Based on the day 2 shedding results, subjects receiving the 10^10^ cfu dose shed significantly more B7A than all other subjects (median: 7.0 x 10^8^ and 1.2 x 10^7^ cfu/g, respectively; p = 0.02) (Fig 3A). There was no significant difference in ETEC shedding between naïve and veteran subjects in Cohort 2 (median: 1.2 x 10^8^ and 1.0 x 10^8^ cfu/g, respectively; p = 0.7) (Fig 3B). Subjects with moderate-severe diarrhea shed approximately 50-fold more B7A than those that did not meet the primary outcome (median: 3.3 x 10^8^ cfu/g and 6.6 x 10^6^ cfu/g; p = 0.002) (Fig 3C).

### Immunology

**Fig 4:**
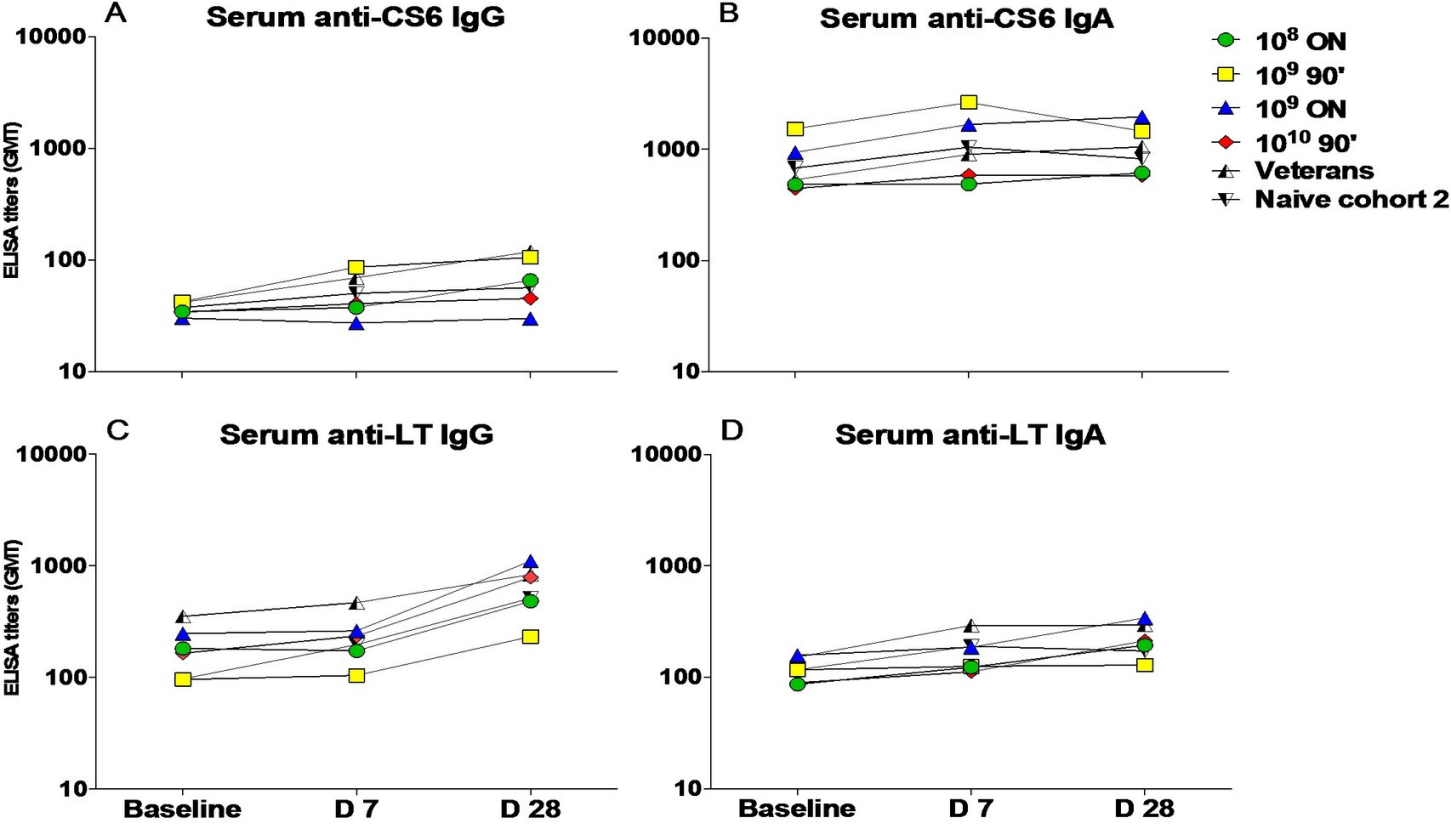
Serum IgG and IgA responses to CS6 and LT as measured by ELISA after B7A challenge. (A) IgG anti-CS6; (B) IgA anti-CS6; (C) IgG anti-LT; (D) IgA anti-LT. IgG and IgA antigen-specific responses were evaluated by ELISA before the oral challenge (baseline), and at 7 and 28 days later. Graphs represent GMT per time point per group. Cohort 1 groups divided by dose and fasting times. (ON- overnight, 90’- 90 minute). Cohort 2 divided into veterans and naïve.

**Fig 5:**
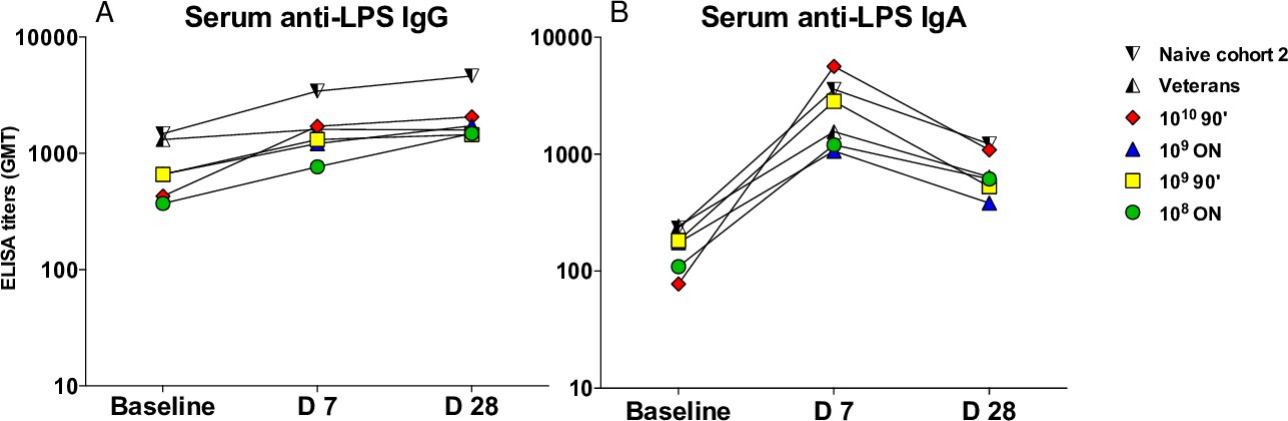
Serologic responses over time to LPS following B7A challenge. (A) IgG anti-LPS; (B) IgA anti- LPS. IgG and IgA antigen-specific responses were evaluated by ELISA before the oral challenge (baseline), and 7 and 28 days later. Graphs represent GMT per time point per group.

Response rates to CS6, LT, and 0148 LPS following challenge are shown in Table 3. Anti-CS6 serologic response rates were comparably low (IgA: 12.8%; IgG: 17.0%) with no significant differences across the study groups (all p>0.05) or over time (Fig 4A and 4B). A total of 32.6% and 23.9% of naïve subjects demonstrated anti-CS6 ALS (antibody in lymphocyte supernatant) response by IgA and IgG, respectively. ALS or serum anti-LT responses in naïve subjects ranged from 0% to 57% depending on study group with no differences over time across the dose and fasting groups (Fig 4C and 4D). Additionally, there were no significant differences in anti-LT responses between naïve subjects (first and second cohorts) or veterans (second cohort) across any of the immune parameters (Fig 4C and 4D). The most prevalent immune response was to 0148 LPS, which was seen in the majority of subjects. Specifically, an anti-LPS serum IgA response was observed in 85.1% of all naïve subjects with titers peaking on day 7 post-challenge (Fig 5B). A similar anti-LPS IgA ALS response rate (89.4%) was observed among naïve subjects. In Cohort 2, serum IgA and ALS IgG responses to LPS were more frequent in naïve subjects (both 94.7%) than rechallenged veteran subjects (55.6% and 30.0%, respectively) despite comparable baseline titers (both p = 0.04) (Fig 5A).

**Table 3:** Number and percentage of subjects with a response to CS6, LT, and LPS as assessed by serologic and ALS titers.

	Study Group	Serology						ALS					
		IgG responders/total (%)			IgA responders/total (%)			IgG responders/total (%)			IgA responders/total (%)		
		CS6	LT	LPS	CS6	LT	LPS	CS6	LT	LPS	CS6	LT	LPS
**Cohort 1**	10^8^ cfu ON^a^	1/7 (14.3)	2/7 (28.6)	2/7 (28.6)	1/7 (14.3)	1/7 (14.3)	5/7 (71.4)	2/7 (28.6)	1/7 (14.3)	3/7 (42.9)	3/7 (42.9)	1/7 (14.3)	5/7 (71.4)
	10^9^ cfu 90 min	3/7 (42.9)	1/7 (14.3)	2/7 (28.6)	1/7 (14.3)	0/7 (0.0)	6/7 (85.7)	2/7 (28.6)	2/7 (28.6)	3/7 (42.9)	4/7 (57.1)	0/7 (0.0)	7/7 (100.0)
	10^9^ cfu ON	0/7 (0.0)	3/7 (42.9)	1/7 (14.3)	2/7 (28.6)	2/7 (28.6)	4/7 (57.1)	0/7 (0.0)	4/7 (57.1)	1/7 (14.3)	0/7 (0.0)	1/7 (14.3)	5/7 (71.4)
	10^10^ cfu 90 min	0/7 (0.0)	4/7 (57.1)	4/7 (57.1)	0/7 (0.0)	1/7 (14.3)	7/7 (100.0)	1/7 (14.3)	2/7 (28.6)	5/7 (71.4)	2/7 (28.6)	1/7 (14.3)	6/7 (85.7)
	Volunteers with MSD selected for cohort 2^b^	2/11 (18.2)	5/11 (45.5)	5/11 (45.5)	2/11 (18.2)	3/11 (27.3)	10/11 (90.9)	1/11 (9.1)	5/11 (45.5)	5/11 (45.5)	2/11 (18.2)	2/11 (18.2)	10/11 (90.9)
**Cohort 2**10^9^ cfu ON	Naïve subjects	4/19 (21.1)	10/19 (52.6)	7/19 (36.8)	2/19 (10.5)	3/19 (15.8)	18/19 (94.7)	6/18 (33.3)^e^	9/18 (50.0)^e^	18/19 (94.7)	6/18 (33.3)^e^	8/19 (42.1)	19/19 (100.0)
	Veteran subjects^c^	3/11 (27.3)	6/11 (55.6)	4/11 (36.4)	1/11 (9.1)	4/11 (36.4)	6/11 (55.6)	5/10 (50.0)^f^	8/10 (80.0)^f^	3/10 (30.0)^f^	6/10 (60.0)^f^	4/10 (40.0)^f^	8/10 (80.0)^f^
**All naïve subjects**^d^		8/47 (17.0)	20/47 (42.6)	16/47 (34.0)	6/47 (12.8)	7/47 (14.9)	40/47 (85.1)	11/46 (23.9)	18/46 (39.1)	30/47 (63.8)	15/46 (32.6)	11/47 (23.4)	42/47 (89.4)

a—overnight fasting.

b—eleven subjects selected from the four groups in cohort 1 who developed moderate-severe diarrhea (MSD).

c—subjects who developed MSD after challenge in cohort 1.

d—cumulative responses of all naïve subjects challenged in cohort 1 and 2 regardless of the dose.

e—samples from 18 of 19 subjects were available.

f—samples from 10 of 11 subjects were available.

*Response defined as ≥4 fold-rise in titers over baseline.

## Discussion

Our initial cohort with four dosing and fasting groups indicated that a 1 log reduction in the B7A inoculum dose induced a comparable disease rate if preceded by an overnight fast when compared to the more commonly utilized 90-minute fast. Despite equivalent attack rates (71.4%), the volunteers with the 10^9^ cfu dose and overnight fast had a higher mean ETEC disease severity score, greater frequency (median of 8 vs. 5), and 24-hour stool output volume (median of 1055 g vs. 607 g) than those who received 10^10^ with the 90-minute fast. The attack rate was lower (57.9%) in the confirmation cohort, suggesting that the lower dose with overnight fast may not yield consistent or sufficiently high attack rates for subsequent vaccination/challenge trials. This is in contrast to what was seen with the model refinement of the CFA/I-producing ETEC strain, H10407, where the inoculum dose was lowered by 2 logs with a longer fasting time and still produced consistent attack rates [12]. Also, in contrast with the H10407 study as well as a study by Levine et al with B7A [12, 15], we did not observe homologous protection upon B7A rechallenge; however, there are notable differences in the study designs. Levine et al gave an approximate 10^8^ cfu dose of B7A with 3 g of sodium bicarbonate after a 90-minute fast and 13% (1/8) of previously exposed subjects developed diarrhea compared to 58% (7/12) of naïve subjects [15]. Another major difference between studies was the time of antibiotic treatment after the initial challenge, day 8 by Levine et al compared with a median of 55.1 hours (IQR: 26.5, 107.9 hours) for the volunteers who went on to be rechallenged in our study. It may be that earlier antibiotic treatment blunted a sufficiently protective immune response or that the clinical illness from prolonged fasting and the higher inoculum dose overwhelmed any acquired immunity that was conferred by the initial challenge. An assessment of the subjects between the studies did not identify any clear differences between the immune responses. Our results are somewhat consistent with a 2007 study by Qadri et al [22] where in Bangladeshi birth cohort repeated diarrheal episodes were observed in children with CS6-expressing ETEC, potentially indicating insufficient protection following initial exposure. However, there are significant differences in the study populations and the setting of the exposure that confounds direct comparisons.

The greater immunogenicity of CFA/1 compared with CS6 may also have protected the volunteers in the H10407 rechallenge [12] from the illness seen on rechallenge with B7A, as the former volunteers were protected despite the similar timing of antibiotic treatment with the latter. Aspects of the clinical illness seen with B7A are distinctly different than with H10407. As previously described [10], symptom onset is rapid among B7A recipients, with a median time to illness as short as 11.4 hours in the 10^9^ cfu with 90-minute fast group, and with three of the volunteers with illness onset ≤5 hours. This was consistent with what was seen in other challenge studies with B7A [14, 15]. In addition, the volume and frequency of diarrhea is less than that seen with challenge with H10407, as previously described [10]. Despite these differences, shedding levels two days post-inoculation was associated with challenge dose and with clinical symptoms. Given the short incubation period, we tested the inoculum for the presence of pre-formed toxin using previously described methods [17, 18, 23] and only negligible quantities of ST were identified making it unlikely that preformed toxin contributed to the short incubation period observed with B7A.

In this study, we observed variable immune responses that did not consistently increase with higher dose or fasting time. Although the B7A strain has been used in many volunteer studies, this is the first study to comprehensively evaluate serum antibody responses and ALS response rates induced by this strain in naïve and rechallenged volunteers. Our serologic and ALS anti-CS6 immunologic assays reveal relatively low response rates. These response rates are comparable to rates observed with prior B7A challenge studies as well as other ETEC challenges [14]. Similarly, in natural ETEC infections of adult travelers, serologic response rates and maximum titers were low [24]. This is in contrast to robust serologic IgA and IgG titers observed among natural infections of patients in an adult endemic population [25]. In our study, in general more subjects demonstrated an immune response to B7A antigens as assayed by ALS as compared to serology. A similar observation has been made in a recent study of an inactivated whole cell ETEC vaccine, including CS6, when administered to Swedish adults [26]. In this study we observed the highest frequency of response was directed against 0148 LPS. As the ALS assay reflects the transient antigen-specific plasmablasts in the circulation during acute infection, this parameter may be more sensitive for evaluating the local intestinal immune responses, as seen by others [27].

In summary, we attempted to identify an optimal dosing and fasting regimen for the CS6-expressing ETEC strain B7A to maximize attack rates while minimizing the inoculum dose necessary to achieve a high and reproducible attack rate. We found that increasing the fasting time was not sufficient to allow for a decrease in the inoculum dose while maintaining a consistent attack rate; however, the rate of non-diarrheal symptoms, as demonstrated by the mean ETEC disease severity scores, appeared to increase with challenge dose for a given fasting regimen. The lack of homologous protection upon rechallenge observed in this study potentially indicates that a dose of 10^9^ cfu following an overnight fast may overwhelm vaccine-induced immunity. Alternatively, the short time to antibiotic treatment (five days or less) following challenge may not allow all subjects to develop a robust, protective immunity. The immune response to CS6 was variable and, generally poor and lower than previously described; however, variability in threshold, cut-offs, assay methodology and small sample size may account for this difference. Moving forward with challenge studies utilizing this strain, the 10^10^ cfu higher dose is likely necessary to ensure a consistent and predictable attack rate, but may underestimate protective immunity generated by a vaccine against naturally occurring infection.

## Supporting information

S1 File (XLSX)

S2 File (DOCX)

S3 File (DOC)

S4 File (PDF)
