# Supplementary material for: Refinement of the CS6-expressing enterotoxigenic Escherichia coli strain B7A human challenge model: A randomized trial
Source: PLoS One. 2020 Dec 2;15(12):e0239888. doi: 10.1371/journal.pone.0239888 (PMC7710093; doi:10.1371/journal.pone.0239888)
Supplement: S2 File — (DOCX) [file pone.0239888.s002.docx]

**Refinement of the human challenge model for B7A, An Enterotoxigenic *Escherichia coli* Challenge Strain Expressing CS6: a randomized trial**

**SUPPLEMENTARY METHODS**

**Eligibility Criteria**

Inclusion Criteria

- 1. Male or female between 18 and 50 years of age, inclusive.
  2. General good health, without clinically significant medical history, physical examination findings or clinical laboratory abnormalities per clinical judgment of the PI
  3. Completion of a training session and demonstration of comprehension of the protocol procedures and knowledge of ETEC-associated illness by passing a written examination (passing grade ≥ 70%)
  4. Willing to participate after informed consent obtained.
  5. Availability for the study duration, including all planned follow-up visits.
  6. Negative pregnancy test with understanding (through informed consent process) to not become pregnant during the study or within three months following last scheduled study visit. Females of childbearing potential must agree to use an efficacious hormonal or barrier method of birth control during the study. Abstinence is acceptable. Female subjects unable to bear children must have this documented (e.g. tubal ligation or hysterectomy) or must have negative pregnancy tests. Effective methods of avoiding pregnancy (including oral, topical or implanted contraceptives, IUD, female condom, diaphragm with spermicide, cervical cap, abstinence, use of a condom by the sexual partner, or sterile sexual partner) prior to dosing of the ETEC challenge strain.

Exclusion Criteria

*General health criteria*

1. Presence of a significant medical condition (e.g., psychiatric conditions; gastrointestinal disease, such as peptic ulcer, symptoms or evidence of active gastritis/dyspepsia, inflammatory bowel disease, irritable bowel syndrome (as defined by the Rome III criteria or medical diagnosis); alcohol or illicit drug abuse/dependency) which in the opinion of the investigator precludes participation in the study. Some medical conditions which are adequately treated and stable would not preclude entry into the study. These conditions might include stable asthma controlled with inhalers or mild hypertension stably controlled with a single agent.
2. Significant abnormalities in screening hematology, or serum chemistry as determined by PI or PI in consultation with the research monitor and sponsor.
3. Evidence of confirmed infection with HIV, Hepatitis B, or Hepatitis C.
4. Evidence of IgA deficiency (serum IgA < 7 mg/dL or below the limit of detection of assay).
5. Evidence of current excessive alcohol consumption or drug dependence (a targeted drug screen may be used to evaluate at the clinician’s discretion).
6. Evidence of impaired immune function.
7. Recent vaccination or receipt of an investigational product (within 30 days before receipt of challenge).
8. Any other criteria which, in the investigator’s opinion, would compromise the ability of the subject to participate in the study, the safety of the study, or the results of the study

*Research Related Exclusions Applicable to Challenge Participation*

1. History of microbiologically confirmed ETEC or cholera infection in last 3 years.
2. Occupation involving handling of ETEC or *Vibrio cholerae* currently or in the past 3 years.
3. Symptoms consistent with Travelers’ Diarrhea concurrent with travel to countries where ETEC infection is endemic (most of the developing world) within 3 years prior to dosing, OR planned travel to endemic countries during the length of the study.
4. Vaccination for or ingestion of ETEC, cholera, or *E. coli* heat labile toxin within 3 years prior to dosing.
5. Any prior experimental infection with ETEC strain B7A

*Study-specific Exclusion Criteria (potential increased risk or complicating outcome ascertainment)*

1. Abnormal stool pattern (fewer than 3 per week or more than 3 per day).
2. Regular use of laxatives, antacids, or other agents to lower stomach acidity.
3. Use of any medication known to affect the immune function (eg, systemic corticosteroids and others) within 30 days preceding the administration of challenge or planned use during the active study period.
4. Known allergy to two of the following antibiotics: ciprofloxacin, trimethoprim-sulfamethoxazole, and amoxicillin.

*Eligibility for proceeding to the second challenge after completing the first challenge*

1. Must continue to meet inclusion criteria above (including repeat of some safety labs)
2. Must not meet any of the exclusion criteria 1-8 and 14-17 listed above (prior ETEC exposure no longer exclusionary due to prior challenge)
3. Must have met the primary endpoint of moderate-severe diarrhea
4. All study subjects with serious adverse events from the primary challenge will be excluded from a repeat (second) challenge.

**Randomization Procedure**

For Cohort 1, volunteers were randomized 1:1:1:1 in block sizes of 4 to ensure comparable group sizes. The randomization code was performed using the PROC PLAN procedure in SAS v.9.2. (Cary, NC) at the Naval Medical Research Center (NMRC). Once run, the SAS Log, Code and Output was signed by the person performing the randomization. Subjects were assigned ID numbers, prior to admission, the ID numbers of 28 eligible subjects were provided to the NMRC coordinator, who randomized the subjects, and returned the randomization list to the Investigational Pharmacy and the CIR coordinator. Subjects in each treatment group were provided different color arm bands corresponding to the group. Their fasting time and the product they ingested depended on the study ID and the group that to which they were randomized.
