# Supplementary material for: Refinement of the CS6-expressing enterotoxigenic Escherichia coli strain B7A human challenge model: A randomized trial
Source: PLoS One. 2020 Dec 2;15(12):e0239888. doi: 10.1371/journal.pone.0239888 (PMC7710093; doi:10.1371/journal.pone.0239888)
Supplement: S4 File — (PDF) [file pone.0239888.s004.pdf]

Human challenge model refinement for B7A, An Enterotoxigenic *Escherichia coli* (ETEC) Challenge Strain that Expresses CS6

Sponsor	A. Louis Bourgeois, Ph.D., M.P.H.<br>Center for Immunization Research<br>Department of International Health<br>Johns Hopkins Bloomberg School of Public Health<br>624 N. Broadway, HH, Rm 205<br>Baltimore, MD 20215
Funding Agency	Jointly funded by: Joint Warfare Medical Research Program, Department of Defense<br>PATH-VAC
Principal Investigator(s)	Kawsar R. Talaat, MD<br>Department of International Health<br>Johns Hopkins University<br>Bloomberg School of Public Health<br>624 North Broadway, Rm. 249<br>Baltimore, MD, 21205<br>Telephone: 410-502-9627<br>Fax: 410-502-6898<br>Email: ktalaat@jhu.edu
Research Monitor	Jane L. Halpern, MD, MPH, DrPH<br>Department of International Health<br>Johns Hopkins Bloomberg School of Public Health<br>1219 Roundhill Road<br>Baltimore, MD 21218<br>Tel. 410-366-4823<br>Email: jhjp@comcast.net
NMRC Lead Investigator	Christopher Duplessis, MD, MPH, MS<br>Enteric Diseases Department (EDD)<br>Naval Medical Research Center (NMRC)<br>503 Robert Grant Avenue<br>Silver Spring, MD 20910<br>Telephone: 301-319-7312<br>Fax: 301-319-7679<br>E-mail: christopher.a.duplessis.mil@mail.mil
Subinvestigators	Ramiro L. Gutierrez, MD, MPH<br>EDD, NMRC<br>503 Robert Grant Avenue<br>Silver Spring, MD 20910<br>Telephone: 301-319-3193<br>E-mail: ramiro.l.gutierrez.mil@mail.mil<br><br>Mark S. Riddle, MD, DrPH

EDD, NMRC
Telephone: 301-319-7686
Fax: 301-319-7679
E-mail: mark.s.riddle10.mil@mail.mil

Chad Porter, PhD, MPH
EDD, NMRC
Telephone: 301-319-7505
Fax: 301-319-7679
E-mail: chad.k.porter2.civ@mail.mil

Milton Maciel, PhD, PharmD
EDD, NMRC
Telephone:
Fax: 301-319-7679
E-mail: Milton.M.Maciel.ctr@mail.mil

Clinical Trial Sites	Center for Immunization Research (CIR) Isolation Unit<br>301 Building<br>301 Mason Lord Drive Suite 4300<br>Baltimore, MD 21224<br><br>CIR Outpatient Clinic<br>624 N. Broadway, Hampton House Rm. 117<br>Baltimore, MD 21205
Site Investigational Product Accountability	Johns Hopkins Medicine<br>Department of Pathology, Division of Microbiology Meyer B-130<br>600 N. Wolfe Street<br>Baltimore, MD 21287-7070<br><br>Johns Hopkins Bayview Medical Center<br>Department of Pharmacy Research<br>4940 Eastern Avenue, AA bldg., Rm 149<br>Baltimore, MD 21224<br>Phone: 410-502-9627
Clinical laboratories	Quest Diagnostics, Inc.<br>Sulphur Spring Rd,<br>Baltimore, MD 21227<br><br>Johns Hopkins Hospital<br>600 North Wolfe Street<br>Baltimore, MD 21287<br><br>Johns Hopkins Bayview<br>4940 Eastern Ave.<br>Baltimore, MD, 21224

Research laboratories	Subhra Chakraborty, PhD, MS, MPH<br>CIR Enteric Research Laboratory (JHSPH)<br>615 N Wolfe Street / W5616 Baltimore, MD 21205<br>Telephone: 410-502-2510<br>Email: schakr11@jhu.edu<br><br>Milton Maciel, PhD, PharmD<br>ETEC immunology laboratory (EDD, NMRC)
Data Management	The EMMES Corporation<br>401 N Washington Street, NW Suite 700<br>Rockville, MD 20850<br>Telephone: 301-251-1161 (Administrative Coordinator)
Institutional Review Boards	Western Institutional Review Board (IRB of record)<br>1019 39th Ave. SE, Puyallup, WA 98374<br>Fax: (360) 252-2498<br>Email: clientservices@wirb.com<br><br>Naval Medical Research Center Institutional Review Board<br>Research Services Directorate<br>Office of Research Administration<br>Code 025, Building 500, Room 004<br>Silver Spring, MD 20910<br>Telephone: 301-319-7276

Investigator's Agreement

Human challenge model refinement for B7A, An Enterotoxigenic *Escherichia coli* (ETEC) Challenge Strain that Expresses CS6

"I have read this protocol and agree to conduct the study as outlined herein in accordance with International Conference on Harmonization Good Clinical Practice Guideline and FDA and DoD Regulations."

Kawsar Talaat
Kawsar R. Talaat, MD

19 Jan 2016
Date

Principal Investigator

1. Protocol Synopsis

PROTOCOL TITLE	Human challenge model refinement for B7A, An Enterotoxigenic *Escherichia coli* (ETEC) Challenge Strain that Expresses CS6
IND NUMBER	To be determined
CHALLENGE STRAINS	ETEC strain B7A (O148:H28 $CS6^{+}$ $LT^{+}ST^{+}$) (Lot 0481)
SPONSOR	A. Louis Bourgeois, Ph.D., M.P.H.
MANUFACTURER	Pilot Bioproduction Facility, Walter Reed Army Institute of Research, Silver Spring, MD
PRINCIPAL INVESTIGATOR	Kawsar R. Talaat, MD
STUDY SITES	Center for Immunization Research (CIR) Isolation Unit<br>301 Building<br>301 Mason Lord Drive Suite 4300<br>Baltimore, MD 21224<br><br>CIR Outpatient Clinic<br>624 N. Broadway, Hampton House Rm. 117<br>Baltimore, MD 21205
LABORATORIES	Quest Diagnostics Incorporated, Baltimore, MD 21227<br>Johns Hopkins Hospital, Baltimore, MD 21287<br>Johns Hopkins University School of Public Heath, Baltimore, MD, 21205<br>Naval Medical Research Center, Silver Spring, MD 20910
STUDY OBJECTIVES	Primary<br>1. Evaluate the safety of the challenge model.<br>2. Identify a dose and fasting regimen that induces moderate-severe diarrhea in at least 70% of naïve subjects without causing high output diarrhea (determined by stool output volumes or signs and symptoms associated with hypovolemia)<br>3. Assess protection upon repeat exposure to homologous ETEC strain (applying previously determined orally administered challenge inoculum)<br><br>Secondary<br>1. Measure mucosal and systemic immune responses to experimental infection<br>2. Obtain and archive samples for future proteomics, microbiome and/or systems biology efforts<br>3. Compare B7A shedding level (at Day 2 and Day 4) in subjects infected for the first time and in those re-challenged with a homologous ETEC strain.<br><br>Exploratory<br>1. Immunology and systems biology analyses to include (but not limited to) transcriptomics, proteomics, phosphoproteomics, and immune profiling<br>2. Evaluate the cognitive impact of acute diarrhea using a wrist-worn actigraph and psychomotor vigilance testing<br>3. Evaluate the impact of both the B7A ETEC challenge and antibiotic exposure on short term changes in host microbiota.

<table>
<tr><td></td><td>4. Explore the impact of microbiota on disease susceptibility
5. Evaluate the impact of the B7A ETEC challenge on short term changes in intestinal inflammation/repair, epithelial barrier function, motility, and systemic immune dysregulation.</td></tr>
<tr><td>STUDY DESIGN</td><td>This will be a dose-finding study in which B7A ETEC will be administered simultaneously at three dose levels as outlined in the table below. A second, confirmatory cohort will include up to 15 additional subjects with the potential to re-challenge previously exposed subjects. Following a pre-specified fast, subjects will drink 120 ml of sodium bicarbonate just prior to ingesting 30 ml of sodium bicarbonate containing the ETEC inocula. Subjects will be assessed daily for adverse events and all stools will be collected to assess for the primary endpoint of moderate (4-5 loose stools in 24 hours or 401-800 g of loose stools in 24 hours) to severe (> 6 loose stools in 24 hours or >800g of loose stools in 24 hours) diarrhea post-inoculation. Any subject passing a grade 3-5 stool will be encouraged to start drinking oral rehydration solution (ORS) (an oral glucose/electrolyte solution to prevent dehydration) at a rate equal to their stool output. IV rehydration will be provided if pre-specified criteria are met. All subjects will be treated with ciprofloxacin (500 mg by mouth twice daily for three days) five days after infection unless early treatment criteria are met. Subjects will be discharged from the inpatient facility when clinical symptoms are resolved or resolving AND two consecutive stool cultures are negative for ETEC.

Study design overview
<table>
<tr><th>Cohort</th><th>N</th><th>Dose (cfu)</th><th>Pre-dose fasting time</th></tr>
<tr><td rowspan="4">1</td><td>7</td><td>10^{8}</td><td>Overnight</td></tr>
<tr><td>7</td><td>10^{9}</td><td>90 minutes</td></tr>
<tr><td>7</td><td>10^{9}</td><td>Overnight</td></tr>
<tr><td>7</td><td>10^{10}</td><td>90 minutes</td></tr>
<tr><td rowspan="2">2</td><td>15</td><td colspan="2">Naïve subjects (dose and fasting regimen determined from Cohort 1)</td></tr>
<tr><td>15</td><td colspan="2">Veteran subjects from Cohort 1</td></tr>
</table></td></tr>
<tr><td>PRIMARY ENDPOINT</td><td>Moderate or severe diarrhea (as defined below) post-inoculation:

• Moderate diarrhea: 4 to 5 loose/liquid stools or 401-800 g of loose/liquid stool in any 24-hour period
• Severe diarrhea: ≥ 6 loose/liquid stools or > 800 g of loose/liquid stool in any 24-hour period</td></tr>
<tr><td>STUDY DURATION</td><td>After screening and selection of subjects, eligible individuals will be admitted to the inpatient facility on day -1. The inpatient period will last up to 10 days during which time subjects will be challenged with ETEC strain B7A, followed for clinical symptoms, and treated with antibiotics. Outpatient follow-up after challenge will occur about 28 days after challenge with a telephone follow-up for general health at 6 months. The re-challenge will occur between 1 and 8 months following the initial challenge. Immunologic testing will require approximately 3 months following the last clinic visit (study day 28). The entire study, considering screening, follow-up, immunologic testing, multiple inpatient challenge periods, analysis and reporting will last approximately 2.5-3 years.</td></tr>
<tr><td></td><td>Subjects will be recruited from the Baltimore and the surrounding area via</td></tr>
</table>

ELIGIBILITY CRITERIA	advertisements and word of mouth and screened at the Center for Immunization Research (CIR). Up to eight alternates per cohort will be recruited to replace anyone who does not report or is unable to participate at time of inpatient unit admission.<br><br>Inclusion Criteria:<br>1. Male or female between 18 and 50 years of age, inclusive.<br>2. General good health, without clinically significant medical history, physical examination findings or clinical laboratory abnormalities per clinical judgment of the PI.<br>3. Completion of a training session and demonstration of comprehension of the protocol procedures and knowledge of ETEC-associated illness by passing a written examination (passing grade ≥ 70%)<br>4. Willingness to participate after informed consent obtained.<br>5. Availability for the study duration, including all planned follow-up visits.<br>6. Negative pregnancy test with understanding (through informed consent process) to not become pregnant during the study or within three months following last scheduled study visit. Females of childbearing potential must agree to use an efficacious hormonal or barrier method of birth control during the study. Abstinence is acceptable. Female subjects unable to bear children must have this documented (e.g. tubal ligation or hysterectomy) or must have negative pregnancy tests. Effective methods of avoiding pregnancy (including oral or implanted contraceptives, IUD, female condom, diaphragm with spermicide, cervical cap, abstinence, use of a condom by the sexual partner, or sterile sexual partner) should be used prior to dosing of the ETEC challenge strain.<br><br>Exclusion Criteria:<br>General health criteria<br>1. Presence of a significant medical condition (e.g., psychiatric conditions; gastrointestinal disease, such as peptic ulcer, symptoms or evidence of active gastritis/dyspepsia, inflammatory bowel disease, irritable bowel syndrome (as defined by the Rome III criteria or medical diagnosis); alcohol or illicit drug abuse/dependency) which in the opinion of the investigator precludes participation in the study. Some medical conditions which are adequately treated and stable would not preclude entry into the study. These conditions might include stable asthma controlled with inhalers or mild hypertension stably controlled with a single agent.<br>2. Significant abnormalities in screening hematology or serum chemistry as determined by PI or PI in consultation with the research monitor and sponsor.<br>3. Evidence of confirmed infection with HIV, Hepatitis B, or

Hepatitis C.

4. Evidence of IgA deficiency (serum IgA < 7 mg/dL or below the limit of detection of assay).
5. Evidence of current excessive alcohol consumption or drug dependence (a targeted drug screen may be used to evaluate at the clinician's discretion).
6. Evidence of impaired immune function.
7. Recent vaccination or receipt of an investigational product (within 30 days before receipt of challenge).
8. Any other criteria which, in the investigator's opinion, would compromise the ability of the subject to participate in the study, the safety of the study, or the results of the study

Research Related Exclusions Applicable to Challenge

9. History of microbiologically confirmed ETEC or cholera infection in last 3 years.
10. Occupation involving handling of ETEC or *Vibrio cholerae* currently, or in the past 3 years.
11. Symptoms consistent with Travelers' Diarrhea concurrent with travel to countries where ETEC infection is endemic (most of the developing world) within 3 years prior to dosing, OR planned travel to endemic countries during the length of the study.
12. Vaccination for or ingestion of ETEC, cholera, or E coli heat labile toxin within 3 years prior to dosing.
13. Any prior experimental infection with ETEC strain B7A.

Study-specific Exclusion Criteria (potential increased risk or complicating outcome ascertainment)

14. Abnormal stool pattern (fewer than 3 per week or more than 3 per day).
15. Regular use of laxatives, antacids, or other agents to lower stomach acidity.
16. Use of any medication known to affect the immune function (eg, systemic corticosteroids and others) within 30 days preceding the administration of challenge or planned use during the active study period.
17. Known allergy to two of the following antibiotics: ciprofloxacin, trimethoprim-sulfamethoxazole, and amoxicillin.

Eligibility for proceeding to the second challenge after completing the first challenge

18. Must continue to meet inclusion criteria above
19. Must not meet any of the exclusion criteria 1-8 and 14-17 listed above (prior ETEC exposure no longer exclusionary due to prior challenge)
20. Must have met the primary endpoint of moderate-severe diarrhea
21. All study subjects with serious adverse events from the primary

	challenge will be excluded from a repeat (second) challenge.
METHODS	Subjects will be monitored for diarrhea and other signs/symptoms of enteric illness by daily medical checks, vital sign determinations, grading (loose and liquid stools are graded as 3-5) and weighing of all stools. Five days after challenge (or sooner if the subject meets early treatment criteria), subjects will be treated with ciprofloxacin (500 mg by mouth twice daily for three days), except in cases of known allergy or intolerance in which case a suitable alternative will be utilized. All subjects will be discharged from the inpatient unit when they are well and have had at least 2 consecutive stool cultures negative for the challenge strain. Follow-up visits for 4 weeks post-challenge will monitor safety and immunologic parameters.<br><br>A second inpatient period will be utilized to confirm the fasting and dosing regimen and to assess protection upon homologous re-challenge. This second challenge will occur between 1 and 8 months following the initial challenge.<br><br>After study completion, an adjudication board will be used to independently evaluate ETEC disease outcome data.
STUDY PROCEDURES	
TEST ARTICLE DOSING	Subjects will be admitted as inpatients and challenged with 10^8 to 10^{10} CFUs of ETEC strain B7A following a 90 minute (10^9or 10^{10}) or an overnight (10^8or 10^9) fast. Following the designated fast, subjects will drink 120 ml of sodium bicarbonate just prior to ingesting 30 ml of sodium bicarbonate containing the ETEC inoculum.
CLINICAL MONITORING	Daily medical assessments with adverse event determination, vital signs three times daily, examine and weigh all stools, stool culture work-up for challenge strain (up to three times daily), and safety laboratory tests (refer to Time and Events Schedule).<br><br>The primary clinical endpoint is moderate to severe diarrhea according to the following definitions post-inoculation:<br>• Severe diarrhea: ≥ 6 grade 3-5 stools or > 800 g of grade 3-5 stools in any 24 hour period<br>• Moderate diarrhea: 4-5 grade 3-5 stools in 24 hours or 401-800 g of grade 3-5 stools in any 24 hour period<br><br>Secondary clinical endpoints are chosen to support the primary endpoint and include the following:<br>• Maximum 24-hour stool output<br>• Percent of subjects with severe diarrhea<br>• Percent of subjects with diarrhea of any severity<br>• Total weight of grade 3-5 stools passed per subject over during the inpatient period<br>• Number of grade 3-5 stools per subject<br>• Percent of subjects with nausea, vomiting, anorexia, or abdominal pain/cramps rated as moderate to severe<br>• Mean time to onset of diarrhea<br>• Number of subjects with moderate to severe ‘ETEC illness’<br>• Number of cfu of the challenge strain per gram of stool 2 and 4 days post challenge

	• ETEC systemic and diarrhea severity score post-challenge<br>• Cognitive evaluation: Exploratory evaluation of the cognitive impact of acute diarrhea will be performed during the inpatient phase with the use of wrist worn Psychomotor Vigilance Testing (PVT) and actigraphy devices.
MICROBIOLOGY	**Standard**<br><br>Intestinal colonization by the challenge strain will be assessed by monitoring fecal shedding patterns by qualitative culture.<br><br>**Exploratory:**<br><br>Microbiota:<br>An assessment of changes in microbiota will be performed employing 16s ribosomal RNA sequencing of the microbial community present in the collected stool sample. Human gut microbiota is stable in a given host but highly variable amongst individuals. For this reason, stool from each individual subject will be collected prior to challenge (infection) in order to establish their specific baseline gut microbiota composition.<br><br>Gut microbiota changes of individual subjects will be evaluated following B7A ETEC challenge and following antibiotic exposure and compared to their "normal" gut microbiota composition (prior to infection). This survey will identify gut microbiota modification during the development of B7A ETEC enteritis and following antibiotic treatment.<br><br>Barrier Dysfunction & Dysbiosis: Fecal samples will assayed for fecal α1-antitrypsin, a marker of protein leakage into the intestinal tract. In addition, to measure changes in barrier function and response, serum will be collected to test for changes in immunodominant antigens of the microbiota that stimulate T cells and have been shown to be associated with inflammatory bowel diseases. Seroreactivity to these flagellins is found in multiple experimental models of colitis in mice. The protein based microarray includes 45 select antigens. Blood samples may be taken for a limulus assay.<br><br>Intestinal Inflammation & Repair: Serum samples will also be tested for changes in levels of leptin (and IL-8), and fecal samples will be obtained to evaluate REG1, calprotectin, neopterin, cytokines, and myeloperoxidase.<br><br>Additional culture-independent methods may be used to quantitate B7A shedding.
RESEARCH IMMUNOLOGY	Immunogenicity evaluation will be conducted primarily by analysis of serum anti-CS6 and anti-toxin parameters by ELISA and ALS. In addition, exploratory expanded immunological analyses will be conducted.<br><br>Systemic-immune response:<br>Serum samples will be assayed for IgG and IgA antibody titers against LT, using the GM1-ELISA and against O148, and CS6 using methods previously established. Previously established high-titer specimens will be included on each plate to track day-to-day inter-assay variation. For each antigen, pre- and

	post-challenge serum samples will be assayed side-by-side. The antibody titer assigned to each sample will represent the geometric mean of duplicate tests. Reciprocal endpoint titers < 50 will be assigned a value of 25 for computational purposes. Seroconversion will be defined as a > 4-fold increase in endpoint titer between pre-and post-challenge samples.<br><br>Secondary immune response outcomes:<br>Peripheral blood mononuclear cells (PBMCs) isolated from EDTA venous blood will be assayed for antigen specific IgG and IgA Antibody Lymphocyte Supernatants (ALS) responses representative of mucosal response.<br><br>Exploratory expanded immunological evaluation: Exploratory and expanded immunological assessments will be planned for this study for which samples of serum and cells may be collected and evaluated pending funding availability. Among these, serum and PBMC samples will be collected for transcriptomic, cytokine, proteomic, antigen microarrays, and other systems biology analyses to identify molecular signatures associated with ETEC infection. The cytokine analyses will encompass representation from multiple pathways including pro-and anti-inflammatory, and regulatory pathways.<br><br>Antigen specific memory B cell quantification may also be performed [purified PBMCs will be archived to determine the levels of B7A ETEC-specific memory B cells that are generated following challenge]. Stool collected from the subjects may be used to assess for B7A ETEC challenge antigen-specific fecal IgA responses. Saliva may be collected to assess the salivary IgA responses.<br><br>**Cognitive evaluation**: Exploratory evaluation of the cognitive impact of acute diarrhea will be performed during the inpatient phase with the use of wrist worn Psychomotor Vigilance Testing (PVT) and actigraphy devices.
CLINICAL MANAGEMENT	Fluid Management<br><br>Oral: Any subject passing a grade 3-5 stool will be encouraged to start drinking oral rehydration solution (ORS) (an oral glucose/electrolyte solution to prevent dehydration) or other oral rehydration fluid at a rate equal to their stool output.<br><br>Intravenous: During the inpatient period of the study, a subject may be administered IV fluids (clinician discretion) if they:<br>• experience abrupt onset of diarrhea, defined as passage of an initial loose/liquid stool of > 300g, or > 400 g of loose stool over 2 hours in conjunction with other symptoms, as determined by the PI or designee.<br>• become hypovolemic, defined as confirmed supine systolic BP < 90 mmHg and associated symptoms, or significant lightheadedness on standing, with a confirmed postural change in BP or pulse. Postural vital signs will be measured lying and 2 minutes after standing. A significant change is a decrease in systolic BP of > 20 mmHg, or diastolic BP of >10 mmHg or increase in pulse of > 30 beats/minute.<br>• are determined necessary by the study physician; eg, diarrhea with nausea/vomiting and subject is unable to keep up with output, or for other reason(s).

	Antibiotic Treatment<br>Antibiotic treatment after challenge will be administered according to criteria for early antibiotic treatment (described below) or 5 days after challenge if no diarrhea develops. Subjects will be treated with an antibiotic (ciprofloxacin (500 mg by mouth twice daily for 3 days), except in cases of known allergy or intolerance in which case a suitable alternative will be utilized. Alternative treatments are trimethoprim 160 mg / sulfamethoxazole 800 mg by mouth twice daily for three days, or amoxicillin (500 mg by mouth 3 times daily for 3 days). Subjects will be discharged from the inpatient facility when they are well and after they have had 2 consecutive stool cultures that are negative for the challenge strain (may be collected on the same day). Routine treatment will commence at about 120 hours (on the morning of day 5) post-challenge. If, because of illness, a subject is unable to take oral antibiotics, intravenous antibiotics may be given (IV ciprofloxacin at an appropriate dose based on weight and clinical status).<br><br>Treatment for vomiting may be needed. Subjects who are vomiting may be given ondansetron (Zofran) ODT or ondansetron IV for the management of vomiting.<br><br>Early antibiotic treatment after challenge will commence when any of the following criteria are identified and a study physician considers it to be warranted:<br>• Severe diarrhea (based on volume, 800 g in 24 hours)<br>• Stool output consistent with moderate diarrhea for 48 hours<br>• Mild or moderate diarrhea and 2 or more of the following symptoms: severe abdominal pain, severe abdominal cramps, severe nausea, severe headache, severe myalgias, any fever (≥ 38.0°C), or any vomiting.<br>• A study physician determines that early treatment is warranted for any other reason
DISCHARGE PROCEDURES	All subjects are scheduled for discharge from the inpatient ward approximately 8 days after receipt of the challenge inoculum. The day of discharge may be earlier if the subject qualifies for early antibiotic treatment. Subjects will be discharged from the inpatient phase of the study when clinical symptoms are resolved or resolving AND two consecutive stool cultures are negative for the challenge strain.
SAMPLE SIZE	The aim to down select an ETEC challenge strain dose with a ≥ 70% attack rate can be accomplished in a minimum of 7 subjects with a confidence interval of 29 - 96%. Increasing the number of subjects by 15 for the selected inoculum/fasting regimen (ie, Cohort 2) will provide greater confidence (50 - 89%) that the target 70% attack rate will be achieved in future applications of the challenge model.<br><br>A sample size of 15 subjects per arm in Cohort 2 provides an 80% power to detect a moderate-severe diarrhea risk difference of 55% presuming a minimum of a 70% attack rate in naïve subjects.
ANALYSIS	Demographic and clinical information will be collected. Adverse events will be summarized by challenge dose. The descriptive statistics presented for each

	system organ class (SOC) and preferred term will be the number of subjects with event (N), the percent of subjects exposed with event (%), and the number of events (E). All adverse events will be listed by subject number, dose, latest version of MedDRA system organ class, and MedDRA preferred term.<br><br>Clinical laboratory values and vital signs will be listed. All values outside normal range (at screening and at any follow-up visits) will be listed by subject number and flagging of clinical significant values.<br><br>Immunologic responses will be analyzed by comparing the number of responders per group, as well as the magnitude of the responses in the various groups. Student's t-tests or Wilcoxon tests will be used as appropriate.<br>All statistical tests will be interpreted in a two-tailed fashion using an alpha= 0.05.

2. Time and Events schedule

	Screening 1-2 visits	Challenge Phase (Inpatient)					f/u Visits and call		
**Study Event**	**-60**[a]	**-1**	**0**	**1-4**	**5**	**6-7**	**8**[b]	**28**	**180**[c]
Compliance Ranges	-60 to -1	--	--	--	--	--	±1	±3d	±1m
Study Briefing[a]	X								
Comprehension Assessment	X								
Informed Consent (study participation)	X								
Medical History and Physical Examination[d]	X								
Chemistry, Hematology, & Blood typing[e]	X								
Anti-HIV-1, Anti-HCV, HBsAG[f]	X								
Serum IgA	X								
Urine toxicology screen[g]	X								
Clinical Check Vitals (BP, HR, T)[h]	X	X	X	X	X	X	X	X	
Pregnancy test[i]	X	X						X	
Inpatient		X	X	X	X	X	X		
Challenge			X						
Stool weighing and grading[j]			X	X	X	X	X		
Stool bacteriology[k]		X	X	X	X	X	X		
Start antibiotic therapy[l]					X				
Saliva and Fecal Collection(Immunology)		X						X	
Planned discharge							X		
Clinical check [m, h, n]	X	X	X	X	X	X	X	X	
Serology[o]		X						X	
ALS[o]		X				X			
Exploratory Immunology[p]	X	X		X	X	X		X	
Microbiome Assays		X	X	X	X	X	X	X	
Cognitive Study (PVT/Actigraphy)[q]		X	X	X	X	X	X		
Functional Bowel Disorder Survey	X								X
Study completion								X	
Telephone Contact									X
Approximate blood volume (mL) by study day[r]	53	95	0	29	5	32	0	66	

Note: Subjects being rechallenged will repeat the above schedule with a targeted second challenge occurring between 1 and 8 months following the initial challenge. Repeat screening procedures at a minimum will include PE, CBC and serum chemistry. The maximum volume of blood to be collected over a two-month window will be no more than 500 mL. Rechallenged subjects will not be required to complete the Day 180 follow-up telephone call for their initial challenge.

[a] Visits are defined as following, see above for compliance windows:

- Study Day -60 (-60 to -1) is screening
- Day -1: Day prior to challenge
- Days 0-6 are the challenge inpatient period
- Day 7: Day of discharge or short interval follow-up after early discharge
- Day 28: 28 days after challenge
- Days 180 is a long term follow-up phone calls

[b] Criteria for discharge from the unit: Subjects can be discharged from the inpatient phase of the study when they have completed a minimum of 2 doses of abx, clinical symptoms are resolved or resolving and 2 consecutive stool cultures are negative for ETEC. Subjects will be required to complete their abx as

outpatients. It is expected that most subjects will be discharged on days seven or eight. If a subject does not fulfill criteria for discharge he/she may be required to stay on the unit until all criteria have been fulfilled.

[c] At six (6) months post-inoculation, phone calls will take place to inquire about the occurrence of any serious health events. Rechallenged subjects will not be required to complete the Day 180 follow-up telephone call for the initial challenge.

[d] Physical Examination (PE) will include: HEENT (Head; Ears; Eyes; Nose; Throat), skin, respiratory (lung), cardiovascular (heart), abdomen, neurological and musculoskeletal system. The BMI will be utilized by the PI as a relative measure of general health to be used on a case-by-case basis. PE will be done at screenings, and on admission. During the inpatient period a symptom focused PE will be completed.

[e] Hematology will include: Hemoglobin, White blood cell count (WBC) with differential, and platelets; additionally, ABO and RH blood typing will be done prior to challenge.

Serum Chemistry will include: ALT (SGPT), AST (SGOT), Blood urea nitrogen (BUN), creatinine, random glucose. Follow up sample to be taken if clinically significant abnormalities are seen. Hematology and serum chemistry will be performed at screening only unless needed for medical follow-up purposes during the course of the study. Clinically relevant laboratory abnormalities will be recorded as medical history if obtained before day 0.

Not clinically significant laboratory abnormalities not on toxicology table can be recorded on the MH if deemed necessary by the PI. If indicated, subjects may have additional blood draws taken for monitoring of serum electrolytes, blood cultures or for other safety reasons.

[f] Screening Serology will include HIV, HBsAg, HCV and total IgA (Serum IgA < 7 mg/dL or limit of detection of assay will be considered exclusionary).

[g] Urine Drug Screen will test for the presence of amphetamine, barbiturates, opiates, phencyclidine, cocaine, and benzodiazepine, methadone, and propoxyphene screening at the discretion of the study clinician.

[h] Vital Signs (VS) will include heart rate, blood pressure, and oral temperature. Vital signs are obtained from study subjects at protocol-directed time points throughout the study. There will be instances when a vital sign needs to be repeated. Standard practice will be to repeat the vital sign within approximately 20 minutes of the original reading. Only the vital sign that needs to be repeated will be repeated. Both the original and repeat measurements will be recorded in the study source documents. If, in the judgment of the PI or his designee, the repeat measurement is a more appropriate reflection of the subject's vital sign, the repeat measurement will be recorded in the CRF field for that measurement. The other vital signs will be recorded in their respective CRF fields, at the time they were obtained the repeated value may be entered as the value for the original time even though it may have been obtained several minutes later than the original vital signs.

The following VS are obtained and documented in the source documents:

- Sometime during the screening visit
- Sometime during the admission to challenge
- Before and after challenge
- At least 3 times daily during in-patient period
- At the day 7 visit
- At the day 28 visit

A grade 1 bradycardia, or other grade 1 abnormalities will not be considered to be exclusionary at screening or an AE for the study, unless judged to be clinically significant by the PI. Clinically relevant and concurrent medical conditions or surgical procedures will be recorded as medical history obtained prior to challenge and as an AE if obtained after challenge. This includes pre-existing lab abnormalities, VS abnormalities, and symptoms associated with menses (e.g. cramps, headaches, etc.) Not clinically significant abnormalities not on the toxicology table can be recorded on the MH if deemed necessary by the PI. These abnormalities should not be recorded as AEs.

Disregarding clinical relevance and clinically significant, the following VS will be captured in the electronic CRF:

- Before and after challenge
- At discharge
- At visit day 7

- At visit day 28

[i] Serum pregnancy tests will be performed at screening and the day of admission, prior to challenge and a urine test on the last scheduled follow-up visit. If the serum pregnancy test on the day of admission to challenge does not return a result in time, a urine pregnancy test will be performed before challenge (Day 0).

[j] Stool weighing and grading. During the inpatient period all stool samples are collected, weighed and graded. If a subject meets discharge criteria prior to day 7, no further stool samples will be collected.

[k] Stool sample for bacteriology will begin the day after challenge, or prior to institution of early antibiotic therapy (whichever is sooner). If a stool sample is not obtained before 1300 hours a rectal swab will be obtained (the swab will be collected about 1300 to allow for processing). Swabs will be used only to obtain stools to be processed for bacteriology. Stool samples will be collected for assays as specified in the laboratory study of event schedule and as per written SSPs. A subset of these samples, during high shedding points, will be reserved for the later validation and development of bacteriological assays for shedding of ETEC and other organisms. Additionally, stool samples will be obtained to assess for exploratory endpoints to include microbiome, PCR and transcriptomics.

[l] Six doses of ciprofloxacin (cipro) 500 mg BID, to start five days after challenge for three days, or suitable alternative trimethoprim-sulfamethoxazole (1 DS tablet twice daily for three days) or amoxicillin (500 mg three times daily for 3 days); unless subject meets criteria for early antibiotic treatment.

[m] After screening, subject continuing eligibility must be confirmed by reassessing relevant inclusion and exclusion criteria before challenge on either day of admission or challenge.

[n] Interim medical interview and AE collection will be conducted at each outpatient follow-up visit at the clinical center.

[o] Blood for immunology endpoints will be collected as specified in the laboratory study of event schedule and as per written SSPs. Total approximate predefined blood volumes can be found in the laboratory study event schedule.

[p] Exploratory Immunology. Systems biology--Expanded immunology assessments to include antigen-specific cellular assays, pathogen-specific proteomics, transcriptomics, and host proteomics to evaluate the immune response to challenge. Exploratory immunological evaluations are outlined in the immunology lab study and events schedule. Samples will be obtained and reserved pending funding availability.

[q] Exploratory Cognitive Assessment will be performed on all individuals during the inpatient phase (thrice daily). Wrist-based PVT evaluation will be performed throughout the inpatient phase.

[r] Approximate blood volumes: Day 1 we will collect 24 mL for exploratory immunology. Day 2 we will not collect blood. Day 3 we will collect 5 mL for exploratory immunology. Day 4 will not have blood collection. Day 5 we will collect 5 mL for exploratory immunology. Day 6 will not have blood collection. Day 7: we will collect 32ml

3. Table of Contents

1. PROTOCOL SYNOPSIS 5
2. TIME AND EVENTS SCHEDULE 14
3. TABLE OF CONTENTS 17
4. LIST OF ABBREVIATIONS AND DEFINITIONS OF TERMS 20
5. INTRODUCTION 22
5.1. Clinical significance 22
5.5 Evidence for anti-CF Immunity 24
5.6 History of the ETEC Human Challenge Model 25
6. STUDY OBJECTIVES 26
6.1. Primary Objectives and Endpoints 26
6.2. Secondary Objectives and Endpoints 27
Secondary endpoint measures: 27
6.3. Exploratory Objectives 27
7. TRIAL DESIGN 27
7.1. Study Design 27
7.2. Recruitment 28
7.3. Screening 29
7.3.1. Inclusion criteria 30
7.3.2. Exclusion criteria 31
7.4. Day -1 32
7.5. Challenge 32
7.6. Inpatient monitoring 32
7.7. Outcome verification and rechallenge 35
7.8. Concomitant medication 35
7.9. Handling of study samples 35
7.10. Outcome measures 35
7.10.1. Clinical 35
7.10.2. Immunological 36
7.10.3. Exploratory 37
7.10.4. Outcome Adjudication Committee 38
8. INVESTIGATIONAL PRODUCT 38
8.1. Label 38
8.2. Product characterization 38
8.3. Product preparation 39
8.4. Storage 39
9. SUBJECT WITHDRAWAL 39
9.1. When and how to withdraw subjects 40
9.2. Replacement of subjects 40
9.3. Follow-up for withdrawn subjects 40
10. SAFETY ASSESSMENT 40
10.1. Vital signs 41
10.2. Physical examination 42
10.3. Laboratory assessments 42
10.4. IND safety reporting 44

10.4.1. Adverse event or suspected adverse reaction ... 44
10.4.2. Solicited and anticipated adverse events ... 44
10.4.3. Serious adverse event or serious suspected adverse reaction ... 45
10.4.4. Unexpected adverse event or unexpected suspected adverse reaction ... 45
10.4.5. Other adverse event ... 46
10.5. Relationship to investigational product ... 46
10.6. Recording of adverse events ... 46
10.6.1. Methods / Timing for assessing, recording and analyzing safety endpoints ... 46
10.6.2. Duration of follow-up of subjects after adverse events ... 47
10.6.3. Safety assessment ... 47
10.7. Reporting adverse events ... 49
10.7.1. Reporting serious and unexpected adverse events ... 49
10.7.1.1. Reporting to the sponsor ... 49
10.7.1.2. Reporting to the IRB ... 51
10.7.2. Immediately reportable events ... 52
10.7.2.1. Pregnancy ... 52
10.7.2.2. AE-related withdrawal of consent ... 52
10.7.2.3. Pending inspections/Issuance of reports ... 52
10.7.3. IND reporting ... 52
10.7.3.1. Annual reports ... 52
10.7.3.2. Final clinical study report ... 53
10.8. Safety Criteria for Stopping Doses ... 53
10.9. Study Termination Criteria ... 53
11. STATISTICAL CONSIDERATIONS ... 53
11.1. Introduction ... 53
11.2. Sample size consideration ... 54
11.3. Analysis ... 54
12. DATA HANDLING AND RECORDKEEPING ... 54
13. RECORD AND SPECIMEN ARCHIVAL ... 55
14. OBLIGATIONS AND ROLES OF SPONSOR, INVESTIGATOR AND STUDY PERSONNEL ... 55
15. QUALITY CONTROL AND ASSURANCE ... 56
15.1. QA/QC monitoring ... 56
15.2. Protocol deviation management ... 56
15.3. Monitoring ... 56
16. HUMAN SUBJECTS PROTECTIONS CONSIDERATIONS ... 57
16.1. Risks / Benefit ... 57
16.1.1. Risks ... 57
16.1.2. Risk mitigation strategies ... 58
16.1.3. Benefits ... 59
16.2. Subject compensation ... 59
16.3. Research related injury ... 60
16.4. Compensation for investigators ... 62
16.5. Fair and equitable selection of subjects ... 62
16.6. Informed consent ... 62

17. PRIVACY AND CONFIDENTIALITY 63
17.1. Storage of data and samples 63
17.2. Provisions protecting privacy and confidentiality 63
17.3. Safeguards for vulnerable subjects 63
18. PROTOCOL REVIEW PROCESS 63
11 PUBLICATION POLICY 64
12 LIST OF REFERENCES 65

4. List of Abbreviations and Definitions of Terms

Abbreviation	Explanation
abx	antibiotic
AE	Adverse event
ASC	Antibody-secreting cell
ALS	Antibody Lymphocyte Supernatant
BIgG	Bovine Immunoglobulin
B7A	B7A ETEC strain
B/P	Blood Pressure
C	Celsius
CFR	Code of Federal Regulations
CF	Colonization Factor
CFA	Colonization Factor Antigen
CFU	Colony Forming Unit
CIR	Center for Immunization Research
cGMP	Current Good Manufacturing Practice
cm	Centimeter
CS	Colonization Surface Antigen
Da	Daltons
DoD	Department of Defense
eCRF	Electronic case report form
EDC	Electronic data capture
ETEC	Enterotoxigenic *E coli*
F	Fahrenheit
FDA	US Food and Drug Administration
F/U	Follow Up
GCP	Good Clinical Practice
HBsAG	Hepatitis B surface antigen
HIV	Human immunodeficiency virus
HR	Heart Rate
IB	Investigator's Brochure
ICH	International Conference on Harmonization
IgA	Immunoglobulin A
IgG	Immunoglobulin G
IgM	Immunoglobulin M
IM	Intramuscular(ly)
IND	Investigational New Drug
IRB	Institutional Review Board
LPS	Lipopolysaccharide
LT	Labile toxin
CT	Cholera toxin
MCB	Master Cell Bank
ORS	Oral Rehydration Solution
µg	Microgram
mL	Milliliter
NMRC	Naval Medical Research Center
PBF	WRAIR Pilot Bioproduction Facility

PCB	Production Cell Bank
PBMC	Peripheral blood mononuclear cell
PI	Principal investigator
PVT	Psychomotor Vigilance Testing
SAE	Serious adverse event
SAP	Statistical analysis plan
SOC	System Organ Class
SOP	Standard operating procedure
SSP	Study-specific procedure
USAMRMC	US Army Medical Research and Materiel Command
WRAIR	Walter Reed Army Institute of Research

5. Introduction

5.1. *Clinical significance*

Diarrhea is a significant medical problem globally yielding an estimated 1.3-4.6 billion annual cases [1, 2]. Infectious diarrhea causes significant acute morbidity (negatively impacting growth and cognitive development) and mortality in infants, young children, and vulnerable populations in resource-limited countries, and civilian and military travelers to these areas [3, 4]. According to the World Health Organization (WHO) diarrheal illness is the second leading cause of death in children under five years of age, is preventable and treatable (via sanitation, hygiene, and safe drinking water), accounting for 760,000 deaths per year in this age group attributed in part to malnutrition [1].

Traveler's diarrhea (TD) affects up to 60% of travelers [5, 6]. TD is commonly self-limiting, lasts 2-6 days [7], and resolves after a week in 90% of cases, with a minority of patients experiencing persistent or chronic diarrhea. Although generally a self-limiting illness, about 20% of travelers who experience diarrhea are bedridden for some period and approximately 40% change their itinerary in some way because of the illness [6]. Diarrhea can vary in severity from mild discomfort to severe dehydration and dysentery. Personal hygiene and field sanitation measures have been unsuccessful in eliminating the risk of (TD) [8-10]. For example, pre-travel education and counseling of individuals on reducing risk behaviors (e.g. avoid ice/tap water, undercooked meat, unwashed/unpeeled fruits/vegetables) is common practice, however, while this intuitively makes sense, multiple studies have failed to show any consistent reduction in disease incidence [11, 12].

Bacterial enteropathogens comprise the majority of the pathogens identified in TD (civilian and military) encompassing upwards 80% of identified cases [13], with Enterotoxigenic *Escherichia coli* (ETEC) consistently the most identified. Additionally, ETEC is the most common bacterial etiology of infectious diarrhea in endemic pediatric populations accounting for 30 - 50% of diarrheal episodes [14-16]. TD incidence rates reach 0.5 episodes per person over 1 - 2 weeks of initial exposure in developing regions [14-18]. ETEC is culpable in an estimated 400 million cases and 160,000 deaths annually among infants and young children [19]. ETEC may be the first enteric illness encountered by infants [20] and the heavy burden of illness early in life contributes to malnutrition, which can then lead to growth stunting and diminished cognitive development [18]. In 2010 ETEC associated Disability-Adjusted Life Years (DALYs) were estimated at 8.5 million (10 percent of all diarrhea DALYs), and Years Lived with Disability (YLDs) were estimated at one million (13 percent of all diarrhea YLDs) [21, 22]. ETEC exposures occur through ingestion of contaminated food and water, typically producing non-invasive, watery diarrhea, although may manifest with a spectrum of disease presentations (based on strain virulence characteristics), ranging from mild diarrheal episodes to severe, cholera-like purging (even in immunocompetent hosts).

5.2 Diarrhea in the Military

A unique subset of vulnerable travellers is the military. Military associated diarrheal illness (essentially TD occurring in deployed military) has consistently been reported in deployed military personnel and remains the leading cause of disease non-battle injury (DNBI) accounting for a significant reduction in operational readiness, and mission capability [23] particularly for

deployments to the developing world. Among military personnel mortality has decreased (compared to historical controls), however, there remains significant morbidity, and a clear impact on operational readiness [24]. For historical perspective, data suggests that during the U.S. Civil War, 21,000 military deaths were attributable directly to dysentery. During the Korean War, approximately 80,000 duty-days were lost due to diarrhea and dysentery. During the Vietnam War, hospital admission rates or confinement to quarters due to diarrheal illness was higher than malaria by a 4:1 ratio, making diarrhea the most burdensome disease of that conflict [25]. Up to 70% of deployed U.S. personnel in support of Operations Enduring Freedom and Iraqi Freedom reported diarrheal episodes and 30% had three or more episodes with some units experiencing a monthly incident rate of up to 60% [26, 27]. Forty percent of UK forces in Afghanistan suffered at least one episode of diarrhea during their tour contributing to significant operational impact [28] with up to 43,000 man-days lost to 'no duty' or 'reduced performance' during the six months between April and October 2009 [28]. UK military data from Kenya has shown up to a 60% attack rate over a 6 week exercising period [28]. Diarrheal disease continues to be of significant military relevance as large numbers of young service members are deployed to areas with high TD rates [23]. From a military public health standpoint, its acute impact on troop health is larger than any other infectious disease syndrome and is compounded by the chronic risk of significant post-infectious sequelae [27-30]. The most cost-effective response to this military readiness threat is to prevent the exposure leading to diarrhea. The military has developed extensive capabilities for the provision of sanitation and hygiene, and clean food and water. This strategy is reasonably effective when it is possible to develop the proper infrastructure, but it is often undermined during rapid deployments and during small scale and brief operations. In large scale deployments conducted under strict security measures that prohibit routine exposure to indigenous food and water, diarrhea remains a serious problem. During the joint multinational military exercise conducted in Egypt (Operation Bright Star '01) under stringent security conditions, 9% of personnel reported developing diarrhea [30]. Controlling the base area infrastructure may be possible but patrolling patterns in high-risk areas often involves exposure to local pathogens.

Therefore, development of a safe and effective vaccine is needed to reduce the impact of ETEC disease on deployed military personnel and has been deemed a high priority by the U.S. military, as ETEC diarrhea has the potential to curtail critical overseas missions.

5.3 Pathogenicity of ETEC

The pathogenesis of ETEC diarrhea involves the sequential steps of colonization (via colonization factors (CF) promoting intestinal adherence) followed by secretogenic toxin production. Colonization ensues via the proteinaceous adhesive fimbrial surface-exposed polymeric protein appendages (the CF) potentiating microorganism attachment to the human intestinal epithelial cell contributing to infectivity and pathogenicity (interfering with intestinal physiology including motility) [31]. Upon colonization, ETEC secretes one or both of two enterotoxins that induce fluid and electrolyte secretion (by differing pathways) resulting in watery diarrhea. The two enterotoxins produced by ETEC are heat-stable enterotoxin (ST) and heat-labile enterotoxin (LT). To be classified as ETEC, *E. coli* must express one or both toxins. ST is a low molecular weight, nonimmunogenic peptide, and LT is a bipartite oligomeric protein that is structurally and functionally related to cholera toxin (CT) with 80% homologous amino acid sequencing [32-34]. ST is present in 75% of ETEC, either alone or with LT, and correlates with ETEC associated

diarrhea via activation of guanylyl cyclase. LT is composed of an A subunit that carries the enzymatic activity and five B subunits that bind to the receptor. The A subunit causes an increase in cAMP inside epithelial cells and results in fluid secretion.

5.4 ETEC Colonization Factors

To date, more than twenty-two serologically distinct CFs have been identified. Due to historical precedence and global prevalence, the classic CFs that were initially reported, CFA/I, CFA/II, and CFA/IV, have been most closely studied. CFA/I is a singular fimbrial structure, while CFA/II and CFA/IV are now known to comprise one or more distinct surface antigens. The CFA/II complex includes the thin fibrillar structure coli surface antigen 3 (CS3) expressed by itself or together with CS1 or CS2; and CFA/IV includes the structurally indistinct CS6 expressed alone or with CS4 or CS5. Based on classification by phylogenetic analysis of fimbrial usher proteins (FUP), many of the most prevalent ETEC CFs fall into either the α- or γ3-clade 34. The α-clade includes eight ETEC Class 5 fimbriae [35], which are composed of a major subunit that polymerizes to form a helical stalk and a tip-localized minor adhesive subunit that mediates adherence [36, 37]. Class 5 fimbriae share sequence similarities among their tip-localized adhesins more so than their major subunits [36], with the latter showing some subclass-specific serologic cross-reactivity [38, 39]. CS3 and CS6 are both atypical fimbriae in the FUP γ3-clade, each with two major subunits and no tip-localized adhesin, and no sequence similarities between the CS3 and CS6 major subunits. Based on meta-analyses of all available reports on ETEC CF prevalence and distribution, very conservative estimates indicate that ETEC Class 5 fimbriae in the FUP α-clade along with CS3 and CS6 (γ3-clade) are expressed by at least 70% of ETEC causing human disease [40]. The most commonly detected CFs of the α- and γ3-clade are CFA/I and CS6, respectively, which account for ~26% of all ETEC in travelers based on current evidence [40].

5.5 Evidence for anti-CF Immunity

CFs have long been a prime target for vaccine research and development. Their role as protective antigens has been substantiated by a number of studies in populations naturally exposed to ETEC diarrhea as well as volunteer studies of experimentally induced diarrhea, as has the role of LT enterotoxin [41-45]. Evidence for the preventive role of anti-CF immunity also derives from studies showing that milk with high levels of anti-CF antibodies passively protects newborn farm animals that are otherwise susceptible to diarrhea caused by ETEC bearing species-specific CFs [46, 47].

Additionally, oral administration of bovine milk immunoglobulins (BIgG) with specific activity against ETEC CFs has protected human subjects in the volunteer challenge model. In a landmark study, Tacket *et al* found that a colostral BIgG concentrate derived from cows immunized with a cocktail of killed whole-cell ETEC (including CFA/I-ETEC) conferred 100% protection to volunteers challenged with the prototype ETEC strain H10407 (CFA/I; LTST; O78:H11) [48]. Subsequently, it was shown that BIgG derived from the milk of cows immunized with purified CFA/I conferred 90% protection against H10407 challenge in volunteers [49], indicating the primacy of CFA/I as a protective antigen. Most recently, under funding from the Peer Reviewed Medical Research Program, we evaluated the efficacy of (BIgG) preparations made against CFA/I and CS17, two Class 5 fimbriae. In brief, both anti-CFA/I BIgG and anti-CS17 BIgG conferred

significant protection against ETEC challenge strains expressing homologous CFs. These studies further support CFs of this class as protective antigens.

Despite the robust evidence supporting the CFs from class 5 as protective antigens, there is a dearth of evidence on other CF types, such as CS6. Since its first description in 1985 [39], CS6 has been the focus of considerable research, yet the generation of incontrovertible evidence as to its specific role in diarrhea pathogenesis and protection against re-infection is lacking. One clear and consistent finding is that CS6, expressed most often alone but also with CS5 or CS4, is one of the most common CFs associated with symptomatic ETEC infection in both endemically exposed populations as well as travelers [40, 45, 50-57]. This has driven the focus on CS6 as a target for many groups working in vaccine development [58-62]. The majority of individuals naturally infected with CS6- ETEC exhibited mucosal and serological responses against CS6 [63] as well as CS6-specific B-cell memory responses [64], while naive subjects experimentally infected with CS6-ETEC showed less robust mucosal and serological responses [65]. In limited investigations, however, serum anti-CS6 antibody titers did not show a protective relationship for subsequent CS6-ETEC diarrhea [66]. While these findings indicate that CS6 is recognized by the host during infection, we have little understanding regarding bacterial regulation of CS6 expression in vivo [67]. Considering our current body of knowledge, the epidemiological importance of CS6 stands in sharp contrast with the absence of consistent, credible proof that CS6 serves as a protective antigen. This is the first of two studies to begin assessing the protective capacity of CS6 in passive prophylaxis studies by making use of the experimental human challenge model for ETEC strain B7A, which expresses this epidemiologically important CF. The goal of the study described herein is to re-establish and refine the experimental human challenge model for B7A.

5.6 History of the ETEC Human Challenge Model

Over the past 40 years, the enterotoxigenic *E. coli* (ETEC) human challenge model has been used to elucidate the pathogenesis and immune responses associated with ETEC infection as well as to test the efficacy of investigational drugs and vaccines. The initial experimental infection, published in 1971, was a landmark study establishing ETEC as the organism responsible for causing acute, cholera-like illness in a U.S. soldier in Vietnam [68]. In this classic paper, researchers demonstrated that while porcine and human isolates of disease-causing *E. coli* were both capable of inducing fluid excretion in rabbit ileal loops, only human isolates were capable of causing disease in human subjects. It was later discovered that the difference in the two strains was the species-specific tropism of the intestinal colonization factor fimbriae. One of the strains in that original study was B7A, a CS6-expressing, LT+, ST+ strain of ETEC.

Since that landmark study, approximately 600 naïve subjects have been administered ETEC in an experimental infection. One of the three most common strains administered is the aforementioned B7A strain (CS6+, LT+, ST+. O148:H28). To date, this strain has been safely administered at doses ranging from $1x10^8$ to $1x10^{10}$ cfu yielding diarrhea attack rates from <25% to 100% [69]). Most recently, in a study conducted at the US Army Medical Research Institute for Infectious Disease (USAMRIID) using a B7A cell bank produced under cGMP conditions at the WRAIR, this strain induced moderate-severe diarrhea attack rates (often the primary outcomes for vaccination/ challenge studies) of 37.5 and 100% at doses of 10^9 and 10^{10} cfu, respectively [65]. In all prior studies, there have been no 'related' serious adverse events and all 'related' adverse events have

been consistent with the acute diarrheal illness (with associated signs and symptoms) anticipated from an experimental infection with virulent ETEC. In least in one prior study, it was observed that initial experimental infection with the B7A strain protected subjects against re-challenge with the same organism ~9wks later [70]. The B7A strain is sensitive to most commonly used antibiotics, including ciprofloxacin and amoxicillin, and is readily cleared following a routine 3-day course of antibiotics. Thus, B7A potentiates the safe development of diarrheagenic illness, assessment in efficacy in prophylactic interventions and confident treatment efficacy after completion of the evaluation.

One concern regarding this and other ETEC challenge models is the high dose of inoculum required to induce sufficient disease rates to facilitate evaluation of a vaccine in reasonable numbers of subjects. A B7A inoculum of 10^{10} cfu may not be reflective of the average inoculum in naturally acquired infection and may in turn skew efficacy results towards the null in a volunteer assessment. In addressing this concern with another ETEC challenge strain, H10407 (CFA/I-ETEC), a refinement of the model was instituted whereby implementation of an overnight fast (in place of the typical 90 minute fast before challenge) resulted in reproducible attack rates among subjects with inoculum doses 2 logs below previously required doses [71]. Following along with those studies, we plan to establish the B7A model at JHU using the most recently administered doses (10^9, 10^{10} cfu) and fasting regimen (90 minutes) while simultaneously assessing the effect of an overnight fast on moderate-severe diarrhea rates following challenge. A higher diarrhea attack rate following an overnight fast compared to the traditional 90 minute fast is anticipated based on the results from recent studies with H10407. Subsequently, we will validate the optimal dose and fasting regimen identified in a rechallenge cohort. The optimal challenge regimen will be utilized in the follow-on passive, oral vaccination-challenge study.

6. Study Objectives

The overarching objective of this study is to identify a safe and optimal dose and regimen (fasting duration) for administering the challenge ETEC strain B7A (O148:H28 $CS6^+$ LT^+ST^+) (Lot 0481). Additionally, an assessment of homologous protection following rechallenge with B7A will be assessed. These data will be utilized to determine the optimal dosing regimen for use in a subsequent study to assess the efficacy of passive immunoprophylaxis with anti-CS6 and anti-B7A killed whole cell bovine antibodies.

6.1. *Primary Objectives and Endpoints*

1. Evaluate the safety of the challenge model.
2. Identify a dose and fasting regimen that induces moderate-severe diarrhea in at least 70% of naïve subjects without causing high output diarrhea (determined by stool output volumes or signs and symptoms associated with hypovolemia)
3. Assess protection upon repeat exposure to the homologous ETEC strain (applying previously determined orally administered challenge inoculum), specifically [B7A (O148:H28 $CS6^+$ LT^+ST^+) (Lot 0481)]

The primary endpoint for this study is moderate-severe diarrhea defined as follows post-inoculation:

- Moderate diarrhea: 4 to 5 loose/liquid stools or 401-800 g of loose/liquid stool in any 24-hour period
- Severe diarrhea: ≥ 6 loose/liquid stools or > 800 g of loose/liquid stool in any 24-hour period

6.2. *Secondary Objectives and Endpoints*

1. Measure mucosal and systemic immune responses to experimental infection
2. Obtain and archive samples for future proteomics, microbiome and/or systems biology efforts
3. Compare B7A shedding levels in subjects infected for the first time with B7A and in those re-challenged with the homologous strain

Secondary endpoint measures:
A number of secondary endpoints will be determined in this study. Specific endpoints have been selected to support the primary outcome and are outlined below.

1. Maximum 24-hour stool output
2. Percent of subjects with severe diarrhea
3. Percent of subjects with diarrhea of any severity
4. Total weight of grade 3-5 stools passed per subject over inpatient period
5. Number of grade 3-5 stools per subject
6. Percent of subjects with nausea, vomiting, anorexia, or abdominal pain/cramps rated as moderate to severe
7. Mean/Median time to onset of diarrhea
8. Number of subjects with moderate to severe ETEC illness
9. Number of colony forming units of the challenge strain per gram of stool 2 and 4 days after challenge
10. ETEC systemic and diarrhea severity score post-challenge

6.3. *Exploratory Objectives*

1. Exploratory immunology and systems biology analyses to include transcriptomics, proteomics, phosphoproteomics, and immune profiling
2. Exploratory evaluation of the cognitive impact of acute diarrhea using a wrist-worn actigraph and psychomotor vigilance testing
3. Evaluate the impact of both the B7A ETEC challenge and antibiotic exposure on short-term changes in host microbiota.
4. Explore the impact of the microbiome on disease susceptibility.
5. Evaluate the impact of the B7A ETEC challenge on short-term changes in intestinal inflammation/repair, epithelial barrier function, motility, and immune system modulation.

7. Trial Design

7.1. *Study Design*

This study is separated into two cohorts. The first cohort is designed as an open-label dose-finding study in which a CS6-ETEC strain (B7A) will be administered to four groups as indicated in Table 3. The objective is to identify the lowest inoculum dose and fasting period that induces a moderate-severe diarrhea attack rate of ≥70% without producing high output diarrhea. Subjects (n=28) will be assigned to receive one of three doses of CS6-ETEC strain B7A with either an overnight (10^8 and 10^9 cfu) or a 90-minute (10^9 and 10^{10} cfu) pre-inoculation fast. The lowest dose and fasting regimen combination will be identified from the initial cohort and utilized in an additional 15 naïve subjects (Cohort 2) to validate the moderate-severe diarrhea attack rates (n=15). Concurrently, up to 15 veteran subjects who meet the primary outcome of moderate-severe diarrhea from Cohort 1 will be re-challenged with B7A to assess protection from homologous re-challenge. For cohort 1, subjects will be assigned in a 1:1:1:1 ratio to one of 4 study groups.

Table 3. Study Design

Cohort	N	Dose (cfu)	Pre-dose fasting time
1	7	10^8	Overnight
	7	10^9	90 minutes
	7	10^9	Overnight
	7	10^{10}	90 minutes
2*	15	Naïve subjects	
	15	Veteran subjects from Cohort 1meeting the primary endpoint	

*Note: The total number of subjects enrolled in Cohort 2 will be ≤30 dependent on the number of veteran subjects available for re-challenge. In the event that the available number of veteran subjects is <15, efforts will be made to increase the number of naïve participants to provide more confidence in disease rates in subsequent applications of the model. The challenge administered to the cohort 2 subjects will be the optimal dose and fasting duration identified from the cohort 1 study. In cohort 2, B7A veterans will be re-challenged with B7A at approximately 1-8 months after their initial infection.

Subjects will be admitted to the JHU CIR Bayview facility the day prior to inoculation (day -1) and will remain in the inpatient facility until meeting all discharge criteria. The Western Institutional Review Board (IRB) and IRB at the Naval Medical Research Center will review and approve the protocol prior to initiation. Subjects meeting pre-set criteria will be treated with antibiotics. Subjects not receiving early antibiotic treatment will start antibiotic treatment on study day 5. Routine discharge is scheduled for day 8, when most subjects are expected to meet the discharge criteria: they feel well (clinical symptoms resolved or resolving), have taken at least one dose of antibiotic and have two consecutive stool cultures negative for the challenge strain. Subjects may be discharged earlier than day 8 on a case-by-case basis if they meet discharge criteria. Subjects will return for at least one outpatient visit (more if discharged early) to assess for any new adverse events or to continue follow-up of previously identified adverse events and to have samples collected for immunology testing. Additionally, approximately 6 months following experimental infection, subjects will have a telephone call to assess for any new onset serious adverse events. The second cohort of the study will be separated in time from the first cohort by 1-8 months.

7.2. *Recruitment*

The CIR at JHBSPH recruits volunteers from the greater Baltimore, Washington DC and Philadelphia regions. Frequently, subjects are recruited from as far as the New York City metropolitan area and New Jersey.

Newspaper ads and study fliers posted on the JHU campus and community bulletin boards will be used to recruit prospective subjects. Additionally, subjects in previous studies that have expressed interest in participating in future trials will be contacted about the proposed study. All study specific-related advertisements will be reviewed and approved by the WIRB, NMRC IRB and HRPO-ORP. Subjects responding to the advertisements by a phone call to the center will be screened for eligibility based on a standard screening questionnaire administered by the CIR recruiter. Some elements of the inclusion/exclusion criteria will be discussed with the subject at that time and a preliminary determination will be made regarding the individual's eligibility for study participation.

7.3. *Screening*

The CIR may use a screening protocol approved by the Johns Hopkins School of Public Health (JHSPH) Institutional Review Board (IRB) in recruiting volunteers for this study. The screening protocol is entitled "Screening of adult volunteers for eligibility to participate in clinical studies evaluating investigational vaccines, antimicrobial agents, or disease prevention measures or the pathogenesis of infectious agents" JHSPH IRB 200, JHSPH IRB H.22.04.02.19.A2. Volunteers will be made aware that the screening process may take several visits to complete. Using this screening protocol, a medical history/exam and a series of clinical laboratory tests may be completed to rule out occult illness and pregnancy. These laboratory tests may include, but are not limited to complete blood count (CBC), serum chemistries, hepatitis B antigen, hepatitis C antibody, HIV-1 antibody, IgA levels, serum HCG (for females of childbearing potential), and urine toxicology (drug screening). (Confirmatory testing will be performed on subjects who test positive for hepatitis B, hepatitis C, or HIV-1 antigens.) Subjects who have ≤ 2 mild (grade 1) abnormalities may be included if the principal investigator determines that their participation will not present undue risk to the subject. Subjects with > 2 mild abnormalities may be included in the study at the discretion of the principal investigator. Subjects with clinical laboratory abnormalities of greater than mild severity will not participate in this clinical trial. The clinical toxicity grading scale that will be used as a guideline is based on the Guidance from the FDA Center for Biologics Evaluation and Research (CBER). If any additional safety labs are performed, the FDA Guidance for Industry will be utilized.

Additionally, samples for ABO and RH blood typing will be collected following recent data suggesting correlation between ABO typing and susceptibility to moderate to severe diarrhea following challenge with ETEC strain H10407 (Fleckenstein, unpublished). RH typing has been previously reported to affect clinical outcomes in subjects infected with other enteric pathogens and as such similar associations will be assessed as part of this study [72-74].

Potential volunteers will be given a complete description of the study. To ensure comprehension of the study, all volunteers will have to pass a written examination before inclusion in the study. Volunteers who meet all inclusion criteria and none of the exclusion criteria, pass the comprehension test, and sign the study Informed Consent Document (ICD) may be eligible for the study. Details on administration of the comprehension test are found in the next section in the discussion of selection of subjects for participation.

Subjects screened and enrolled in Cohort 1 will be consented prior to participation for the potential to be enrolled in Cohort 2 as veteran subjects to assess homologous protection upon re-challenge. Their eligibility for 'Cohort 2' will be dependent on their successful completion of Cohort 1 as well as meeting the primary outcome following their initial infection. Subjects enrolled as naive participants in Cohort 2 will not be consented for a subsequent re-challenge.

Informed consent is an ongoing process which includes the informed consent document. Subjects will receive an oral presentation of the study. Each prospective subject will be given the written, IRB-approved informed consent, allowed ample time to read the consent, allowed to ask questions about the study, have his/her questions answered, and given time to decide if he/she would like to participate in the study. To document subjects' understanding of informed consent, immediately before the consent is signed, the person obtaining consent will administer a brief quiz or comprehension test. Incorrect answers will be discussed with subjects to reinforce the consent and subjects will be given one additional opportunity to take the test. Subjects who fail the comprehension test on the first attempt may retake the comprehension test on the same day, or they may come back on a separate visit to retake the test. A final acceptable test score is 70% or more answered correctly. Subjects failing after 2 attempts are not eligible for study enrollment. No coercion or influence is allowed in obtaining subjects' consent. Before subjects participate in the study, consent forms will be signed and dated by subjects as well as by the PI or designee. Subjects will receive copies of the signed consent prior to participation. As part of the consent process, subjects will also be asked to read and sign a Medical Records/Lab Results Release, with an opportunity to ask questions, if relevant. Additionally, subjects will be asked to complete a Functional Bowel Disorder Survey (Rome III) to assess general GI health (survey administered by study staff).

7.3.1. Inclusion criteria

1. Male or female between 18 and 50 years of age, inclusive.
2. General good health, without clinically significant medical history, physical examination findings or clinical laboratory abnormalities per clinical judgment of the PI
3. Completion of a training session and demonstration of comprehension of the protocol procedures and knowledge of ETEC-associated illness by passing a written examination (passing grade ≥ 70%)
4. Willing to participate after informed consent obtained.
5. Availability for the study duration, including all planned follow-up visits.
6. Negative pregnancy test with understanding (through informed consent process) to not become pregnant during the study or within three months following last scheduled study visit. Females of childbearing potential must agree to use an efficacious hormonal or barrier method of birth control during the study. Abstinence is acceptable. Female subjects unable to bear children must have this documented (e.g. tubal ligation or hysterectomy) or must have negative pregnancy tests. Effective methods of avoiding pregnancy (including oral, topical or implanted contraceptives, IUD, female condom, diaphragm with spermicide, cervical cap, abstinence, use of a condom by the sexual partner, or sterile sexual partner) prior to dosing of the ETEC challenge strain.

7.3.2. Exclusion criteria

General health criteria

1. Presence of a significant medical condition (e.g., psychiatric conditions; gastrointestinal disease, such as peptic ulcer, symptoms or evidence of active gastritis/dyspepsia, inflammatory bowel disease, irritable bowel syndrome (as defined by the Rome III criteria or medical diagnosis); alcohol or illicit drug abuse/dependency) which in the opinion of the investigator precludes participation in the study. Some medical conditions which are adequately treated and stable would not preclude entry into the study. These conditions might include stable asthma controlled with inhalers or mild hypertension stably controlled with a single agent.
2. Significant abnormalities in screening hematology, or serum chemistry as determined by PI or PI in consultation with the research monitor and sponsor.
3. Evidence of confirmed infection with HIV, Hepatitis B, or Hepatitis C.
4. Evidence of IgA deficiency (serum IgA < 7 mg/dL or below the limit of detection of assay).
5. Evidence of current excessive alcohol consumption or drug dependence (a targeted drug screen may be used to evaluate at the clinician's discretion).
6. Evidence of impaired immune function.
7. Recent vaccination or receipt of an investigational product (within 30 days before receipt of challenge).
8. Any other criteria which, in the investigator's opinion, would compromise the ability of the subject to participate in the study, the safety of the study, or the results of the study

Research Related Exclusions Applicable to Challenge Participation

9. History of microbiologically confirmed ETEC or cholera infection in last 3 years.
10. Occupation involving handling of ETEC or *Vibrio cholerae* currently or in the past 3 years.
11. Symptoms consistent with Travelers' Diarrhea concurrent with travel to countries where ETEC infection is endemic (most of the developing world) within 3 years prior to dosing, OR planned travel to endemic countries during the length of the study.
12. Vaccination for or ingestion of ETEC, cholera, or *E. coli* heat labile toxin within 3 years prior to dosing.
13. Any prior experimental infection with ETEC strain B7A

Study-specific Exclusion Criteria (potential increased risk or complicating outcome ascertainment)

14. Abnormal stool pattern (fewer than 3 per week or more than 3 per day).
15. Regular use of laxatives, antacids, or other agents to lower stomach acidity.
16. Use of any medication known to affect the immune function (eg, systemic corticosteroids and others) within 30 days preceding the administration of challenge or planned use during the active study period.
17. Known allergy to two of the following antibiotics: ciprofloxacin, trimethoprim-sulfamethoxazole, and amoxicillin.

Eligibility for proceeding to the second challenge after completing the first challenge

18. Must continue to meet inclusion criteria above (including repeat of some safety labs)

19. Must not meet any of the exclusion criteria 1-8 and 14-17 listed above (prior ETEC exposure no longer exclusionary due to prior challenge)
20. Must have met the primary endpoint of moderate-severe diarrhea
21. All study subjects with serious adverse events from the primary challenge will be excluded from a repeat (second) challenge.

Additionally, although not a specific exclusionary criterion, subjects who intend to donate blood products within 1 month of study participation should not enroll in the study. Subjects will be informed of this requirement during the informed consent process and they will have the option not to participate. They will also be instructed, that Red Cross policy may be to reject blood donation for up to a year after participation in a research study.

7.4. *Day -1*

The day of admission, subjects will be evaluated to ensure no exclusionary conditions have arisen and baseline exam and laboratory evaluations obtained. Subjects will undergo vital signs assessment, review of medical history, and physical examination. A serum pregnancy test (βhCG) will also be obtained from women that have childbearing potential. If the result of a serum pregnancy test is not available on day of challenge, a urine pregnancy test will be conducted. Additionally, samples will be collected for assessment of immune outcome (and exploratory) measures. Stool may be collected for bacteriology, exploratory, and microbiome assays as per schedule of events. Subjects will also receive instruction on the wear of the wrist actigraph and execution of 5-minute PVT.

Subjects will be offered a snack at approximately 11:00 pm. Subsequently, subjects in the overnight fasting arm will initiate their fasting period until 90 minutes after challenge. Subjects assigned to the 90 minute fasting groups will be able to eat and drink until 90 minutes before challenge and their fasting will continue until 90 minutes after the B7A challenge

7.5. *Challenge*

On the day of challenge, subjects assigned to the 90 minute fasting period will receive a light breakfast and then initiate an approximate 90 minute fasting period. Subjects assigned to the overnight fasting arm will continue their overnight fast. Approximately 1 minute prior to challenge, subjects will drink 120 mL of bicarbonate buffer (buffer formulation: 13.35 gram of sodium bicarbonate in 1000 mL of sterile water for irrigation). For challenge, subjects will drink a solution of virulent B7A bacteria suspended in the remaining 30 mL of bicarbonate buffer at the appropriate inoculum doses. All subjects will continue fasting for at least an additional 90 minutes post challenge.

7.6. *Inpatient monitoring*

Subjects will remain at the inpatient facility under clinical observation. Vital Signs (VS) will be assessed at least 3 times each day, once in the morning, in the afternoon and at bedtime. On challenge day, VS will be assessed 4 times, once prior to challenge, once about 30 minutes after challenge, and then 2 additional times this day. A clinician will conduct a daily medical interview

to assess health status, follow-up, monitor, and treat as indicated. All stools will be collected for weighing and grading. Following ETEC B7A challenge, up to 3 stool samples will be collected daily for culture starting the day after challenge. If a subject is unable to provide a stool sample by 1300 hours, s/he will be asked to obtain a rectal swab (swab will be obtained at 1300). Swabs will be used starting the day after challenge.

Subjects will perform 5-minute PVT tests at least three times a day during the inpatient phase using the wrist actigraph. As an exploratory assessment, performance of the three PVTs per day will be predicated on the subject not undergoing other procedures or primary study related events. Missed PVTs will not be considered protocol deviations. Similarly, management of symptoms associated with ETEC or other illness will have priority over completion of PVTs.

Antibiotic treatment after challenge will be administered according to criteria for early antibiotic treatment (described below) or 5 days after challenge if subjects do not meet the criteria for early treatment. Subjects will be treated with an antibiotic [ciprofloxacin (500 mg by mouth twice daily for 3 days)], except in cases of known allergy or intolerance in which case a suitable alternative will be utilized. Alternative treatments are trimethoprim 160 mg / sulfamethoxazole 800 mg by mouth twice daily for three days, or amoxicillin (500 mg by mouth 3 times daily for 3 days). Subjects will be discharged from the inpatient facility when they are well and after having 2 consecutive stool cultures which are negative for the challenge strain. Routine treatment will commence at about 120 hours (on the morning of day 5) post-challenge. If, because of illness, a subject is unable to take oral antibiotics, intravenous antibiotics may be given (IV ciprofloxacin at an appropriate dose based on weight and clinical status).

Treatment for vomiting may be needed. Subjects who are vomiting may be given ondansetron (Zofran) ODT or ondansetron IV for the management of vomiting.

Early antibiotic treatment after challenge will commence when any of the following criteria are identified and a study physician considers it to be warranted:

- Severe diarrhea (based on volume, 800 g, or earlier if study physician considers it warranted)
- Stool output consistent with moderate diarrhea for 48 hours
- Mild or moderate diarrhea and 2 or more of the following symptoms: severe abdominal pain, severe abdominal cramps, severe nausea, severe headache, severe myalgias, any fever (≥ 38.0°C), or any vomiting.
- A study physician determines that early treatment is warranted for any other reason

Symptoms infrequently associated with ciprofloxacin include nausea, vomiting, diarrhea, abdominal pain, rash, headache or restlessness. Amoxicillin is generally well tolerated and the most frequently reported adverse reactions are diarrhea and rash.

Rehydration Procedures: Subjects passing grade 3-5 stools post-challenge will be offered ORS, an oral glucose/electrolyte solution or Gatorade to prevent dehydration, at the same volume as their stool output.

A subject may be administered IV fluids (clinician discretion) if they:

- Experience abrupt onset of diarrhea, defined as passage of an initial loose/liquid stool of >

300 g, or > 400 g of loose/liquid stools over 2 hours in conjunction with other symptoms, as determined by PI or designee

- Become hypovolemic, defined as confirmed supine systolic BP < 90 mmHg and associated symptoms, or significant lightheadedness on standing, with a confirmed postural change in BP or pulse. Postural vital signs will be measured lying and 2 minutes after standing. A significant change is a decrease in systolic BP of > 20 mmHg, or diastolic BP of > 10 mmHG or increase in pulse of > 30 beats/minute
- If determined necessary by the study physician; eg, diarrhea with nausea/vomiting and unable to drink enough to keep up with output, or other reason

Administration of Oral Rehydration Solution (ORS)
CeraLyte 50 (CeraProducts, Jessup, MD) is a rice-based oral electrolyte solution that will used to help control/prevent dehydration among subjects experiencing ETEC diarrhea. CeraLyte 50 is packaged in 10 g sachets to be dissolved in 200 mL (~ 1 cup) tap water. The contents per 10 g sachet are as follows:

- Sodium chloride - 230 mg
- Potassium chloride - 156 mg
- Trisodium citrate - 378 mg
- Carbohydrates - 9.4 g

On a per-liter basis, CeraLyte 50 provides 50 mEq of sodium chloride and 20 mEq of potassium at a mOsm < 250.

For documentation purposes of concomitant medications, ORS will not be considered a concomitant medication while IV fluids will.

Routine discharge is scheduled for study day 8. Two consecutive negative stool cultures for B7A are required before discharge (can be collected on the same study day). Remaining doses of antibiotic will be given to the subject for self-administration. VS at discharge will be recorded in the source documents and in the electronic CRF.

Early discharge is permitted in cases where early antibiotic treatment has been initiated. The subject needs 2 stool cultures negative for B7A and to have taken two doses of antibiotic with resolved or resolving clinical symptoms before discharge. Remaining doses of antibiotic will be given to the subject for self-administration. Subjects discharged before study day 7 will return on day 7 and provide the requisite samples (stool, blood) as delineated in the T&E table.

Outpatient Follow-Up: After discharge, challenge subjects will have outpatient follow up in the clinic on day 28 (+/- 3). In addition, subjects will also have a single phone follow-up on day 180 (+/- 1 month). Clinic visits during follow-up will include vital signs assessment, clinical checks and sample collection for immunogenicity and exploratory outcome evaluation. Subjects will be asked questions from the Function Bowel Disorder Survey (Rome III) during the telephone follow-up at day 180 to assess for any new health issues occurring in the months after the study. Subjects with new onset functional bowel disorders will be asked to report to the JHU CIR for an in-person follow-up visit and counseling, however, it is not expected that subjects will develop any chronic symptoms.

7.7. *Outcome verification and rechallenge*

A cohort of naïve subjects will be recruited for Cohort 2 to verify the dose and fasting regimen identified from the initial cohort. Additionally, previously challenged veteran subjects who met the primary endpoint of moderate-severe diarrhea will be rechallenged as part of Cohort 2. The study scheduled for those subjects is as outlined above. These subjects will be rechallenged at approximately 1-8 months after their initial diarrheal illness due to B7A.

7.8. *Concomitant medication*

Only concomitant medications approved by the study physician will be used during the study period. As the subjects will stay in the inpatient facility after challenge until treatment, this should not be an issue. Subjects taking regular medication (ie, birth control pills) prior to enrollment in the trial will be allowed to continue to take this medication unless it is specifically excluded as part of the inclusion/exclusion criteria for the trial. Subjects needing to take unapproved or excluded medication will not be eligible for enrollment in this study. Any medication ordered by the study physician during the course of the trial will be documented on appropriate source documents. Approved medications being taken prior to and during the course of the trial will also be documented in this manner.

7.9. *Handling of study samples*

Samples collected under this protocol will be used to conduct protocol-related safety and immunogenicity evaluations. Samples for immunogenicity will be collected at the JHU CIR and maintained until transport to NMRC. Storage at NMRC of these biological samples will be handled according to appropriate procedures. Any study for the future use of these biological samples will have IRB approval. All subjects will consent for the future use of their specimens.

7.10. *Outcome measures*

7.10.1. Clinical

The primary endpoint of this study is moderate to severe diarrhea according to the following definitions post-inoculation:

1. Severe diarrhea: ≥ 6 grade 3-5 stools in 24 hours, or > 800 g of grade 3-5 stools in 24 hours and,
   Moderate diarrhea: 4-5 grade 3-5 stools in 24 hours or 401-800 g of loose/liquid stool in any 24-hour period

Stool will be graded based on a standard stool grading scale as follows:

Grade 1 = Fully formed (normal)
Grade 2 = Soft (normal)
Grade 3 = Thick liquid (diarrheal)
Grade 4 = Opaque watery (diarrheal)

Grade 5 = Rice-water (diarrheal)

Additional secondary endpoints have been selected as follows:

- Maximum 24-hour stool output
- Percent of subjects with severe diarrhea
- Percent of subjects with diarrhea of any severity
- Total weight of grade 3-5 stools passed per subject over inpatient period
- Number of grade 3-5 stools per subject
- Percent of subjects with nausea, vomiting, anorexia, or abdominal pain/cramps rated as moderate to severe
- Mean/median time to diarrhea onset
- Number of subjects with moderate to severe 'ETEC illness'
- Number of cfu of the challenge strain per gram of stool 2 and 4 days after challenge
- ETEC systemic and diarrhea severity score post-challenge with B7A

An exploratory assessment of the cognitive impact of ETEC challenge will be conducted with the use of continuous wrist actigraphy/PVT monitoring and laptop-derived PVT monitoring. These outcomes will not be utilized as part of the regulatory, safety, immunogenicity, or efficacy evaluation of the study product, are exploratory in nature and will not be retained in the regulatory file. Subjects will be issued wrist actigraph/PVT device for use while inpatients. Wrist PVT 5-min tests per day will be performed by each subject up until discharge from the treatment facility as outlined in the SSP. Comparisons will be made between symptom presence/severity and adjusted for sleep cycles and other confounding variables. Additional exploratory assessments will include evaluation of immune activation and cognitive performance by use of systems biology parameters.

7.10.2. Immunological

Blood samples for immunology include obtaining serum and peripheral mononuclear cells (PBMCs) by appropriate collection tubes and processing methodology.

Serum samples will be assayed for IgG and IgA antibody titers against LT using the GM1-ELISA and against O148, LPS, and CS6 using methods previously established. Previously established high-titer specimens will be included on each plate to track day to day interassay variation and for standardization. For each antigen, pre- and post-challenge serum samples will be assayed side-by-side. The antibody titer assigned to each sample will represent the geometric mean of duplicate assay. Reciprocal endpoint titers < 50 will be assigned a value of 25 for computational purposes. Seroconversion will be defined as a > 4-fold increase in endpoint titer between pre-and post-challenge samples.

Qualitative (responder rates) and quantitative assessments (log transformed values) will be made in addition to evaluation of the kinetics of the immune response. Median increases (fold-rises) of anti-ETEC (i.e., CS6, LT, O148, LPS) antibody concentrations and seroconversion rates will be calculated along with 95% confidence intervals. Geometric mean titers will also be determined and presented with their 95% confidence intervals.

PBMCs will be assayed to determine antigen specific (CS6, O148, LPS and LT) ALS responses.

Antibody in Lymphocyte Supernatant (ALS) is a methodology that has been shown to be a replacement for ELISPOT methodology. PBMCs are incubated without stimulation and the supernatant is later assayed for antigen-specific IgG and IgA Abs by ELISA. It offers advantages over ELISPOT because it does not require a ELISPOT reader, the supernatant can be obtained and tests performed later, and the same supernatant can be used in multiple assays, which optimizes the number of cells obtained from volunteers. A positive ALS response will require a four-fold rise in antibody titers between pre and post challenge samples. For each antigen, pre-and post-challenge samples will be tested on the same plates.

Samples will also be collected to support additional exploratory evaluations in systems biology and immune profiling. Cells and serum samples will be collected for use in a variety of transcriptomic, proteomic, flow cytometry, memory B cells, and cytokine analysis.

7.10.3. Exploratory

Exploratory and expanded immunological assessments will be planned for this study. Among these, serum and PBMC samples will be collected for transcriptomic, cytokine, proteomic, and other systems biology analyses to identify molecular signatures associated with ETEC infection. The cytokine analyses will encompass representation from multiple pathways including pro-and anti-inflammatory, and regulatory pathways.
Antigen specific memory B cell quantification may be performed with purified PBMCs to investigate the response generated following oral challenge. Briefly, following an in vitro stimulation/expansion to enrich for memory B cells, they are finally quantified as Ag-specific ASC by ELISPOT to detect relative changes following experimental infection].

Fecal and salivary IgA samples will be obtained to assess for mucosal IgA [including but not limited to total and anti-CS6, anti-O148 (LPS), and anti-LT] (see schedule delineated in the Time and Events Table). Subjects will be provided stool hats to collect all stools which will be processed within 2 hours.

Collection of a saliva sample will be performed utilizing synthetic oral swabs (Salimetrics Oral Swab; SOS). The subject will place a single swab in their mouth under the tongue, to collect saliva (only the lingual area—not from the parotid) for several (approximately 10) minutes. Subjects will be instructed not to eat or drink anything, including chewing gum, for 10 minutes prior to saliva sample collection. Subjects will be instructed to avoid drinking alcohol or using mouthwash for 24 hours and to avoid caffeinated beverages for 12 hours prior to collecting the sample. Saliva collection vials will be pre-loaded with 10uL of 100X HALT Protease Inhibitor Cocktail. Immunologic responders will be defined as subjects with a $\geq$ two-fold increase in reciprocal endpoint titer.

In addition, stool samples will be obtained to assess for exploratory endpoints to include microbiome characterization, culture-independent methods to quantitate B7A shedding, and PCR and transcriptomics (on the microbiome). This testing is subject to change as advances in research occur during the time that the stool is archived. These samples will be collected per SSP.

7.10.4. Outcome Adjudication Committee

Blinding will not be utilized in the cohorts of the study. In an effort to obtain an unbiased determination of the efficacy outcomes, an independent outcome adjudication committee, the members of which will be blinded as to the dose and fasting regimens of the challenge volunteers, will evaluate challenge outcome data after study.

The committee will be comprised of 3 individuals, independent of the study sponsor and investigative team, who are experts on diarrheal illness case identification and pathogen diagnosis. The committee will also include an unblinded study statistician who will lead and coordinate the committee but will have a non-voting role in deliberations.

The committee voting members will review all potential efficacy-related cases and endpoint data but will be blinded as to the dose and fasting regimen of cases. Among the committee's responsibilities, they will (1) review and confirm all primary endpoint cases; (2) review all protocol-specified entry criteria, adherence, and compliance issues to ascertain classification in the per-protocol and other study populations; and (3) provide guidance regarding secondary and other endpoint classifications to include agreement on objective criteria for classification of endpoints. Specific duties and responsibilities will be outlined by charter prior to the start of the study.

8. Investigational product

The investigational product is ETEC strain B7A. It was manufactured at the WRAIR PBF in 1997. Each vial of the production cell bank contains approximately 9 x 10^8 cfu of live ETEC B7A in Luria Broth (LB) with 15% glycerol as cryopreservative. There is 1 ml of the bacterial suspension per vial. The lot number is 0481. Vials are stored at ≤-80±10°C. Bacteria are not given directly from the vials to volunteers; they are inoculated into media and grown overnight.

8.1. *Label*

The label is as follows:

Production Cell Bank for Enterotoxigenic *E. coli* CS6
Challenge Strain B7A
BPR No.: BPR-258-00 Lot No.: 0481
Contents: 1.0mL
Cautions: For Manufacturing Use Only; Viable Organism.
Date of Mfg.: 09 Oct 97 Storage: ≤-80 ± 10°C
Manufactured By: WRAIR, Washington, D.C. 20307

8.2. *Product characterization*

Table 4. Specifications of Production Cell Bank of B7A lot 0481

Sample Test	Specification	Result	Pass/Fail or N/A
Morphology	Cream colored, circular,	Cream colored, circular,	Pass

	smooth colony	smooth colony	
Gram Stain	Gram Negative Rod	Gram Negative Rod	Pass
Viability	For Information Use Only	8.7 x 10^8 CFU/ml	N/A
Purity	No Contamination	No Contamination	Pass
Western Blot	Comparable to CS6 Reference Standard	Comparable to CS6 Reference Standard	Pass
Antibiotic Sensitivity	For Information Use Only	@ 0.5 μg/ml Ciprofloxacin Sensitive	N/A

Abbreviations: N/A = Not Applicable, CFU = Colony Forming Units

This PCB of B7A was used previously used in a human challenge trial carried out by WRAIR investigators (under BB-IND 7766). Organisms prepared from the PCB will be used to challenge subjects participating in this trial. This strain is susceptible to ciprofloxacin, and amoxicillin.

8.3. *Product preparation*

Fresh, plate grown organisms will be used for challenge inocula, a standard approach for ETEC challenge studies. Approximately 48 hours before challenge, a vial of the cGMP PCB will be thawed and streaked onto agar for the inoculum and MacConkey for *E. coli* verification. After 22-24 hours of incubation at 35-37°C, 10 colonies will be used to prepare a suspension in sterile saline (0.85%). This suspension will be used to heavily inoculate approximately 6 agar plates for incubation at 35 -37°C. Agar plates will be harvested in sterile saline after 18 - 20 hours and the resulting bacterial suspension further diluted in saline for optical density determination at 600 nm. The optical density of the suspension will be adjusted to the appropriate concentration of bacterial cells depending on study group. The number of cfu in the inoculum will be determined by titrating and plating on agar plates before and after administration to subjects.

8.4. *Storage*

The B7A vials are stored at ≤-80°C ± 10°C.

The challenge strain will be transferred on dry ice from the WRAIR PBF to the CIR Enterics Research Laboratory at JHBSPH, logged in and stored at -80°C ± 10°C in a locked and temperature-monitored freezer. Any use of these vials will be done under the supervision of the CIR Enterics Research Laboratory, JHSPH and tracked in an accountability log. Any vials remaining at the end of the study will be disposed of or returned to NMRC for use in non-clinical research studies.

9. Subject withdrawal

Each subject may withdraw consent at any time during the study without penalty. Counseling about the subject's health will be provided if he/she decides to discontinue participation in the study. Medical advice regarding what is in the best interest of the subject will be provided.

The PI may discontinue the subject’s activity without the subject’s consent if any of these criteria are met:

- A subject fails to comply with study procedures.
- A subject's safety or health may be compromised by further participation.
- It is determined to be in the subject's best interest.

9.1. *When and how to withdraw subjects*

A subject may end his or her participation in the study at any time. If a subject withdraws, the investigator will make a reasonable effort to determine the reason for the withdrawal from the study and to complete termination procedures. Telephone calls, registered letters, and email correspondence are considered reasonable effort. For subjects leaving the study, a targeted examination may be performed, if medically indicated and if permitted by the subject. If subjects withdraw after receiving the challenge, they will be encouraged to remain an inpatient until two negative cultures are obtained and after completing two doses of antibiotic.

A subject may be withdrawn for an adverse event (AE) or serious adverse event (SAE) resulting in a safety concern, or for noncompliance with protocol requirements. When a subject withdraws due to an AE or is withdrawn by the PI due to an AE, the sponsor must be notified within 24 hours. Investigators must follow specific policy at each institution regarding the timely reporting of AEs and SAEs to the local IRB. In all cases, the PI will make a reasonable effort to complete study termination procedures.

9.2. *Replacement of subjects*

Up to 8 alternates per cohort may be selected. If a subject does not present for challenge, elects to withdraw, or is found to have met an exclusion criterion prior to challenge, s/he will be replaced by an alternate. Subjects who withdraw or are withdrawn after being challenged will not be replaced.

9.3. *Follow-up for withdrawn subjects*

If possible, attempts will be made to follow-up with the subjects for safety at least 28-days after receipt of the challenge inoculum. Immunogenicity assessments will be continued for all subjects presuming no undue risk to the subjects related to specimen collection. If a subject meets withdrawal conditions for a concomitant medication violation or noncompliance, this should be clearly documented. All withdrawn subjects will receive antibiotics for outpatient treatment and will be educated on the importance of complying with treatment.

10. Safety assessment

Safety monitoring will be conducted throughout the study; therefore, safety concerns will be identified by continuous review of the data by the PI, clinic staff, clinical monitor, research monitor, and the sponsor.

Study Safety Management: The research monitor and principal investigator will review any safety concern. A data safety monitoring board (DSMB) is not required for this study.

Research Monitor: The research monitor will function as an independent safety advocate for subjects per AR 70-25 and DoD Instruction 3216.02. An independent research monitor is required to review all unanticipated problems involving risk to subjects or others, SAEs, and all subject deaths associated with the protocol and provide an unbiased written report of the event. At a minimum the research monitor should comment on the outcomes of the event or problem and, in the case of a SAE or death, comment on the relationship to participation in the study. The research monitor should also indicate whether he/she concurs with the details of the report provided by the study investigator. Reports for events determined by either the investigator or research monitor to be possibly or definitely related to participation and reports of events resulting in death should be promptly forwarded to the IRBs, ORP HRPO, and USAMRMC Division of Regulated Activities and Compliance.

The research monitor, in accordance with JHBSPH guidelines, will have the following responsibilities:

- Evaluate ongoing safety data and make recommendations in order to ensure subjects safety as required
- Be available for consultation by the clinical investigative team through the period of the clinical study in which there is an interaction with human subjects
- Be available to review all SAEs and other unanticipated problems involving risk to subjects
- Be available to discuss SAEs and significant safety issues
- Provide clinical advice, in accordance with the study protocol, on the clinical management of subjects. This advice may include, but is not limited to
  - Decisions on “borderline” laboratory values and eligibility for enrollment
  - Confirmation and discussion of treatment decisions for difficult clinical situations
- Must document all clinical decisions including date, time and signature
- Must communicate all decisions to the study PI and other study investigators, which must be stored with subject source documents

All safety reports (ie, serious adverse events, deviations, unanticipated problems involving risk and subject deaths) will be submitted to the Western and NMRC IRBs.

10.1. *Vital signs*

Vital signs (temperature, blood pressure, heart rate) will be obtained throughout the inpatient period and at each study visit after discharge. Respiratory rates will be obtained on a case-by-case basis at the discretion of the study clinician. (See for applicable AE coding.)

Table 5. Reference Ranges and Adverse Event Coding for Vital Signs Parameters

Vital Signs	Mild (Grade 1)	Moderate (Grade 2)	Severe (Grade 3)	Potentially Life Threatening (Grade 4)
Heart rate				
Tachycardia	101–115	116-130	>130	ER visit or hospitalization for arrhythmia
Bradycardia	50-54[a]	45–49	<45	ER visit or hospitalization for arrhythmia

Vital Signs	Mild (Grade 1)	Moderate (Grade 2)	Severe (Grade 3)	Potentially Life Threatening (Grade 4)
Fever (°C) (°F)	≥100.4 °F and ≤101.1°F (38.0-38.4°C)	≥101.1°F and ≤102.0°F (38.5-38.9°C)	≥102.1 °F and ≤104 °F (39.0-40.0 °C)	> 104.0 °F; life threatening
Blood Pressure				
Hypertension (systolic, mm Hg)	141–150	151 - 155	>155	ER visit/hospitalization for malignant hypertension
Hypertension (diastolic, mm Hg)	91–95	96 – 100	>100	ER visit/hospitalization for malignant hypertension
Hypotension (systolic, mm Hg)[b]	85–89	80 – 84	<80	ER visit/hospitalization for hypotensive shock

[a] Grade 1 bradycardia will not be considered an abnormality for this study unless judged to be clinically significant by the PI or the PI in consultation with the Research Monitor and sponsor.
[b] If a subject has a baseline systolic BP in the 90's then a decrease in BP < 10 without associated clinical symptoms will not be considered an abnormality for this study unless judges to be clinically significant by the PI.

10.2. *Physical examination*

A complete physical exam will be conducted during the screening visit as part of the screening process; a targeted physical exam will be conducted prior to challenge and daily during subject's inpatient stay. Subsequent focused clinical assessments will occur at each study visit with specific attention to the identification of local, systemic or other adverse reactions.

10.3. *Laboratory assessments*

Venous blood samples will be collected for chemistry, hematology, and immunological parameters during the screening phase of this study and to provide a baseline sample. Hematology and chemistry analyses will be performed by commercial laboratory (Quest, Incorporated in Baltimore City or by Johns Hopkins Medical Institutions). Additional specimens may be collected to confirm and evaluate any abnormal values. Additional blood for chemistry and hematology are not planned for systematic collection following experimental infection. However, samples may be obtained as part of the clinical care of an individual subject. The clinical toxicity grading scale that will be used as a guideline is based on the Guidance for Industry: Toxicity Grading Scale for Healthy Adult and Adolescent Subjects enrolled in Preventive Vaccine Clinical Trials. Final grading determination will be made by the Principal Investigator based on normal lab values for the specific lab and clinical symptoms.

Serologic evidence of chronic HIV-1, HCV, and HBV infections will be obtained during the screening process. Evidence of infection will make a subject ineligible. Additional testing will not be performed as part of this study beyond second tier confirmatory tests on those with preliminary positive tests on ELISA after HIV and/or HCV serology (ie,-Western Blots, RIBA).
Targeted drug screenings are planned for this study at screening and at the discretion of the study clinician.

A serum sample for pregnancy testing (female subjects) will be collected at the screening visit and on day -1 prior to challenge. If results from the serum pregnancy test are not available at time of challenge a urine pregnancy test may be performed. A positive pregnancy test prior to challenge will result in disenrollment. Any subjects who become pregnant during the study will be removed from the study and followed until the end of their pregnancy. Procedures to be followed in the event a study participant becomes pregnant during the study period are outlined below.

Table 6. Reference Ranges and Adverse Event Coding for Clinical Hematology Parameters

**Test**	**Quest Normal**	**Mild (Grade 1)**	**Moderate (Grade 2)**	**Severe (Grade 3)**	**Potentially Life Threatening (Grade 4)**
Hemoglobin (g/dL)<br>(for screening purposes only)	M: LLN = 13.2<br>F: LLN = 11.7	M: 12.5-13.1<br>F: 11.0-11.7	M: 10.5-12.4<br>F: 9.5-10.9	M: 8.5-10.4<br>F: 8.0-9.4	M: <8.5<br>F: <8.0
Hemoglobin - decrease from lower limit of normal<br>(used to grade toxicity)		0.5-1.5	1.6-2.0	2.1-5.0	> 5.0
Neutrophils (cells/mm^3)	1,500-7,800	1,225-1,499	1,000-1,224	776-999	< 776
Leukocytes (white blood cells) (cells/mm^3)	3,800-10,800				
Leukopenia		2,500-3,799	1,500-2,499	1,000-1,499	< 1,000
Leukocytosis		10,801-15,000	15,001-20,000	20,001-25,000	> 25,000
Lymphocytes (cells/mm^3)	850-3,900	750-849	500-749	250-499	< 250
Eosinophils (cells/mm^3)	15-500	551-1,500	1,501-5,000	> 5,000	Hypereosinophilic
Platelets decreased – 10^3/mm^3	140-400	125-139	100-124	25-99	< 25

Table 7. Reference Ranges and Adverse Event Coding for Blood Chemistry Parameters

**Test**	**Quest Normal**	**Mild (Grade 1)**	**Moderate (Grade 2)**	**Severe (Grade 3)**	**Potentially Life Threatening (Grade 4)**
Sodium	135-146 (mmol/L)				
Hyponatremia		132-134	130-131	125-129	< 125
Hypernatremia		147-148	149-150	151-152	> 152
Potassium	3.5-5.5 (mmol/L)				
Hypokalemia		3.3-3.4	3.1-3.2	2.9-3.0	< 2.9
Hyperkalemia		5.6-5.7	5.8-5.9	6.0-6.1	> 6.2

Glucose, Random	65-139 (mg/dL)				
Hyperglycemia		140-155	156-200	> 200	Insulin requirements or hyperosmolar coma
Hypoglycemia		60-64	55-59	45-54	< 45
SGOT/AST (elevation)	M: 10-40 U/L<br>F: 10-30 U/L	M: 41-100<br>F: 31-75	M: 101-200<br>F: 76-150	M: 201-400<br>F: 151-300	M: > 400<br>F: > 300
SGPT/ALT (elevation)	M: 9-60 U/L<br>F: 6-40 U/L	M: 61-150<br>F: 41-100	M: 151-300<br>F: 101-200	M: 301-600<br>F: 201-400	M: > 600<br>F: > 400
BUN (elevation)	7-25	26-28	29-31	> 31	Requires dialysis
Creatinine (elevation)	M: 0.7-1.4<br>F: 0.5-1.1	M: 1.5-1.7<br>F: 1.2-1.7	M: 1.8-2.0<br>F: 1.8-2.0	M: 2.1-2.5<br>F: 2.1-2.5	M: >2.5<br>F: >2.5 or requires dialysis

10.4. *IND safety reporting*

The following terms, as defined by 21 CFR 312.32, apply to IND safety reporting.

10.4.1. Adverse event or suspected adverse reaction

Adverse event means any untoward medical occurrence associated with the use of a drug in humans, whether or not considered drug related.

Suspected adverse reaction means any adverse event for which there is a reasonable possibility that the drug caused the adverse event. For the purposes of IND safety reporting, “reasonable possibility” means there is evidence to suggest a causal relationship between the drug and the adverse event. Suspected adverse reaction implies a lesser degree of certainty about causality than adverse reaction, which means any adverse event caused by a drug.

10.4.2. Solicited and anticipated adverse events

A solicited AE is a predetermined event, which may reflect safety concerns related to the investigational product. This study involves challenge with live ETEC bacteria, and therefore the symptoms of ETEC infection are expected. The most common effects of wild-type ETEC infection are moderate to severe diarrhea (which may lead to dehydration and the need for oral or intravenous rehydration), and abdominal cramping, fever, nausea with or without vomiting, loss of appetite, headache, myalgia, and bloating may occur. The following ETEC-associated AEs will be solicited daily during the challenge phase:

1. Vomiting
2. Abdominal Pain
3. Bloating
4. Lightheadedness
5. Anorexia (poor appetite)

6. Generalized Myalgia
7. Arthralgias
8. Abdominal cramping
9. Constipation
10. Nausea
11. Malaise
12. Headache
13. Flatulence

The following will be documented via clinical assessments during the inpatient challenge phase:

1. Diarrhea
2. Hypovolemia
3. Fever (oral temperature ≥ 100.4° F)

10.4.3. Serious adverse event or serious suspected adverse reaction

An adverse event or suspected adverse reaction is considered "serious" if, in the view of either the investigator or sponsor, it results in any of the following outcomes:

- Death
- Life-threatening adverse event
- Inpatient hospitalization or prolongation of existing hospitalization
- Persistent or significant incapacity or substantial disruption of the ability to conduct normal life functions
- Congenital anomaly/birth defect (abortion, stillbirth and any malformation/disease must be reported as an SAE).

An adverse event or suspected adverse reaction is considered "life-threatening" if, in the view of either the investigator or sponsor, its occurrence places the patient or subject at immediate risk of death. It does not include an adverse event or suspected adverse reaction that, had it occurred in a more severe form, might have caused death.

Important medical events that may not result in death, be life-threatening, or require hospitalization may be considered serious when, based upon appropriate medical judgment, they may jeopardize the patient or subject and may require medical or surgical intervention to prevent one of the outcomes listed in this definition. Examples of such medical events include allergic bronchospasm requiring intensive treatment in an emergency room or at home, blood dyscrasias or convulsions that do not result in inpatient hospitalization, or the development of drug dependency or drug abuse.

10.4.4. Unexpected adverse event or unexpected suspected adverse reaction

An adverse event or suspected adverse reaction is considered "unexpected" if it is not listed in the investigator brochure or is not listed at the specificity or severity that has been observed; or, if an

investigator brochure is not required or available, is not consistent with the risk information described in the general investigational plan or elsewhere in the current application, as amended. For example, under this definition, hepatic necrosis would be unexpected (by virtue of greater severity) if the investigator brochure referred only to elevated hepatic enzymes or hepatitis. Similarly, cerebral thromboembolism and cerebral vasculitis would be unexpected (by virtue of greater specificity) if the investigator brochure listed only cerebral vascular accidents. “Unexpected,” as used in this definition, also refers to adverse events or suspected adverse reactions that are mentioned in the investigator brochure as occurring with a class of drugs or as anticipated from the pharmacological properties of the drug, but are not specifically mentioned as occurring with the particular drug under investigation.

10.4.5. Other adverse event

Other adverse events will be identified by the principal investigator during the evaluation of safety data. Significant adverse events of particular clinical importance, other than SAEs and those AEs leading to discontinuation of the subject from the study, will be classified as other adverse events. For each, a narrative may be written and included in the clinical study report.

10.5. *Relationship to investigational product*

The investigator must assign a relationship of each AE to the receipt of the investigational product. The investigator will use clinical judgment in conjunction with the assessment of a plausible biologic mechanism, a temporal relationship between the onset of the event in relation to receipt of the investigational product, and identification of possible alternate etiologies including underlying disease, concurrent illness or concomitant medications. The following guidelines should be used by investigators to assess the relationship of an AE to study product administration. Only a physician can make this determination.

The investigator will assess causality of all AEs as either ‘related’ or ‘unrelated’. Non-serious and serious adverse events will be evaluated as two distinct types of events given their different medical nature. If an event meets the criteria to be determined ‘serious’ it will be examined by the investigator to the extent possible to determine ALL contributing factors applicable to the event. Other possible contributors include:

- Underlying disease
- Other medication
- Protocol required procedure
- Other cause (specify)

At a later date, the adjudication committee will confirm the relationships between diarrheal output and investigational product receipt, as determined by the investigator.

10.6. *Recording of adverse events*

10.6.1. Methods / Timing for assessing, recording and analyzing safety endpoints

AEs, solicited AEs, and SAEs will be assessed at all study visits, documented in the source records, and recorded on the eCRFs using accepted medical terms and/or the diagnoses that accurately characterize the event. Solicited AE's will be recorded as individual events. Unsolicited AE may be recorded as a diagnosis. When a diagnosis is known, the AE term recorded on the eCRF will be the diagnosis rather than a constellation of symptoms. The investigator will assess all AEs for seriousness, relationship to investigational product, severity, and other possible etiologies. When an event has not resolved by the proscribed reporting period, it will be left open/without an end date on the AE eCRF and will be updated with end date or ongoing at visit.

The timeframe for the collection of AEs and SAEs begins at the time of experimental infection product through 28 days after receipt of the challenge strain. Additionally, subjects will be contacted by telephone approximately at 6 months after challenge to assess for any new onset SAEs or AEs of special interest mandated by the FDA.

10.6.2. Duration of follow-up of subjects after adverse events

Investigators are required to follow SAEs to resolution, even if this extends beyond the prescribed reporting period. Resolution is the return to baseline status or stabilization of the condition with the probability that it will become chronic. The SAE outcomes will be reported to the sponsor.

Investigators are not obligated to actively seek SAEs in former subjects; however, if a SAE, considered to be related to the investigational product is brought to the attention of the investigator *at any time* until closure of the study, the event will be reported.

Investigators should follow-up adverse events at least until the final study visit. This may include repeat safety laboratory analysis. Outcome should be assessed as:

- Resolved
- Resolved with sequelae
- Severity change (highest severity in a day will be recorded, if the severity on day 1 is mod, then mild and mod, it will be entered as moderate for the day only, then if on day 2 is mild, the moderate AE will stop and the AE will be reentered as mild)
- Ongoing at day 28
- Died
- Lost to follow up

10.6.3. Safety assessment

All AEs will be assessed for severity by the investigator. Inherent in this assessment is the medical and clinical consideration of all information surrounding the event including any medical intervention required. Each event will be assigned one of the following categories: mild, moderate, severe, or life-threatening. The criteria below may be used for any symptom not included in the grading scale. Any grade 4 (life-threatening) AE must be reported as an SAE.

The eCRF for AEs will reflect only the highest severity for continuous days an event occurred.

**Mild**	Grade 1	Does not interfere with routine activities; minimal level of discomfort
**Moderate**	Grade 2	Interferes with routine activities; moderate level of discomfort
**Severe**	Grade 3	Unable to perform routine activities; significant level of discomfort
**Potentially life-threatening**	Grade 4	Hospitalization or ER visit for potentially life-threatening event

FDA guidelines for toxicity will be followed; however, if a subject is evaluated in an emergency room for nonlife threatening illness or symptoms (ie, visits emergency department on weekend for mild problems because the physician's office is closed), the information from that visit will be reviewed and severity of the adverse event will be assessed according to the subject's clinical signs and symptoms.

As defined by the ICH guideline for GCP, the term "severe" is often used to describe intensity (severity) of a specific event (as in mild, moderate, or severe myocardial infarction); the event itself however, may be of relatively minor medical significance (such as severe headache). This is **not** the same as "serious", which is based on subject/event **outcome** or **action** criteria usually associated with events that pose a threat to a subject's life or functioning. Seriousness (not severity) serves as a guide for defining regulatory reporting obligations.

During the challenge phase of the study, ETEC disease-specific adverse events will be graded in accordance to the table below.

Table 8. Challenge Phase ETEC Infection Anticipated Adverse Event / Endpoint Assessments

**Adverse Event**	**Severity**[a]	**Parameter**
Diarrhea (based on highest output of loose/liquid stools in any 24-hour period)	1	Mild: 1-3 grade 3-5 stools totaling 200-400g
	2	Moderate:<br>4 to 5 loose/liquid stools or 401-800 g of loose/liquid stool
	3	Severe: 6 or more grade 3-5 stools totaling >800g
	4	Life threatening
Body temperature (t)	1	≥100.4P^{o}PF and ≤101.1P^{o}PF (38.0-38.4P^{o}PC)
	2	≥101.1P^{o}PF and ≤102.0P^{o}PF (38.5-38.9P^{o}PC)
	3	>102P.0°PF (39P.0°PC)
	4	Life threatening hyperthermia
Vomiting	1	One episode within a 24-hour period
	2	Two episodes within a 24-hour period
	3	More than two episodes with a 24-hour period
	4	Life threatening consequence of emesis
Other solicited (nausea, emesis, bloating, myalgia, arthralgias, abdominal pain, abdominal	1	Discomfort noted, but no disruption of normal daily activities; slightly bothersome; relieved with or without symptomatic treatment.

Adverse Event	Severity[a]	Parameter
cramping, malaise, bloating, headache, lightheadedness, constipation, hypovolemia, fevers, anorexia, flatulence) and non-solicited adverse events.	2	Discomfort sufficient to reduce or affect normal daily activity to some degree; bothersome; interferes with activities, only partially relieved with symptomatic treatment.
	3	Discomfort sufficient to reduce or affect normal daily activity considerably; prevents regular activities; not relieved with symptomatic treatment.
	4	Life threatening

[a]1=mild; 2=moderate; 3=severe; 4=life threatening.

10.7. *Reporting adverse events*

The PI will report all AEs to the sponsor and the local IRB in the appropriate safety, annual, and/or final reports. The NMRC staff in conjunction with the clinical site will draft annual and final clinical study reports and provide files to the sponsor for review and submission to the FDA.

10.7.1. Reporting serious and unexpected adverse events

10.7.1.1. Reporting to the sponsor

All SAEs and unexpected AEs must be reported promptly (within 72 hours) to the sponsor as per 21 CFR 312.64, whether or not the event is considered related to study product. Further, the investigator should comply with relevant study site SOPs on reporting SAEs.

The minimum information that the investigator will provide to the sponsor is specified in Table 10.

Table 9. Study Contacts for Reporting Serious Adverse Events

**Sponsor**	A. Louis Bourgeois, Ph.D., M.P.H.<br>Center for Immunization Research<br>Department of International Health<br>Johns Hopkins Bloomberg School of Public Health<br>624 N. Broadway, HH, Rm 205<br>Baltimore, MD 20215
**Institutional Review Board**	Western Institutional Review Board (IRB of record)<br>3535 7th Avenue SW<br>Olympia, WA 98502<br>Telephone: (800) 562-4789<br>Fax: (360) 252-2498<br>Email: clientservices@wirb.com
**Collaborating Institutional Review Board**	Naval Medical Research Center (NMRC) IRB<br>Research Services Directorate<br>Office of Research Administration<br>Code 025, Building 500, Rm 004<br>Silver Spring, MD<br>Telephone: 301-319-7276<br>Fax: 301-319-7277
**Research Monitor**	Jane L. Halpern, MD, MPH, DrPH<br>Department of International Health<br>Johns Hopkins Bloomberg School of Public Health<br>1219 Roundhill Road<br>Baltimore, MD 21218<br>Tel. 410-366-4823<br>Email: jhjp@comcast.net

Table 10. SAE Information to Be Reported to the Sponsor

**Notification Method**	**Information to be Provided**
Email or Telephone (within 72 hours)	IND number, sponsor study number, name of the investigational product, and investigator name and contact number
	Subject identification number
	SAE, onset date, date of investigational product administration, severity, relationship, and subject's current status
AND	
Email or Fax	Cover sheet or letter
	Adverse event case report form
	Serious adverse event report form
	Concomitant medication case report form or a list of concomitant medications
	Medical record progress notes including pertinent laboratory/diagnostic test results

Notification Method	Information to be Provided
Email or Telephone (within 72 hours)	IND number, sponsor study number, name of the investigational product, and investigator name and contact number
NOTE: When submitting SAE reports via email, the subject line of each email notification will read as follows: **SAFETY REPORT – IND # _____, Study #_____, Subject# _____, Event term: _____**	

In order to comply with regulations mandating sponsor notification of specified SAEs to the FDA within 7 calendar days, investigators must submit additional information as soon as it is available. The sponsor will report unexpected SAEs associated with the use of the challenge strain to the FDA as specified at 21 CFR 312.32 (c).
Investigators must follow all relevant regulatory requirements as well as specific policy at each institution regarding the timely reporting of SAEs to the local IRB and research monitor.

Reporting to the sponsor does not fulfill the investigator's duty to report all unanticipated problems involving risk to human subjects or others to the IRB. The PI will notify the local IRB and the research monitor.

10.7.1.2. Reporting to the IRB

Unanticipated problems involving risk to subjects or others, serious adverse events related to participation in the study and all subject deaths should be promptly reported by phone, email, or fax to the local Western Institutional Review Board (WIRB) and NMRC ORA. A written report will follow the initial notification.

Investigators are required to forward safety information provided by the sponsor's representative to the IRB. All SAEs will be reported to the WIRB according to WIRB guidelines.

WIRB Guidelines: WIRB Phone 800-562-4789; Fax 360-252-2498. Sites must use the WIRB Ten-Day Adverse Event Form. Investigators are required to report adverse events that fit the following criteria within 10 working days of the time the investigator becomes aware of them (see Table 11 for contact information):

- Event is UNANTICIPATED (an unanticipated event is any adverse experience where the nature, severity or frequency is not identified in the investigator brochure or described in the protocol. Events which are already cited in the investigator brochure or protocol are not unanticipated and do not have to be reported to the WIRB), and

- Event is POSSIBLY RELATED to the study design, procedures, or drug/device. If the AE is clearly not related to the study drug, device, procedures, or washout process, it would not represent a risk to other subjects in the research and, therefore, does not have to be reported to the WIRB.

Table 11. Contact Information

IRB	Telephone	Fax	Address
NMRC	301-319-7276	301-319-7277	500 Robert Grant Ave<br>Silver Spring, MD 20910
WIRB	800-562-4789	360-252-2498	P.O. Box 12029<br>Olympia, WA 98508-2029

10.7.2. Immediately reportable events

10.7.2.1. Pregnancy

Each pregnancy must be reported *immediately* **(within 72 hours of identification)** by email or fax to the sponsor and the IRB. The investigator must report any pregnancy on study subjects to the Research Monitor within 14 calendar days of learning of this occurrence.

Subjects who become pregnant after Day 0 through 3 months after the last study visit will be followed to term, and the following information will be gathered for outcome: date of delivery and health status of the mother and child including the child's gender, height, and weight. Complications and/or abnormalities should be reported including any premature terminations. A pregnancy is reported as an AE or SAE only when there is suspicion that the investigational product may have interfered with the effectiveness of contraception or there was a serious complication in the pregnancy including a spontaneous abortion or an elective termination for medical rationale.

A pregnancy outcome other than abortion, stillbirth, and any malformation/disease as well as follow-up of the infant must be reported by the Investigator within 14 days of learning of its occurrence using local site procedures.

10.7.2.2. AE-related withdrawal of consent

Any AE-related withdrawal of consent during the study must be reported *immediately* **(within 24 hours of identification)** by email or fax to the sponsor and the IRB.

10.7.2.3. Pending inspections/Issuance of reports

The knowledge of any pending compliance inspection/visit by the FDA, Office for Human Research Protections (Department of Health and Human Services), or other government agency concerning clinical investigation or research, the issuance of Inspection Reports, FDA Form 483, warning letters, or actions taken by any Regulatory Agencies including legal or medical actions and any instances of serious or continuing noncompliance with the regulations or requirements will be reported immediately to IRB and the sponsor.

10.7.3. IND reporting

10.7.3.1. Annual reports

The NMRC lead investigator will be responsible for the preparation of a detailed annual synopsis

of clinical activity, including adverse events, for submission to the sponsor. Each annual report will summarize IND activity for 1 year beginning approximately 3 months before the IND FDA anniversary date. The sponsor will notify the NMRC lead investigator of the due date with sufficient time for the NMRC lead investigator to assemble the required information.

10.7.3.2. Final clinical study report

A final study report will be prepared in accordance with "Guidance for Industry: Submission of Abbreviated Reports and Synopses in Support of Marketing Applications" and ICH E3 Guideline "Structure and Content of Clinical Study Reports" and provided to the sponsor for review and approval. The sponsor representative will use this report to prepare the final clinical study report for submission to the FDA. The investigative team will report all AEs to the sponsor and the local IRB in the appropriate safety, annual, and/or final reports.

10.8. *Safety Criteria for Stopping Doses*

The PI, along with the research monitor, may determine if certain events warrant discontinuation of challenges for all subjects in a cohort. If any of the additional following events occur, administration of the investigational product will be discontinued for all subjects in that cohort, and the PI and the research monitor will undertake a thorough review of the events:

- The occurrence of one or more serious adverse events (SAEs) determined to be related to the investigational product.
- One serious or unexpected AE evaluated by the PI, research monitor and sponsor determined to be an unacceptable risk to the health and safety of other subjects.
- Systemic allergic reaction, including but not limited to generalized urticaria, generalized petechiae, or erythema multiforme, occurring in two or more subjects in a group. Bronchospasm or anaphylaxis occurring in any subject.
- One or more grade 3 or greater laboratory abnormalities or SAE (attributed to the lab abnormality) thought to be related to the investigational product challenge (definitely, probably, or possibly) is identified.

Further challenge, in accordance with the protocol, may be resumed with the concurrence of the research monitor, sponsor, PI, and the FDA.

10.9. *Study Termination Criteria*

The PI, research monitor, NMRC IRB, WIRB, Sponsor, or FDA may stop or suspend the use of this product at any time.

11. Statistical considerations

11.1. *Introduction*

Safety, clinical outcomes, and immunogenicity data will be entered into an eCRF using software for data management. Data will be edited with standard strategies for range and consistency checks. AEs for all subjects will be included in the safety analysis.

11.2. *Sample size consideration*

The aim to down select an ETEC challenge strain dose with a ≥ 70% attack rate can be accomplished in a minimum of 7 subjects with a confidence interval of 29 - 96%. Increasing the number of subjects by 15 for the selected inoculum/fasting regimen (ie, Cohort 2) will provide greater confidence (50 - 89%) that the target 70% attack rate will be achieved in future applications of the challenge model.

A sample size of 15 subjects per arm in Cohort 2 provides an 80% power to detect a moderate-severe diarrhea risk difference of 55% presuming a minimum of a 70% attack rate in naïve subjects.

11.3. *Analysis*

During each day of the inpatient period, subjects will be monitored for loose stools (Grade 3-5 not meeting the diarrhea definition), diarrhea, nausea, vomiting, abdominal pain or cramps, fever, headache, abdominal tenderness, abdominal distention or otherwise abnormal abdominal exam. Along with solicited symptoms noted above. Vital signs will be taken 3 times a day or more, particularly if the subject meets the study definition for severe diarrhea. All AEs will be summarized and compared between dose groups. Safety data, including AEs, stool information, specified vital signs, and laboratory tests, will be listed by study subject. The planned statistical evaluation will is based on the proportion of subjects meeting prospectively defined clinical, microbiological and immunological endpoints. The attack rate will be calculated for all study groups, using the standard definition of: (# with endpoint / # receiving inoculum) x 100%. Summary tables will also be created to detail quantitative and temporal features of the illness such as diarrhea stool frequency and volume, maximum temperature observed, and time to illness and infection. Continuous variables will be analyzed using nonparametric statistics. In addition, tables will be prepared to list each commonly observed adverse event, the number of subjects who experienced an event at least once, and the rate of subjects with adverse event(s). Adverse events will be divided into defined severity grades (mild, moderate, severe and life-threatening).

Immunological outcomes will also be summarized in a tabular format and graphed to demonstrate kinetics of response. Qualitative (responder rates) and quantitative assessments (log transformed values) will be analyzed. Median increases (fold rises) of antibody concentrations and seroconversion rates will be calculated along with their 95% confidence intervals. Geometric mean titers will also be determined and presented with their 95% confidence intervals. All statistical tests will be interpreted in a two-tailed fashion using an *alpha* = 0.05.

12. Data handling and recordkeeping

The primary source document for this study will be the subject's clinical file. If separate research records are maintained by the investigator(s), the medical record and the research records will be considered the source documents for the purposes of auditing the study. The source documents will be retained at the site.

For this study, an EDC database system will be used for the collection of the study data in an electronic format. The EDC database system will be designed based on the protocol requirements, the approved eCRF layouts and specifications, and in accordance with 21 CRF Part 11. The eCRF layouts and specifications define and identify the applicable source data that will be collected and captured into the EDC database system. The applicable source data will be electronically transcribed by the site designee onto the eCRF (data entry screens) in the EDC database system. The investigator is ultimately responsible for the accuracy of the data transcribed on the eCRF. Data monitoring and management will be performed in the EDC database system by the study monitor and the designated Data Management group.

A detailed data management plan will be written and approved by the study team and the PI. The plan will be drafted prior to study initiation but will be finalized before study close-out and database lock.

13. Record and specimen archival

All records pertaining to this protocol will be stored in a locked filing cabinet at the NMRC Enteric Diseases Department per Navy regulations for up to 75 years (GCP minimum requirement of 15 years). Access to these records will be limited to researchers in the Enteric Disease Department at NMRC as well as those responsible for regulatory monitoring of data to include representatives of the DoD and JHU. A copy of study records will be made available to the Sponsor. The investigator will obtain permission from the sponsor in writing before destroying any study records and the sponsor will notify the investigator in writing when records can be destroyed. Relevant IRBs will be notified in writing prior to destruction of any research records.

Specimens will be stored indefinitely in the JHU or the ETEC laboratory at NMRC.

14. Obligations and roles of sponsor, investigator and study personnel

This study will be conducted using GCP and in accordance with all federal regulations regarding the protection of human participants in research including The Nuremberg Code, The Belmont Report, US 21 CFR Part 50 – Protection of Human Subjects, 32 CFR 219 (The Common Rule) and all regulations pertinent to the Department of Defense.

The investigators agree to conduct the research in strict accordance with this protocol, the ICH Guideline for Good Clinical Practice (CPMP/ICH/135/95), as well as in conformity with any federal, provincial or local regulations regarding the conduct of clinical studies. The sponsor and investigator must comply with all applicable regulations. In addition, the investigator must follow local and institutional requirements including, but not limited to, investigational product, clinical research, informed consent and IRB regulations. The Sponsor will provide notification to the investigator of protocol and amendment approvals by regulatory authorities when applicable. Except where the investigator's signature is specifically required, it is understood that the term "investigator" as used in this protocol and on CRFs refers to the investigator or appropriate study personnel that the investigator designates to perform a certain duty. The investigator is ultimately responsible for the conduct of all aspects of the study. Sub-investigators or other appropriate study personnel are eligible to sign for the investigator on designated CRFs.

15. Quality control and assurance

15.1. *QA/QC monitoring*

During the study, the investigator will maintain complete and accurate documentation for the study, including medical records, records detailing the progress of the study for each subject, laboratory reports, CRFs, signed informed consent forms for each study subject, drug disposition records, correspondence with the IRB, the study monitor and the sponsor, adverse event reports and information regarding subject discontinuation and completion of the study. All required study data will be clearly and accurately recorded by authorized study personnel in the CRFs. Only designated study site personnel shall record or change data in a CRF. During the study, the investigator will be responsible for the procurement of data and for quality of data recorded in the CRFs. Original observations entered directly into the CRFs are defined as source data. Study specific procedures detail how each form will be completed. The study monitor will ensure accuracy of the case report forms.

15.2. *Protocol deviation management*

All amendments to the protocol, consent form and/or questionnaires, including a change of PI, will be submitted to the WIRB and NMRC IRB for review and approval prior to implementation. Other-than-minimal-risk changes and all unanticipated major problems involving human subjects or others will be reported promptly to the IRBs, and no such changes will be made to the research without IRB approval unless necessary to eliminate apparent immediate hazards to human subjects. Minor minimal-risk deviations necessitated during the course of the trial will be made on site as needed, and documented for subsequent review within a reasonable time period. Deviations from the protocol that potentially impact on subject safety will be promptly reported to the Research Monitor, IRBs, and the Sponsor. Other deviations will be reported at the time of continuing review.

15.3. *Monitoring*

Sponsor monitoring responsibilities will be provided by EMMES. Monitoring will be conducted according to an approved monitoring plan, and according to applicable SOPs.

The study monitor shall be available for consultation with the investigator, and serves as liaison between the clinical study site and the Sponsor. The study monitor or other authorized representatives of the Sponsor may inspect all documents and records maintained by the investigator, including, but not limited to, medical records (office, clinic or hospital) and pharmacy records for the subject in this study. The clinical study site will permit access to such records. The investigator will obtain, as part of informed consent, permission for authorized representatives of the Sponsor, or regulatory authorities, to review, in confidence, any records identifying individuals in this clinical study.

The investigator will notify the Sponsor within 24 hours following contact by a regulatory agency. The investigator and study coordinator will be available to respond to reasonable requests and audit queries made by authorized representatives of regulatory agencies. The investigator will provide

the Sponsor with copies of all correspondence that may affect the review of the current study or his/her qualification as an investigator in clinical studies conducted by the Sponsor. The Sponsor will provide any needed assistance in responding to regulatory audits or correspondence. The investigator will permit independent auditors (employees of the Sponsor or an external company designated by the Sponsor) to verify source data validation of the regularly monitored clinical trial. The auditors will compare the entries in the CRFs with the source data, and evaluate the study site for its adherence to the clinical study protocol and GCP guidelines and applicable regulatory requirements.

The Sponsor will arrange local monitoring prior to beginning, at initiation, during the study, and at closeout by the study monitor or designee.

16. Human subjects protections considerations

16.1. *Risks / Benefit*

16.1.1. Risks

Naturally acquired illness caused by ETEC ranges from mild-to-severe watery diarrhea. Nausea, vomiting, abdominal cramping, headache, abdominal gurgling or gas, anorexia, fever, muscle and/or joint aches, and malaise, may occur. For most adults the illness is not life threatening but often leads to mild to moderate dehydration and significant inconvenience associated with loss of sleep and activity. Study facilities will have personnel and resources capable to manage diarrheal illness and potential complications. Side effects to the antibiotic (ciprofloxacin) used to treat the ETEC infection are possible.

Therapeutic antibiotics for use in this study are licensed approved medications that have been used extensively and shown to be very safe with only rare side effects. The most commonly reported side effects for ciprofloxacin are gastrointestinal symptoms (nausea, vomiting, and diarrhea) in as many as 5 persons in 100. Other reported symptoms in less than 1 person in a 100 include rash, dizziness, and headache. Rarely, allergic reactions to these medications have been observed. Ciprofloxacin is not recommended for use in pregnancy due to concerns of joint damage to the unborn child (based on studies in young animals). Pregnancy is exclusionary for study participation and is documented through testing prior to study interventions and provided discussion on methods to prevent pregnancy during study. Fluoroquinolones, including ciprofloxacin, are associated with an increased risk of tendonitis and tendon rupture in all ages. The risk of developing fluoroquinolone-associated tendonitis and tendon rupture is further increased in older patients usually over 60 years of age, in patients taking corticosteroid drugs, and in patients with kidney heart or lung transplants, all of whom are excluded from this study. *Clostridium difficile* associated diarrhea (CDAD/pseudomembranous colitis) has been reported with use of nearly all antibacterial agents.

Good nursing practices are performed during blood draws, which minimizes the risk to the subject. Hand-washing and sanitary disposal of feces (including pretreatment with bleach) are the main elements of personal hygiene and will minimize the spread by person-to-person infection; hand washing will be emphasized to the subjects and subjects will be instructed not to share food or

beverages. Subjects and staff will be trained in proper techniques of hand washing. Subjects will be instructed as to the importance of completing the 5-day course of antibiotics and this instruction will be documented. Risk of secondary transmission is highly unlikely due to antibiotic treatment and because subjects are required to submit two confirmed, consecutive negative stool samples prior to discharge.

There is a minimal risk of pain, hematoma or infection at the site of venipuncture. The maximum amount of blood drawn from a subject in total, and daily, will fall within applicable regulations.

There may be physical, psychological and social risks if subjects test positive for hepatitis B, hepatitis C and/or HIV. Subjects testing positive will be counseled and referred for treatment.

Medical records associated with this protocol are subject to provisions of the Privacy Act of 1974, 5 U.S.C., Section 552A, and AR 340-21. All data and medical information obtained about subjects will be considered privileged and held in confidence. Subjects will not be identified by name in any published report/presentation of the results. Complete confidentiality cannot be promised to subjects who are military personnel, because appropriate medical command authorities may require reporting information bearing on the health of their personnel. Representatives of the Sponsor, NMRC IRB, WIRB, or FDA may inspect the records of this research as part of their responsibility to oversee research and ensure protection of subjects. Study results and data may be published in scientific/medical journals; the identity of individual subjects will not be disclosed.

16.1.2. Risk mitigation strategies

Subjects will be questioned and examined daily for evidence of infection and diarrhea complications. Vital signs will be recorded at least three times per day. Based on prior studies, infected subjects tend to develop illness with incubation periods of approximately 1-3 days. Therapeutic benefit seems to be optimal if treatment is given within the first three days of symptom onset. The risk of diarrhea complications will be minimized by a conservative approach to timing of antibiotic administration well within an interval that has been shown to be efficacious as well as daily clinical monitoring. Stool output will be closely monitored. The plan will be to treat all subjects no later than day 5 post-dosing.

Aggressive fluid management will be undertaken to ensure the most common complication, dehydration, does not occur. The procedures to institute early oral and/or intravenous rehydration therapy are detailed above. In addition to rehydration therapy, prospectively defined criteria and procedures to institute early antibiotic therapy are also fully described above. In order to ensure clinical resolution and limit the potential for secondary spread upon discharge, predefined discharge criteria have been established. Subjects will be discharged from the inpatient phase of the study when clinical symptoms are resolved or resolving AND two consecutive stool cultures are negative for ETEC.

Systemic or severe gastrointestinal complications rarely occur with ETEC infection. The following clinical findings necessitate immediate consideration and management of complicated enteritis:

- Physical examination compatible with an acute abdomen

- Severe GI bleeding (any evidence of GI blood loss other than hemoccult positivity only, with evidence of hemodynamic instability, decrease in hemoglobin, hypovolemia)
- Sepsis (high fever: temp. >102ºF (39ºC), rigors, hemodynamic instability).

Any of these findings require prompt clinical management and discussion with the independent Research Monitor.

The ETEC strain has the potential for risk to both the environment and to the research personnel; however, the risk to the environment in regards to potential transmission outside of the JHU CIR facility is low. There is a minimal risk of acquiring ETEC infection associated with subject inoculum administration, patient care activities on the ward, or processing ETEC-infected stool. The risk to the environment will be reduced by ensuring that all human waste products from inpatients are disinfected with bleach prior to disposal, ensuring all subjects comply with discharge criteria (two consecutive negative stool cultures for ETEC), emphasizing importance of handwashing for subjects and staff, ensuring proper disposal/cleaning of linen, and cohorting subjects in the JHU CIR while shedding ETEC. Additionally, subjects will not be discharged until they are no longer shedding the challenge strain as per procedures outlined in the protocol.

Recent studies also suggest an increased risk of post-infectious irritable bowel syndrome (PI-IBS) following bacterial enteritis, though there are no studies specifically linking ETEC with these sequelae [28, 75, 76]. PI-IBS, a functional bowel disorder characterized by unexplained abdominal discomfort or pain associated with changes in normal bowel patterns, has been described in a recent systematic review to occur 6-7 times more frequently after an acute enteric infection compared to similar matched controls without such a history [77]. Subjects with prior history of abnormal bowel patterns who might be at higher risk of this post-infectious sequelae are excluded and predefined criteria to assure early treatment as appropriate also may further reduce risk of post-infectious sequelae and is likely to reduce the risk associated with PI-IBS given the positive association between diarrheal illness duration and PI-IBS risk [78, 79].

16.1.3. Benefits

There is no benefit that can be guaranteed to subjects for participating in this research study. However, there is potential societal benefit of the development of a product to prevent ETEC.

16.2. *Subject compensation*

Compensation for participation will occur as detailed below. Compensation will be provided only for completed study procedures designated for compensatory payment. If a Subject is eligible to participate in the investigational protocol after screening, and s/he completes all study visits, procedures and follows all the rules s/he will receive the following compensation:

If enrolled in the study, the Subject will be compensated for participation time and travel in this trial as follows:

- $80 total for screening (only if enrolled in the study or presents as an alternate)
- $2,000 for the inpatient period (as long as all study requirements are met)

- $80 for outpatient study visit: Days 28
- $60 for the follow up telephone contact: Day 180
- $400 bonus upon completion of inpatient phase and outpatient visits

The payment schedule is:
- $2,080 at the completion of the inpatient period (approx. Day 8)
- $480 on Day 28
- $60 after completion of the telephone contact follow up, Day 180

Maximum compensation is $2620 for participation in one admission.

If a subject is not eligible for discharge on day 8 because of illness or not having 2 consecutive negative stool culture results s/he will receive $200 per additional inpatient day. Subjects will not be paid for missed outpatient visits, and may forfeit some or all of their bonus as a result of missed visits or non-compliance.

Subjects who enroll in both cohorts (second cohort compensation):

- $2,000 for the inpatient period (as long as all study requirements are met)
- $80 for outpatient study visit: Days 28 post second challenge
- $400 bonus upon completion of inpatient phase and outpatient visit

The payment schedule is:
- $2,000 at the completion of the inpatient period (approx. Day 8)
- $480 on Day 28

Total compensation for completion of two inpatient stays is $5,100.

16.3. *Research related injury*

All study-related medical care will be provided to subjects without cost. Should a subject be injured as a direct result of participating in this research project, s/he will be provided medical care by the staff at the Walter Reed National Military Medical Center (or other military-affiliated medical center), at no cost to the subjects, for that injury. The subjects will not receive any injury compensation, only medical care. The subjects will not be compensated for care if s/he chooses to seek care from his/her own physician.

If a subject is injured because of participation in this research and is a DoD healthcare beneficiary (e.g., active duty in the military, military spouse or dependent), the subject is entitled to medical care for that injury within the DoD healthcare system, as long as the subject remains a DoD healthcare beneficiary. This care includes, but is not limited to, free medical care at Army hospitals or clinics.

If a subject is injured because of participation in this research and is not a DoD healthcare beneficiary, the subject is entitled to free medical care for that injury at an Army hospital or clinic. It cannot be determined in advance which Army hospital or clinic will provide care. If the subject receives care for research-related injuries outside of an Army hospital or clinic, the subject or the subject's insurance will be responsible for medical expenses.

During the challenge phase, subjects who require medical treatment beyond what can be provided safely at the CIR will be transferred to the Johns Hopkins Hospital for care. If a subject is injured during the study, the study doctor will help the subjects find medical care. Medical care at Johns Hopkins is open to all subjects as it is to all sick or injured people. Neither Johns Hopkins Bloomberg School of Public Health nor the John Hopkins Hospital have any plan to provide compensation to the subjects if they experience injury or other bad effects which are not the fault of the study doctors. Subjects will only be treated for injuries that are directly caused by the research study. In the event this occurs, the sponsor agrees to reimburse the Hospital for all reasonable expenses incurred by the Hospital in providing medical treatment and/or hospitalization reasonably necessary to address any injury to a Subject that, in the reasonable judgment of Hospital and Sponsor, occurs directly as a result of the administration of the IMPs or performance of study procedures in accordance with the Protocol, but only to the extent such expenses are not:

- the result of a foreseeable side effect as indicated in the Protocol
- reimbursed by (or submitted for reimbursement to) the Subject's insurance or any governmental program or other third-party payer providing medical or hospital coverage; provided, however, that this provision shall not obligate Hospital to submit such costs to the prospective Subject's insurance or any governmental program or other third-party payer coverage
- attributable to a failure of Hospital, or any of the Investigator Personnel, including PI, to adhere to the terms of the Protocol, Sponsor's written instructions or Applicable Law
- attributable to the negligence, recklessness or willful misconduct or omission of Hospital or any of its Investigator Personnel, including PI
- attributable to a pre-existing abnormal medical condition or underlying disease of the Subject or treatment that would have been provided to the Subject in the ordinary course notwithstanding participation in the study, or
- attributable to the failure of the Subject to follow the reasonable instructions of Investigator Personnel or Subject's physician.

Transportation to and from military hospitals or clinics will not be provided. No reimbursement is available if the subject incurs medical expenses to treat research-related injuries from outside or private providers. No compensation is available for research-related injuries. The subject is not waiving any legal rights. The subject should contact the PI if the subject believes he or she has sustained a research-related injury. The subject should contact the PI for any questions.

Requests for other benefits, such as compensation for lost time from work, are processed independently of this protocol. Military members retain the right to pursue military disability benefits, and Federal civilian employees retain the right to pursue relief through established workers compensation processes, but neither military disability benefits nor workers compensation benefits are guaranteed. The right of other parties to seek redress against the United States

Government is limited to that set forth by existing agency regulations and the Federal Tort Claims Act. The subject should understand that this does not constitute a waiver or release of legal rights. This issue is addressed in the informed consent and will be discussed with the subject by the investigator or designee before the subject signs the informed consent to participate in the study.

16.4. *Compensation for investigators*

There is no financial compensation for investigators in this study. All investigators will be required to complete a form for the disclosure of significant financial interest.

16.5. *Fair and equitable selection of subjects*

Subjects will not be discriminated against on the basis of race, sex, or religion. Due to the early stage of development of this investigational product, we have excluded individuals under 18 and women who are pregnant or nursing and we have excluded individuals who are over the age of 50 due to the frequency of exclusionary medical conditions. Any individual who is unable to consent due to any reason will not be included in this study.

16.6. *Informed consent*

The informed consent process and document(s) will be reviewed and approved by the NMRC IRB and the WIRB prior to initiation of the study. The consent document(s) will contain a full explanation of the possible risks, advantages, and alternate treatment options, and availability of treatment in the case of injury, in accordance with 21 CFR 50. The consent document indicates that by signature, the subject, or where appropriate, legal guardian, permits access to relevant medical records by the sponsor's representative and by representatives of the FDA. The sponsor's representative will submit a copy of the initial IRB- and sponsor's representative-approved consent form to the FDA and will maintain copies of revised consent documents that have been reviewed and approved by the IRB/ethics committee.

A written informed consent document, in compliance with 21 CFR Part 50, 32 CFR Part 219, the Belmont Principles will be signed by the subject before any study-related procedures are initiated for that subject. This consent document must be retained by the investigator as part of the study records. The investigators or their designees will present the protocol in lay terms to individual subjects. Questions on the purpose of the protocol, protocol procedures, and risks to the subjects will then be solicited. Any question that cannot be answered will be referred to the PI. The subject will be allowed to take the consent document home to consider and discuss it with others and return to the CIR at a later time to sign it. The subject should understand that the study product is investigational and is not licensed by the FDA for commercial use, but is permitted to be used in this clinical research. Informed consent includes the principle that it is critical the subject be informed about the principal potential risks and benefits. This information will allow the subject to make a personal risk versus benefit decision and understand the following:

- Participation is entirely voluntary,
- Subjects may withdraw from participation at any time,
- Refusal to participate involves no penalty, and

- The individual is free to ask any questions that will allow him/her to understand the nature of the protocol.

A description of this clinical trial will be available on http://www.ClinicalTrials.gov, as required by US law.

All non-exempt research involving human subjects shall, at a minimum, meet the requirement of 32 CFR 219.116(a)(6) in the Code of Federal Regulations.

17. Privacy and confidentiality

17.1. *Storage of data and samples*

All original records involving this protocol will be stored at JHU CIR for 5 years. Copies of databases will be stored at NMRC for 75 years (and made available to the Sponsor). All samples will be stored under appropriate conditions in laboratories in the Enteric Disease Department at NMRC.

17.2. *Provisions protecting privacy and confidentiality*

Individual subject medical information obtained as a result of this study is considered confidential and disclosure to third parties, other than those cited below, is prohibited. Subject confidentiality will be further ensured by utilizing subject identification code numbers and subject initials. Neither NMRC nor the JHSPH are HIPAA-covered entities.

17.3. *Safeguards for vulnerable subjects*

This study will not include individuals less than 18, incarcerated or unable to meet the requirements to sign the informed consent form. Military personal will not be specifically recruited for this study.

18. Protocol review process

The protocol will undergo scientific and ethical review at the two primary collaborating institutions: JHU CIR and NMRC. In addition to these reviews, the JHU Biosafety Committee and Pharmacy and Therapeutics Committee will review the protocol. The protocol will also require FDA review as part of the Investigational New Drug (IND) application. The IND sponsor will be Dr. Louis Bourgeois. Continuing review will be undertaken in accordance with existing regulations.

The investigator may deviate from the protocol without prior approval when the change is necessary to eliminate an apparent immediate hazard to the subject. In that event, the investigator will notify the sponsor promptly by phone, will notify the JHU CIR (WIRB) and NMRC IRB, and will confirm notification to the sponsor in writing within 5 working days after the change is implemented. All protocol deviations, including minor deviations not impacting subject safety, will be noted in the continuing review reports, the annual report to the Sponsor, and in the final study report. Any modification to the protocol, consent form and/or questionnaires, including changing the PI, must be submitted to both IRBs for review and approval prior to implementation of the modification.

11 Publication Policy

All data collected during this study will be used to support this IND. All publications and presentations are governed by the standards and norms detailed in NAVMEDRSCHCENINST 5721.1. All authors will submit the proposed publication/presentation at least 30 days prior to the submission date. Prior to submission, the directorate will conduct a substantive scientific and professional review. The document is routed to the Office of Research Administration (ORA) for review and routing for Command review and approval, ultimately by the NMRC Public Affairs Officer (PAO). Once it is cleared at NMRC, it will be forwarded to BUMED through NMSC, if appropriate. Prior to publication, an author must have a completed Publication Clearance Request Submission Form with signatures from all approving and reviewing authorities.

12 List of References

1. *Diarrheal Fact Sheet*. 2013; Available from: http://www.who.int/mediacentre/factsheets/fs330/en/.
2. Sadeghabadi, A.F., et al., *Widespread antibiotic resistance of diarrheagenic Escherichia coli and Shigella species.* J Res Med Sci, 2014. **19**(Suppl 1): p. S51-5.
3. Steffen, R., et al., *Epidemiology of diarrhea in travelers.* JAMA, 1983. **249**(9): p. 1176-80.
4. Ericsson, C.D., *Travellers' diarrhoea.* Int J Antimicrob Agents, 2003. **21**(2): p. 116-24.
5. Kamat, D. and A. Mathur, *Prevention and management of travelers' diarrhea.* Dis Mon, 2006. **52**(7): p. 289-302.
6. Ryan, E.T. and K.C. Kain, *Health advice and immunizations for travelers.* N Engl J Med, 2000. **342**(23): p. 1716-25.
7. Lamberti, L.M., C.L. Fischer Walker, and R.E. Black, *Systematic review of diarrhea duration and severity in children and adults in low- and middle-income countries.* BMC Public Health, 2012. **12**: p. 276.
8. Hillel, O. and I. Potasman, *Correlation between adherence to precautions issued by the WHO and diarrhea among long-term travelers to India.* J Travel Med, 2005. **12**(5): p. 243-7.
9. Mattila, L., et al., *Risk Behavior for Travelers' Diarrhea Among Finnish Travelers.* J Travel Med, 1995. **2**(2): p. 77-84.
10. Tribble, D.R., et al., *Traveler's diarrhea in Thailand: randomized, double-blind trial comparing single-dose and 3-day azithromycin-based regimens with a 3-day levofloxacin regimen.* Clin Infect Dis, 2007. **44**(3): p. 338-46.
11. Rendi-Wagner, P. and H. Kollaritsch, *Drug prophylaxis for travelers' diarrhea.* Clin Infect Dis, 2002. **34**(5): p. 628-33.
12. Shlim, D.R., *Looking for evidence that personal hygiene precautions prevent traveler's diarrhea.* Clin Infect Dis, 2005. **41 Suppl 8**: p. S531-5.
13. Diemert, D.J., *Prevention and self-treatment of traveler's diarrhea.* Clin Microbiol Rev, 2006. **19**(3): p. 583-94.
14. Shah, N., H.L. DuPont, and D.J. Ramsey, *Global etiology of travelers' diarrhea: systematic review from 1973 to the present.* Am J Trop Med Hyg, 2009. **80**(4): p. 609-14.
15. Monteville, M.R., et al., *Incidence, etiology, and impact of diarrhea among deployed US military personnel in support of Operation Iraqi Freedom and Operation Enduring Freedom.* Am J Trop Med Hyg, 2006. **75**(4): p. 762-7.
16. Riddle, M.S., et al., *Incidence, etiology, and impact of diarrhea among long-term travelers (US military and similar populations): a systematic review.* Am J Trop Med Hyg, 2006. **74**(5): p. 891-900.
17. Hyams, K.C., et al., *Diarrheal disease during Operation Desert Shield.* N Engl J Med, 1991. **325**(20): p. 1423-8.
18. Kotloff, K.L., et al., *Burden and aetiology of diarrhoeal disease in infants and young children in developing countries (the Global Enteric Multicenter Study, GEMS): a prospective, case-control study.* Lancet, 2013. **382**(9888): p. 209-22.
19. Qadri, F., et al., *Disease burden due to enterotoxigenic Escherichia coli in the first 2 years of life in an urban community in Bangladesh.* Infect Immun, 2007. **75**(8): p. 3961-8.
20. Rao, M.R., et al., *High disease burden of diarrhea due to enterotoxigenic Escherichia coli among rural Egyptian infants and young children.* J Clin Microbiol, 2003. **41**(10): p. 4862-4.
21. Murray, C.J., et al., *Disability-adjusted life years (DALYs) for 291 diseases and injuries in 21 regions, 1990-2010: a systematic analysis for the Global Burden of Disease Study 2010.* Lancet, 2012. **380**(9859): p. 2197-223.

22. Vos, T., et al., *Years lived with disability (YLDs) for 1160 sequelae of 289 diseases and injuries 1990-2010: a systematic analysis for the Global Burden of Disease Study 2010.* Lancet, 2012. **380**(9859): p. 2163-96.
23. Connor, P., et al., *Diarrhoea during military deployment: current concepts and future directions.* Curr Opin Infect Dis, 2012. **25**(5): p. 546-54.
24. Riddle, M.S., et al., *A prospective study of acute diarrhea in a cohort of United States military personnel on deployment to the Multinational Force and Observers, Sinai, Egypt.* Am J Trop Med Hyg, 2011. **84**(1): p. 59-64.
25. Connor, P. and M.J. Farthing, *Travellers' diarrhoea: a military problem?* J R Army Med Corps, 1999. **145**(2): p. 95-101.
26. Putnam, S.D., et al., *Self-reported description of diarrhea among military populations in operations iraqi freedom and enduring freedom.* J Travel Med, 2006. **13**(2): p. 92-9.
27. Sanders, J.W., et al., *Impact of illness and non-combat injury during Operations Iraqi Freedom and Enduring Freedom (Afghanistan).* Am J Trop Med Hyg, 2005. **73**(4): p. 713-9.
28. Connor, B.A. and M.S. Riddle, *Post-infectious sequelae of travelers' diarrhea.* J Travel Med, 2013. **20**(5): p. 303-12.
29. Sanchez, J.L., et al., *Diarrheal disease incidence and morbidity among United States military personnel during short-term missions overseas.* Am J Trop Med Hyg, 1998. **58**(3): p. 299-304.
30. Sanders, J.W., et al., *Military importance of diarrhea: lessons from the Middle East.* Curr Opin Gastroenterol, 2005. **21**(1): p. 9-14.
31. Gaastra, W. and A.M. Svennerholm, *Colonization factors of human enterotoxigenic Escherichia coli (ETEC).* Trends Microbiol, 1996. **4**(11): p. 444-52.
32. Fleckenstein, J.M., et al., *Molecular mechanisms of enterotoxigenic Escherichia coli infection.* Microbes Infect, 2010. **12**(2): p. 89-98.
33. Sears, C.L. and J.B. Kaper, *Enteric bacterial toxins: mechanisms of action and linkage to intestinal secretion.* Microbiol Rev, 1996. **60**(1): p. 167-215.
34. Zhang, R.G., et al., *The three-dimensional crystal structure of cholera toxin.* J Mol Biol, 1995. **251**(4): p. 563-73.
35. Low, D.B., B. Van der Woude, M. , *Escherichia coli and Salmonella; Fimbriae*, in *Cellular and Molecular Biology*. 1996, ASM Press: Washington, D.C. p. 146-157.
36. Anantha, R.P., et al., *Evolutionary and functional relationships of colonization factor antigen i and other class 5 adhesive fimbriae of enterotoxigenic Escherichia coli.* Infect Immun, 2004. **72**(12): p. 7190-201.
37. Sakellaris, H., G.P. Munson, and J.R. Scott, *A conserved residue in the tip proteins of CS1 and CFA/I pili of enterotoxigenic Escherichia coli that is essential for adherence.* Proc Natl Acad Sci U S A, 1999. **96**(22): p. 12828-32.
38. Gaastra, W., et al., *Antigenic variation within the subunit protein of members of the colonization factor antigen I group of fimbrial proteins in human enterotoxigenic Escherichia coli.* Int J Med Microbiol, 2002. **292**(1): p. 43-50.
39. Thomas, L.V., et al., *The possession of three novel coli surface antigens by enterotoxigenic Escherichia coli strains positive for the putative colonization factor PCF8775.* J Gen Microbiol, 1985. **131**(9): p. 2319-26.
40. Isidean, S.D., et al., *A systematic review of ETEC epidemiology focusing on colonization factor and toxin expression.* Vaccine, 2011. **29**(37): p. 6167-78.
41. Clemens, J.D., et al., *Cross-protection by B subunit-whole cell cholera vaccine against diarrhea associated with heat-labile toxin-producing enterotoxigenic Escherichia coli: results of a large-scale field trial.* J Infect Dis, 1988. **158**(2): p. 372-7.
42. Cravioto, A., et al., *Prospective study of diarrhoeal disease in a cohort of rural Mexican children:*

incidence and isolated pathogens during the first two years of life. Epidemiol Infect, 1988. **101**(1): p. 123-34.
43. Levine, M.M., Nalin, D.R., Hoover, D.L., Bergquist, E.J., Hornick, R.B. & Young, C.R., *Immunity to enterotoxigenic Escherichia coli.* Infect. Immun., 1979. **23**: p. 729-36.
44. Lopez-Vidal, Y., et al., *Enterotoxins and adhesins of enterotoxigenic Escherichia coli: are they risk factors for acute diarrhea in the community?* J Infect Dis, 1990. **162**(2): p. 442-7.
45. Qadri, F., et al., *Prevalence of toxin types and colonization factors in enterotoxigenic Escherichia coli isolated during a 2-year period from diarrheal patients in Bangladesh.* J Clin Microbiol, 2000. **38**(1): p. 27-31.
46. Moon, H.W. and T.O. Bunn, *Vaccines for preventing enterotoxigenic Escherichia coli infections in farm animals.* Vaccine, 1993. **11**(2): p. 213-200.
47. Snodgrass, D.R., Nagy, L.K., Sherwood, D. & Campbell, I., *Passive immunity in calf diarrhea: vaccination with K99 antigen of enterotoxigenic Escherichia coli and rotavirus.* Infect. Immun., 1982. **37**: p. 586-91.
48. Tacket, C.O., et al., *Protection by milk immunoglobulin concentrate against oral challenge with enterotoxigenic Escherichia coli.* N Engl J Med, 1988. **318**(19): p. 1240-3.
49. Freedman, D.J., et al., *Milk immunoglobulin with specific activity against purified colonization factor antigens can protect against oral challenge with enterotoxigenic Escherichia coli.* J Infect Dis, 1998. **177**(3): p. 662-7.
50. Jiang, Z.D., et al., *Prevalence of enteric pathogens among international travelers with diarrhea acquired in Kenya (Mombasa), India (Goa), or Jamaica (Montego Bay).* J Infect Dis, 2002. **185**(4): p. 497-502.
51. Shaheen, H.I., et al., *Phenotypic and genotypic analysis of enterotoxigenic Escherichia coli in samples obtained from Egyptian children presenting to referral hospitals.* J Clin Microbiol, 2009. **47**(1): p. 189-97.
52. Willshaw, G.A., et al., *Isolation of enterotoxigenic Escherichia coli from British troops in Saudi Arabia.* Epidemiol Infect, 1995. **115**(3): p. 455-63.
53. Wolf, M.K., et al., *Characterization of enterotoxigenic Escherichia coli isolated from U.S. troops deployed to the Middle East.* J Clin Microbiol, 1993. **31**(4): p. 851-6.
54. Dutta, S., et al., *Trends in the prevalence of diarrheagenic Escherichia coli among hospitalized diarrheal patients in Kolkata, India.* PLoS One, 2013. **8**(2): p. e56068.
55. Mansour, A., et al., *Pathogenicity and phenotypic characterization of enterotoxigenic Escherichia coli isolates from a birth cohort of children in rural Egypt.* J Clin Microbiol, 2014. **52**(2): p. 587-91.
56. Nada, R.A., et al., *Phenotypic and genotypic characterization of enterotoxigenic Escherichia coli isolated from U.S. military personnel participating in Operation Bright Star, Egypt, from 2005 to 2009.* Diagn Microbiol Infect Dis, 2013. **76**(3): p. 272-7.
57. Rivera, F.P., et al., *Genotypic and phenotypic characterization of enterotoxigenic Escherichia coli strains isolated from Peruvian children.* J Clin Microbiol, 2010. **48**(9): p. 3198-203.
58. Guerena-Burgueno, F., et al., *Safety and immunogenicity of a prototype enterotoxigenic Escherichia coli vaccine administered transcutaneously.* Infect Immun, 2002. **70**(4): p. 1874-80.
59. Harro, C., et al., *A combination vaccine consisting of three live attenuated enterotoxigenic Escherichia coli strains expressing a range of colonization factors and heat-labile toxin subunit B is well tolerated and immunogenic in a placebo-controlled double-blind phase I trial in healthy adults.* Clin Vaccine Immunol, 2011. **18**(12): p. 2118-27.
60. Katz, D.E., et al., *Oral immunization of adult volunteers with microencapsulated enterotoxigenic Escherichia coli (ETEC) CS6 antigen.* Vaccine, 2003. **21**(5-6): p. 341-6.
61. Lapa, J.A., et al., *Randomized clinical trial assessing the safety and immunogenicity of oral microencapsulated enterotoxigenic Escherichia coli surface antigen 6 with or without heat-labile*

enterotoxin with mutation R192G. Clin Vaccine Immunol, 2008. **15**(8): p. 1222-8.
62. Tobias, J., et al., *Construction of a non-toxigenic Escherichia coli oral vaccine strain expressing large amounts of CS6 and inducing strong intestinal and serum anti-CS6 antibody responses in mice.* Vaccine, 2011. **29**(48): p. 8863-9.
63. Qadri, F., et al., *Mucosal and systemic immune responses in patients with diarrhea due to CS6-expressing enterotoxigenic Escherichia coli.* Infect Immun, 2007. **75**(5): p. 2269-74.
64. Alam, M.M., et al., *Antigen-specific memory B-cell responses to enterotoxigenic Escherichia coli infection in Bangladeshi adults.* PLoS Negl Trop Dis, 2014. **8**(4): p. e2822.
65. Coster, T.S., et al., *Immune response, ciprofloxacin activity, and gender differences after human experimental challenge by two strains of enterotoxigenic Escherichia coli.* Infect Immun, 2007. **75**(1): p. 252-9.
66. Rao, M.R., et al., *Serologic correlates of protection against enterotoxigenic Escherichia coli diarrhea.* J Infect Dis, 2005. **191**(4): p. 562-70.
67. Nicklasson, M., et al., *Expression of colonization factor CS5 of enterotoxigenic Escherichia coli (ETEC) is enhanced in vivo and by the bile component Na glycocholate hydrate.* PLoS One, 2012. **7**(4): p. e35827.
68. DuPont, H.L., et al., *Pathogenesis of Escherichia coli diarrhea.* N Engl J Med, 1971. **285**(1): p. 1-9.
69. Porter, C.K., et al., *A systematic review of experimental infections with enterotoxigenic Escherichia coli (ETEC).* Vaccine, 2011. **29**(35): p. 5869-85.
70. Levine, M.M., et al., *Immunity to enterotoxigenic Escherichia coli.* Infect Immun, 1979. **23**(3): p. 729-36.
71. Harro, C., et al., *Refinement of a human challenge model for evaluation of enterotoxigenic Escherichia coli vaccines.* Clin Vaccine Immunol, 2011. **18**(10): p. 1719-27.
72. Harris, J.B., et al., *Blood group, immunity, and risk of infection with Vibrio cholerae in an area of endemicity.* Infect Immun, 2005. **73**(11): p. 7422-7.
73. Kambhampati, A., et al., *Host Genetic Susceptibility to Enteric Viruses: A Systematic Review and Metaanalysis.* Clin Infect Dis, 2016. **62**(1): p. 11-8.
74. Cooling, L., *Blood Groups in Infection and Host Susceptibility.* Clin Microbiol Rev, 2015. **28**(3): p. 801-70.
75. Thabane, M. and J.K. Marshall, *Post-infectious irritable bowel syndrome.* World J Gastroenterol, 2009. **15**(29): p. 3591-6.
76. Halvorson, H.A., C.D. Schlett, and M.S. Riddle, *Postinfectious irritable bowel syndrome--a meta-analysis.* Am J Gastroenterol, 2006. **101**(8): p. 1894-9; quiz 1942.
77. Thabane, M., D.T. Kottachchi, and J.K. Marshall, *Systematic review and meta-analysis: The incidence and prognosis of post-infectious irritable bowel syndrome.* Aliment Pharmacol Ther, 2007. **26**(4): p. 535-44.
78. Smith, J.L. and D. Bayles, *Postinfectious irritable bowel syndrome: a long-term consequence of bacterial gastroenteritis.* J Food Prot, 2007. **70**(7): p. 1762-9.
79. Spiller, R.C., *Role of infection in irritable bowel syndrome.* J Gastroenterol, 2007. **42 Suppl 17**: p. 41-7.
